# PolyWAG: Autonomous filtered water sampling for eDNA

**DOI:** 10.1016/j.ohx.2024.e00617

**Published:** 2024-12-30

**Authors:** Riley Prince, Kai Roy, Nathan Jesudason, Marc Belinga, Jacob Field, Dylan Heiesy, Aaron Arvidson, Torrey Menne, John Selker, Chet Udell

**Affiliations:** OPEnS Lab, Oregon State University, Corvallis, OR, United States

**Keywords:** Environmental DNA, Sampling, Arduino, Data logging

## Abstract

Environmental DNA (eDNA) is an ideal way of researching aquatic environments and determining what species are present in an area the biodiversity of an area, and if any invasive or endangered species are present. Traditional sampling of eDNA consists of manually filtering water, which is labor and cost-intensive for remote locations. Furthermore, commercialized solutions are either expensive or require a field operator to function. We have built a battery-powered eDNA sampler capable of autonomous multi-sampling for a greatly reduced price compared to existing technologies. Environmental DNA collection contains 3 main components: environmental DNA must be preserved, the filtered volume must be accurate, and there must be no cross-contamination between samples. The sampler operates in this way separating eDNA via filters, preserving DNA, and recording the filtered volume per sample. Our PolyWAG eDNA sampler system is a water sampling device that collects DNA samples via 47 mm filter and provides a non-invasive, safe and autonomous means of eDNA collection. The sampler can hold 24 filters and is designed to be easily replaced and reusable. A browser application is used for real-time monitoring, scheduling tasks, and data logging for time, pressure, flow, and filtered volume. Additionally, the sampler design is openly published, modular and is constantly being tested to help us optimize our software and hardware to give us the best results. The 13-step sampling sequence helps reduce cross contamination significantly. Our machine can be deployed for an extended period. It is completely autonomous and costs around $3800 for components or $6000 including labor.

## Graphical abstract

**Figure:**
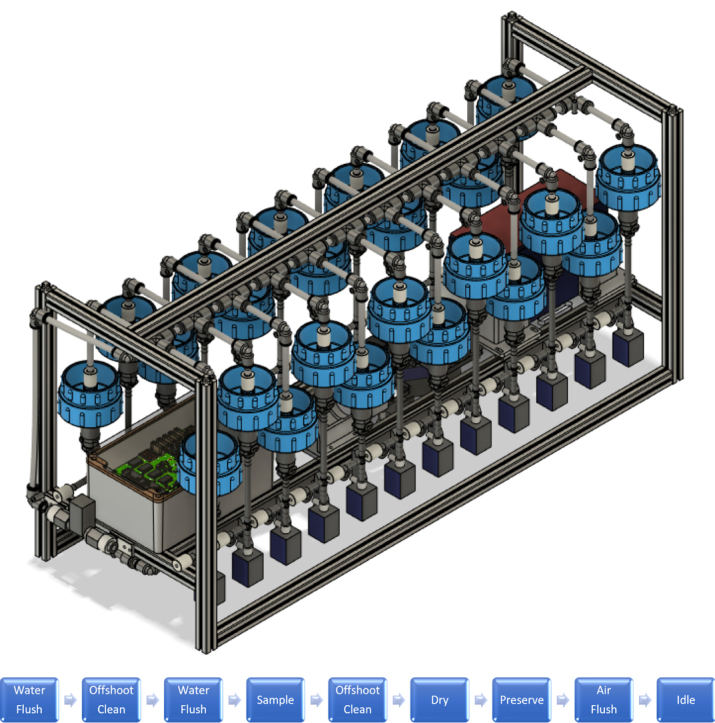

**Specifications table**

**Table:** 

**Hardware name**	*PolyWAG*

**Subject area**	*Environmental, planetary and agricultural sciences*

**Hardware type**	*Field measurements and sensors*

**Closest commercial analog**	*Dartmouth Ocean Technologies’ eDNA Sampler*

**Open source license**	CERN Open Hardware Licence Version 2 - Strongly Reciprocal GNU AFFERO GENERAL PUBLIC LICENSE Version 3

**Cost of hardware**	*$3800 (Cost of just components)* *$6000 (Cost with labor included)*

**Source file repository**	https://doi.org/10.5281/zenodo.14160133

## 1 Hardware in context

Environmental DNA (eDNA) is DNA derived from mucus, feces, gametes, and carcasses [1]. Many things can be learned once this DNA is put through sequencing. eDNA can be used to determine what species are present in an area, the biodiversity of an area, and if any invasive or endangered species are present [2]. eDNA sampling provides scientists and researchers a non-invasive, rapid, cost-effective and sensitive way to detect and estimate the numbers of those organisms present. The use of eDNA is diverse, with many fields leveraging this biological information to understand environments. These disciplines include passive surveillance (Whale tracking), abundance estimates (fish spawning), invasion biology (invasive species detection), environmental assessments (pollen detection), and many more. In recent years its use has exploded, with the number of eDNA related publications increasing nearly 200 times from 2008 to 2019 [3].

Traditional sampling of environmental DNA consists of manually filtering water, often requiring one or more researchers to be on location for days or weeks [4]. The filtration process varies depending on the researcher, but it is common to pull a sample of water with a bottle and pour that water into a funnel containing a filter. This can be connected to a vacuum pump to expedite the filtering process. After the sampling process is completed, the filters need to be preserved and the setup cleaned to avoid cross contamination [4]. This process is labor intensive, cost intensive, and can be dangerous, especially for remote locations. While commercialized solutions to this problem exist, they either still require an operator to be on location or are very expensive. commercial solutions offer a simplified process with additional data collection such as GPS location for around $8,000 [5]. A disadvantage of this solution is that it is not fully autonomous, still requiring an operator to be on location to use the device [5]. An alternative is the DOT Sampler which is a fully autonomous solution that is capable of multiple samples (20+ samples) and is also submersible but comes at a cost of ∼$55,000 [6].

The solution designed by the OPEnS Lab is the middle ground of these two solutions. Environmental DNA collection contains 3 main components: environmental DNA must be preserved, the filtered volume must be accurate, and the must be no cross-contamination between samples. The sampler operates in this way, separating eDNA via filters, preserving DNA, and recording the filtered volume per sample. While it is not submersible (limiting its potential sampling environments), it is capable of autonomous, multi-sample operations for extended periods of time (approximately one month) for the cost of $3800 for components or $6000 including labor. The two core priorities for our design are its autonomous function and the cross-contamination. The autonomous function of the sampler is important for a handful of reasons. An autonomous system requires less researcher hours spent in the field. This has cost benefits from the reduced hours worked and safety benefits when sampling in hazardous environments.

## 2 Hardware description

The eDNA sampler we have developed is a battery-powered autonomous multi-sampling device that collects eDNA samples from water via 47 mm filter holders and provides a non-invasive, safe, and autonomous means of DNA collection. The Sampler Hardware was designed using Autodesk Fusion 360. This design enables the use of 24 replaceable and reusable filter housings. The sampler operates through a custom logic board designed using Eagle CAD. This board is controlled with an Adafruit M0 Feather Wi-Fi microcontroller loaded with a webserver to act as the interface for the sampler’s operations. This webserver hosts a browser application which is used for real-time monitoring, scheduling tasks, and data logging for time, pressure, temperature, flow, and sample volume. The data is located stored onto an SD Card for later data analysis.

The basic function of the sampler can be split into five main sections: Hydraulics, Sampling Procedure, Utilities, Electronics, and User Interface. The Hydraulics section describes how the sampler is physically arranged, the different devices connected to the system, and provides a general idea on how liquids flow through the system. This section is supported by the Sampling Procedure Section which covers what the PolyWAG sampler is doing during each stage of the process. The Sampling Procedure Section refers to the devices described in the Hydraulics section in order to describe what is happening in each state as well as the purpose of that state. The Utilities section is similar to the Sampling Procedure section, but covers the four main utility functions of the sampler. These are functions that are used by an operator during cleaning or setup of the sampler. The Electronics section gives a brief overview of how the custom control board functions. Finally, the User Interface sections gives a brief overview on what the can be found and one within in the UI. More specific details on the User Interface can be found in the Operation Instructions section

**Fig. 1:**
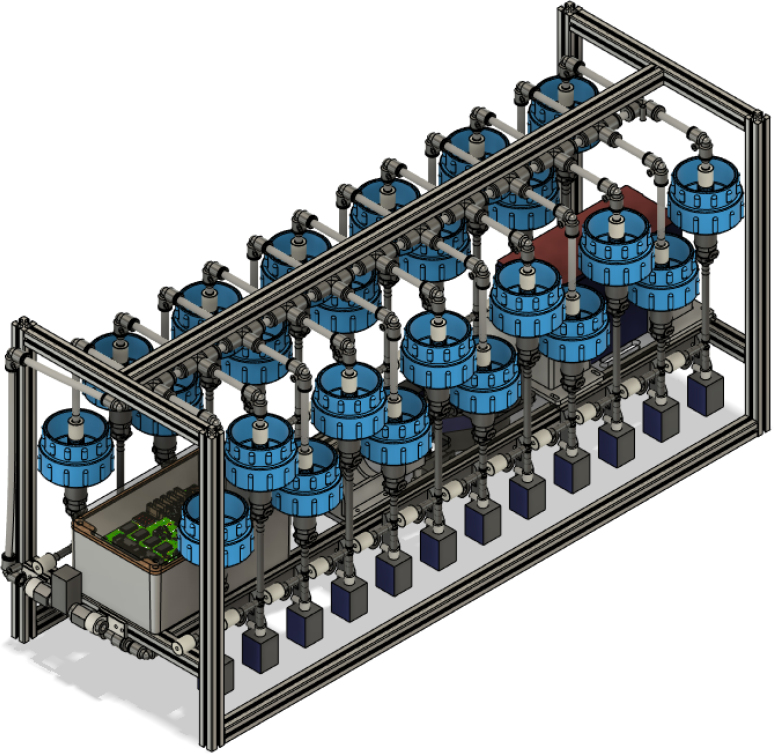
CAD image of the complete sampler assembly.

### 2.1 Hydraulics

The hydraulics of the sampler can be split into the following sections:

• The Pump and Inputs
• The Lower Hydraulics
• The Filter Housings
• The Upper Hydraulics and Outputs

#### 2.1.1 The pump and inputs

**Fig. 2:**
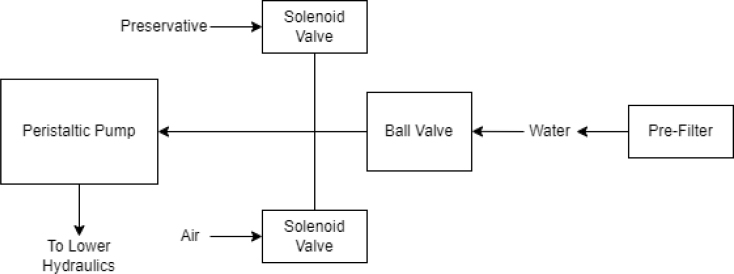
Pump and input hydraulics.

**Fig. 3:**
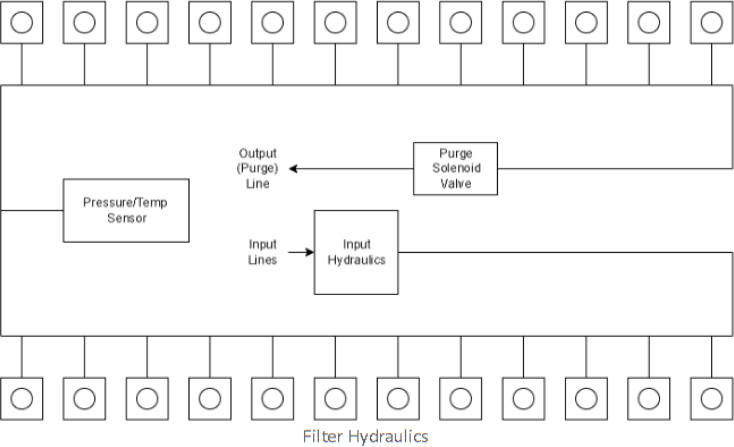
Lower hydraulic system.

**Fig. 4:**
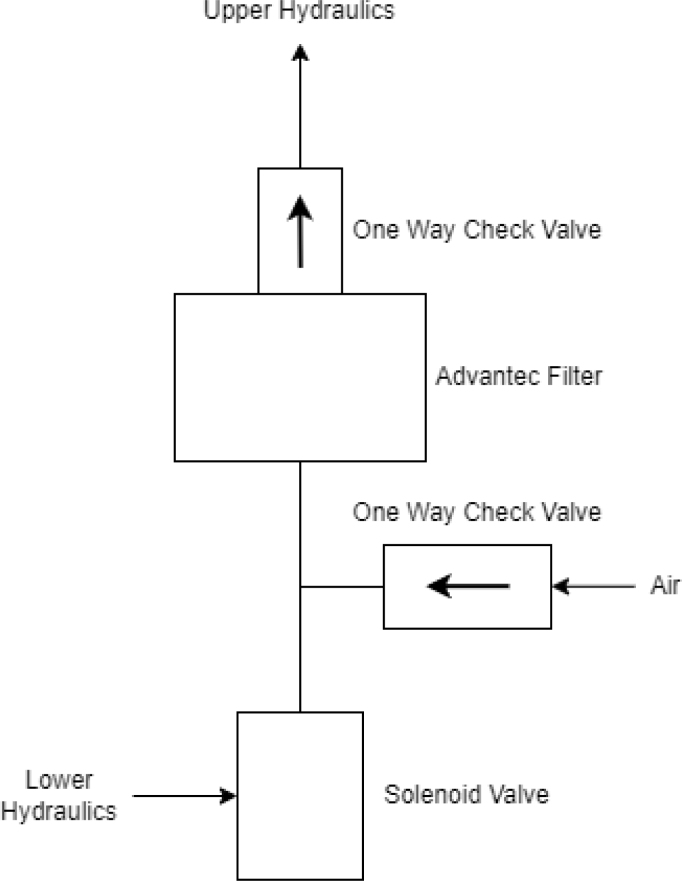
Filter hydraulics.

**Fig. 5:**
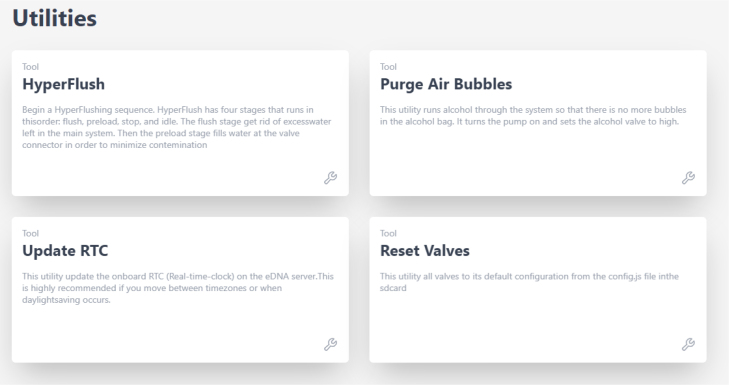
The utilities page in the PolyWAG sampler user interface.

**Fig. 6:**
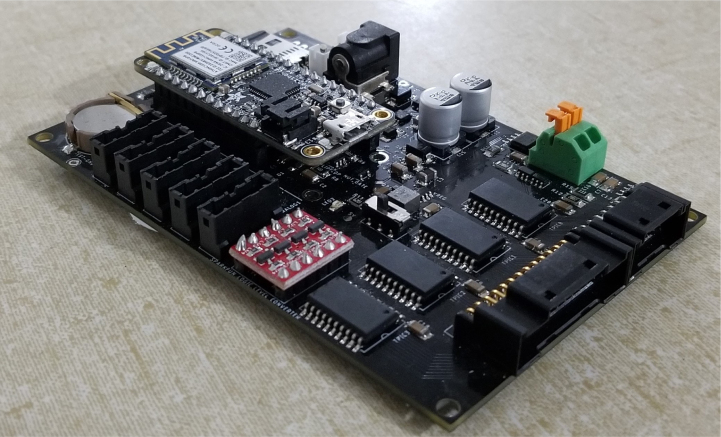
The main electronics control board for the PolyWAG sampler.

**Fig. 7:**
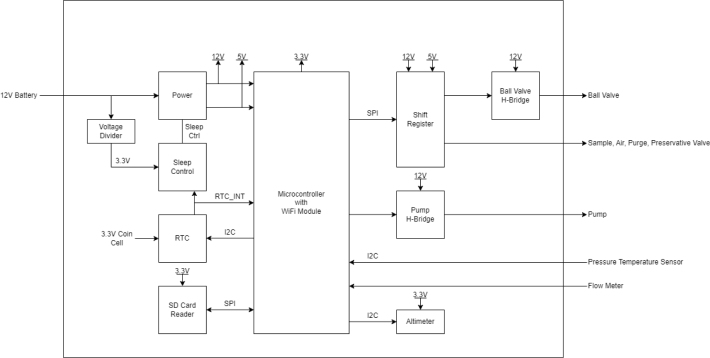
Block diagram of the main electronics control board.

**Fig. 8:**
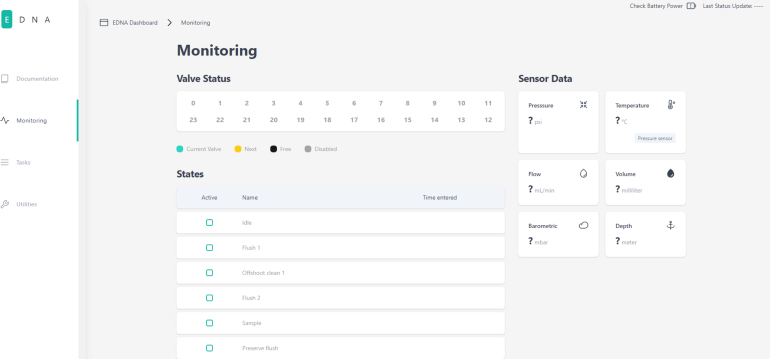
The monitoring page in the PolyWAG sampler user interface.

There are three inputs into the sampler: one for air, one for preservative, and one for water. The preservative input is connected to a hydration bladder where the preservative of choice can be stored. The sample water input has a prefilter at the front end of the tube to prevent debris from entering the sampler. Three valves are used to control the flow from these inputs with the air and preservative being regulated by solenoid valves and the water being controlled by a ball valve. These three valves connect into a single tube connected to the input of the peristaltic pump. The pump is capable of 400mL/min of flow under ideal conditions. The output of the pump connects directly into the Lower Hydraulic Rail (see Fig. 1, Fig. 2, Fig. 3, Fig. 4, Fig. 5, Fig. 6, Fig. 7, Fig. 8).

#### 2.1.2 The lower hydraulics

The Lower Hydraulic Rail consists of 24 solenoid valves connected parallel to each other and controls which filter liquid flows through. These filter valves are split into two sets, one on each side of the sampler. In between these two sets of valves is a M32JM-000105-100PG pressure and temperature sensor. The temperature is logged for later use and the pressure is used for monitoring, stopping an operation if the pressure exceeds a certain margin. At the end of the Lower Hydraulic Rail is another solenoid valve which allows for the lower hydraulics to be purged of their current contents when necessary.

#### 2.1.3 The filter housings

Downstream of each filter solenoid valve there is a tee connection that goes to a one-way check valve and a modified Advantec filter. The one-way check valve allows air into the solenoid valve that opens when the pump runs backwards. The Advantec filter is modified with a CPC quick disconnect and a one-way check valve. The one-way check valve is connected to the Upper Hydraulics and is used to prevent liquid from going backwards through the filter. The Upper Hydraulics simply connects the output of all the filters to one central line that goes through a flow meter and out of the sampler.

#### 2.1.4 The upper hydraulics and outputs

The output of the filters connect into a single output hydraulic line. This line is the main output of the filters, any water that goes through the filters will end up going though this line. This allows for a single flow meter to be added that can measure the flow going through any of the filters. This flow meter is crucial as this is how the sampler knows how much water has gone through a filter. After the flow meter the output line goes outside of the sampler and lets liquids flow back into the environment.

### 2.2 Sampling procedure

Having worked on multiple iterations of the sampler, we have decided to go with a 13-step sampling sequence that helps reduce cross contamination significantly. This sequence can be split into nine unique steps: Idle, Prefilter Clear, Flush, Offshoot Clean, De-pressure, Sample, Preservative Flush, Preservative, Air Flush, and End.

1. Idle
2. Prefilter Clear
3. Flush
4. Offshoot Clean
5. Flush
6. Sample
7. De-pressure
8. Preservative Flush
9. Preservative
10. Flush
11. Offshoot Clean
12. Air Flush
13. End

#### 2.2.1 The idle state

Idle is the default state of the sampler. The sampler waits for a signal from the RTC to move to the first/next state of the Sampling Sequence. If the sampler is not in sleep mode, this is when a client would interact with the UI to do a handful of tasks such as setting up a Sampling Schedule or using the other task utilities. If the sampler is in sleep mode, then only the RTC and supporting circuits are powered. This means there is no way to interact with the sampler without exiting sleep mode.

#### 2.2.2 The Prefilter Clear

Once the RTC sends the signal to start a sample procedure, the sampler enters the Prefilter Clear (PC) state. The purge and input ball valve are opened, and the pump is run in the backwards direction. This will allow for air to flow from the purge and out the input line. This is used to clear the prefilter of anything that might be clogging it, such as accumulated debris. This state runs for X seconds, before moving onto the next state.

#### 2.2.3 The Flush state

The Flush state prepares the lower hydraulics before the next state. The Flush state starts with the purge valve and the ball valving opening, then the motor starts to run in the forward direction. This fills the lower hydraulics with sample liquid and clears out/dilutes and liquid that remained from previous sample. The Flush state runs for the time specified when the Sampling Schedule is created. We recommend a Flush time of six minutes.

#### 2.2.4 The offshoot clean

The OC state closes the purge valve and opens the filter valve for the filter which is about to be used. The pump runs backwards for a few seconds. This clears anything that might be in the tube between the valve and the filter (what we refer to as the offshoot). The Flush state is run one more time before moving to the Sample state.

#### 2.2.5 The Sample state

In the Sample state, the system pushes the sample water through the filter. This is done by opening the filter solenoid valve and Ball Valve and running the pump in the forward direction. The system moves to the next state when the target Sample Volume is reached. This volume is measured by a Flow Meter on the filter output line. There is an additional condition that will end the Sample state, Sample Time. This time cutoff was added since the filter clogs, rapidly decreasing the flow rate during the sample process. To prevent the sample state running for too long, the time limit was implemented. Both conditions are set during task scheduling. Since the pressure greatly increases due to the clogged filter, the de-pressure state is used to reduce the pressure in the lower hydraulics to ensure that the valves can operate consistently.

#### 2.2.6 The Preservative Flush

The Preservative Flush state is nearly identical to the Flush state except the Preservative input valve is used instead of the ball valve. The lower hydraulics are saturated with preservative, preventing additional sample water that may have been stored in the lower hydraulics from going through the filter. If this water was allowed through the filter, then the Sample Volume would be inaccurate by the end of the sequence.

#### 2.2.7 The Preservative state

The Preservative state is like the Sample state except preservative is the input fluid instead of sample water. This state runs for a time specified by the user during scheduling.

#### 2.2.8 The Air Flush

Before the Air Flush, another sequence of Flush and Offshoot Clean states are run to purge the leftover preservative in the lower hydraulics. After these two states, an Air Flush (AF) state is run which is identical to the Flush and PF states but uses the air valve as the input instead of the other two inputs. This ensures that any liquid that is in the lower hydraulics is purged.

#### 2.2.9 The End state

In the End state, the system sets an RTC alarm for the time of the next sample. The system then moves into Idle and if the system was in sleep mode, then the system will go into its low power state.

### 2.3 Utilities

The HyperFlush utility runs water through every filter sequentially for a few seconds per filter. This is mainly used for cleaning out the system after a sample task (i.e., a set of 24 samples) to prevent any unwanted cross contamination. This utility can also be used to test the basic functionality of the sampler, as nearly every component is activated during this sequence.

The Preservative Air Purge (PAP) utility turns the pump on and opens the alcohol valve for 10 s. This runs some alcohol through the system and removes air bubbles from the alcohol bag. Often it helps to use this utility multiple times and to tilt the Preservative Bladder so that the air is near the port.

The Update RTC utility is needed to make sure that the time on the sampler matches your local time, so scheduling a task will remain accurate. Whenever the system is fully depowered (ie the battery is removed), or when new code is uploaded to the microcontroller, the RTC will need to be updated. It is also recommended that the RTC is updated when there is a daylight-saving change, or when you move between time zones.

The Reset Valves Utility is used when valves have been sampled that you want to be sampled again. This is required since the system ‘locks’ the filter valves when they have been used in a sample, this prevents samples from being corrupted accidentally. The code does not let you sample a valve multiple times without being reset to prevent messing up a sample. It is important to note that this utility will reset all valves, not a specific one.

### 2.4 Electronics

The PolyWAG Sampler is designed with a custom electronics control board that can be split into eight to ten blocks with an Adafruit Feather M0 at its core. These blocks consist of the microcontroller/Wifi Block, Power, RTC, and sleep control blocks, and the output blocks consisting of the Shift Register, Pump, and Ball-Valve H-Bridge Blocks.

The power block consists of a reverse polarity current (RPC) circuit and a voltage regulator circuit. The RPC Circuit was added to protect the 12 V battery from current flowing backwards through the system. While the battery has its own protection circuits, they lock the battery in the case of a short and need to be reset using the battery charger. The RPC circuit was added to prevent any “permanent” power loss while in the field. The voltage regulator circuit is a 12 V to 5 V regulator with an enable pin that connects to the sleep control circuit. This is used to save power during long term deployments.

The RTC and sleep control circuit are used to keep track of time and to save power respectively. The sleep control circuit controls the output of the power circuit and is constantly being power by a simple voltage divider circuit. It is basically a Flip Flop circuit that is reset when the RTC triggers an interrupt. The RTC circuit is used to keep track of the time between samples and is powered by a coin cell while power is off. This allows it to keep accurate track of time and signals an interrupt when its internal alarm is triggered. This interrupt is used to both turn power back on and to inform the microcontroller that it is time for a sample. If noise causes the sleep control circuit to reactivate power, the microcontroller will see that the RTC did not trigger the interrupt and will fall back into power saving mode.

The shift register circuit consists of four 8-bit shift registers connect to the microcontroller via SPI. The shift registers are pull-down style shift registers where the ‘output’ pins are pulled to ground. This allows the shift registers to control devices that use higher logic voltages. This allows us to control the 27 12 V solenoid valves with a 5 V IC. The shift registers are also used to control the H-bridge for the Ball valve. The H-bridge for the pump is controlled directly by the micro-controller itself.

The board contains an SD Card circuit for data logging purposes. The data is logged every second and includes the current state, time, and data from the sensors. The sensors include an in-line pressure temperature sensors for monitoring the lower hydraulic line and a flow meter out the output for measuring volume.

The micro-controller of choice is an Adafruit Feather M0 WiFi. The WiFi version of the Feather M0 was chosen as the user interface requires the feather to host a web-server.

### 2.5 User interface

PolyWAG Sampler hosts a webserver that can be connected to via a browser. This acts as the user interface for the system. There are three main sections that make up the user interface: monitoring, tasks, and utilities. The monitoring page displays the data from the sensors, the current state of the sampling procedure, and information on the sampling valves such as the current valve being sampled, and which valves are locked and unlocked.

The utilities page is used to activate the utilities mentioned earlier. The tasks page is where sampling tasks are created. Multiple tasks can be created, and each task is saved in memory for later modification and use. This page is also where tasks can be scheduled for sampling. Each task contains the information on which valves are being used as well as for how long each state occurs.

### 2.6 Final assembly and weather-proofing

The unit is protected from the weather in a variety of ways. First, the entire unit is placed inside a waterproof pelican case. Secondly, the electronics are contained in a splash proof electronics box with desiccant packets to prevent exposure to liquids or moisture. Finally, the electronics board is conformal coated before deployment in the field. Altogether, the unit, including pelican case, sampler, and 1 month of ethanol preservative, weighs about 43 kg (see Table 1).

**Table 1:** Design files fo eDNA sampler.

Design filename	File type	Open source license	Location of the file
CAD Assembly	CAD file	CERN-OHL-S 2.0	Available with the article
Battery Bracket	CAD file	CERN-OHL-S 2.0	Available with the article
Sample Valve Mount	CAD file	CERN-OHL-S 2.0	Available with the article
Preservative Valve Mount	CAD file	CERN-OHL-S 2.0	Available with the article
Flow Meter Mount	CAD file	CERN-OHL-S 2.0	Available with the article
Tube Guide	CAD file	CERN-OHL-S 2.0	Available with the article
Central Assembly Mount	CAD file	CERN-OHL-S 2.0	Available with the article
Control Board Mount	CAD file	CERN-OHL-S 2.0	Available with the article
Electronics Box Lid	CAD file	CERN-OHL-S 2.0	Available with the article
Control Board Schematic	EDA file	CERN-OHL-S 2.0	Available with the article
Control Board PCB	EDA file	CERN-OHL-S 2.0	Available with the article
Switch Breakout Schematic	EDA file	CERN-OHL-S 2.0	Available with the article
Switch Breakout PCB	EDA file	CERN-OHL-S 2.0	Available with the article
UI Code	Software	GPL 3.0	Available with the article
Device (Server) Code	Software	GPL 3.0	Available with the article

## 3 Design files summary

• The CAD Assembly is a CAD file with every major components. The tubing and minor things such as zip ties for cable routing are not included.
• The Battery Bracket is a 3D-Printed component to hold down the battery during transit. Paired with a Velcro strap, the battery does not move.
• The Sample Valve Mount is a 3D-printed bracket that holds four solenoid valves. There are six brackets in the sampler and each valve corresponds with a filter.
• The Preservative Valve Mount is a 3D-printed component that holds the preservative valve in place.
• The Flow Meter Mount is a 3D-printed bracket that holds the flow sensor to the frame.
• The Tube Guide is a 3D-printed components that helps hold the input and output tubes in place.
• The Central Assembly Mount is a laser-cut acrylic components that all of the “central” components mount to. This includes the pump, input control valves, and the battery.
• The Central Board mount is a laser-cut acrylic components that the allows the main control board to mount inside the electronics box.
• The electronics box lid is a CNCed Acrylic components that replaces the metal lid but maintains the groove for the gasket.
• The Control Board Schematic and PCB are EDA files in the Autodesk EAGLE format for the sampler’s main control board.
• The Switch Breakout Schematic and PCB are EDA files in the Autodesk EAGLE format for the sampler’s main power switch.
• The UI Code is the codebase for the web application. The built UI files are stored in the sampler’s SD Card.
• The device (server) code is the codebase for the sampler that is uploaded to the microcontroller. It handles all of the sampler’s functions.

## 4 Bill of materials

Given the number of materials required to build a PolyWAG Sampler, The BOM is located in the zenodo repository and can be found here

## 5 Build instructions

This section details the fabrication of PolyWAG eDNA sampling system. The sampler requires two main skill sets to produce a unit: PCB assembly, and hardware manufacturing. The hardware assembly requires roughly 40 h of work for one person. Optionally, a PCB can be manually assembled, rather than purchased pre-assembled, as outlined in the electronics board section following. The PCB assembly requires roughly two hours of assembly time and an additional 24–72 h of wait time if applying and curing a conformal coating.

### 5.1 Filters

**Fig. 9:**
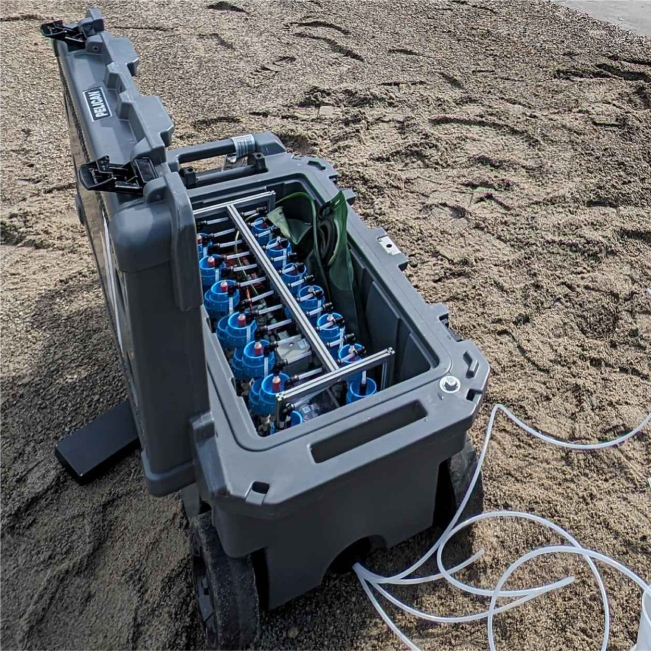
The sampler deployed in a pelican case for weather protection.

This section covers the assembly of the Filter housings, which hold the 47 mm diameter filter paper. These filters housing are made by modifying an Advantec filter housing with a quick disconnect and a one-way check valve into two sizes. The quick disconnect makes it easier to remove the valve from the sampler and the one-way check valve prevents water from going backward through the filters, which is needed because all filter housings share the same output line in the sampler. A fully assembled filter can be found in Fig. 9.

**Fig. 10:**
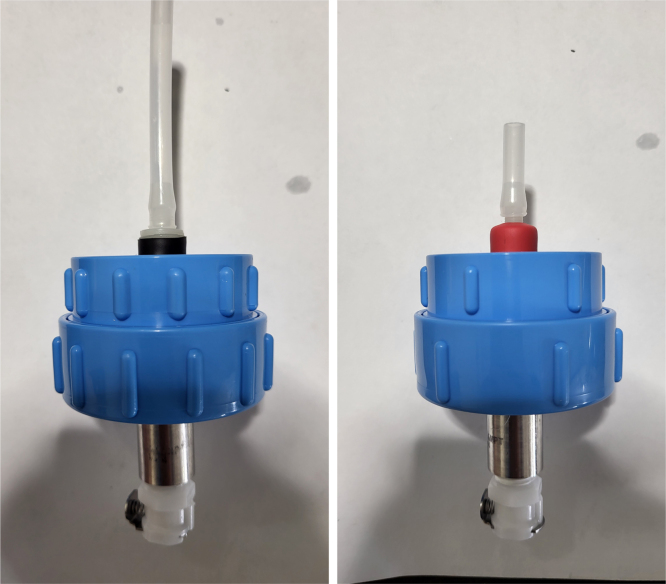
Fully assembled filter housings.

**Fig. 11:**
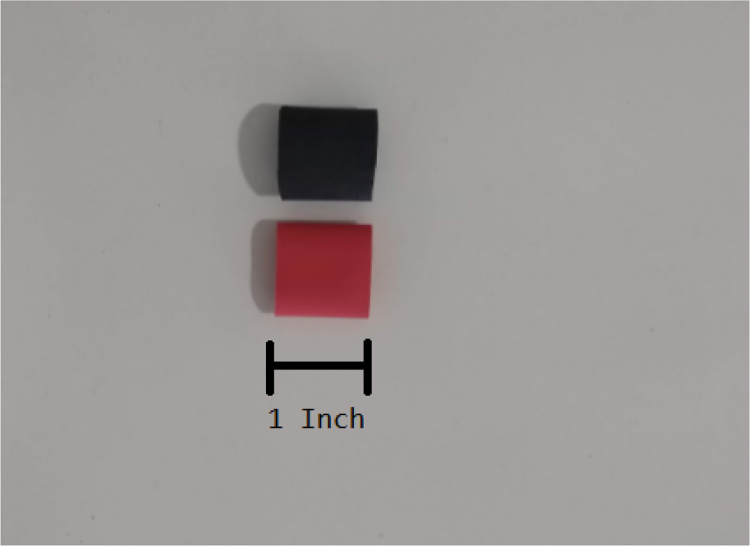
Heat shrink cut into 1 inch length pieces.

#### 5.1.1 Cut heat shrink and tubing

1. Cut 1-inch sections of the heat shrink tubing — 12 red and 12 black.
2. Cut the thin-walled tubing with the following amounts and dimensions using a standard tube cutter: 12 tubes at 28 mm, 12 tubes at 77 mm (see Fig. 10, Fig. 11, Fig. 12, Fig. 13, Fig. 14, Fig. 15, Fig. 16, Fig. 17, Fig. 18, Fig. 19, Fig. 20, Fig. 21, Fig. 22, Fig. 23, Fig. 24, Fig. 25, Fig. 26, Fig. 27).

**Fig. 12:**
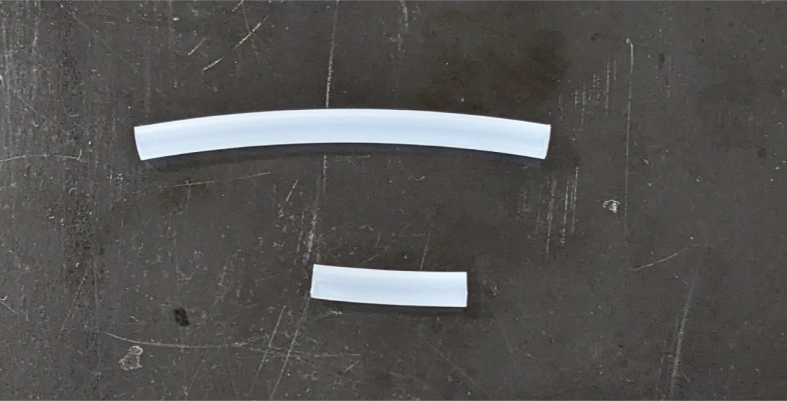
28 mm and 77 mm length thin-wall tubing.

**Fig. 13:**
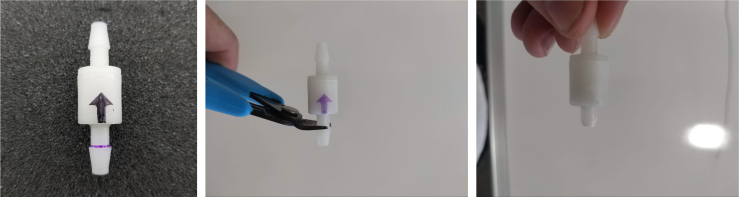
Process for cutting the barbed tip of the spring check valve.

#### 5.1.2 Modify the spring check valves

1. Cut the barbed tip of the inlet side of the check valve (see the purple line in the left image). Push the back of the flush cutters against the edge of the purple line.
  Cut the check valve gently and **not all at once**. Work at it slowly by twisting the valve back and forth while applying pressure with the flush cutters.
2. Thread the valve using the side of the **1/4” NC20** die with the larger hole.
  Put the tip of the valve into the 1/4” die and twist the valve clockwise into the die.
3. Flip the 1/4” **die** over and thread the valve again from the other side **with the smaller hole.**
4. Repeat steps 1–3 until you have 24 modified check valves as shown below:

**Fig. 14:**
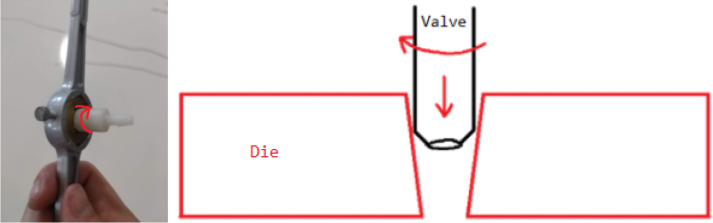
Process for threading the spring check valve.

**Fig. 15:**
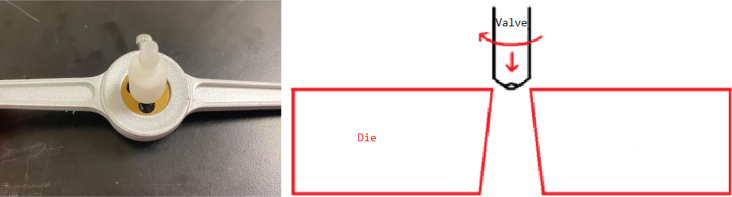
Process for threading the opposite side of the spring check valve.

**Fig. 16:**
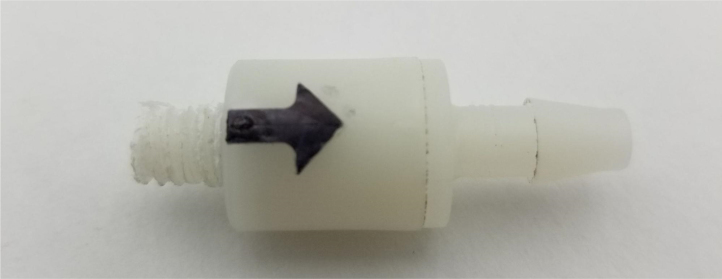
A completed modified spring check valve.

**Fig. 17:**
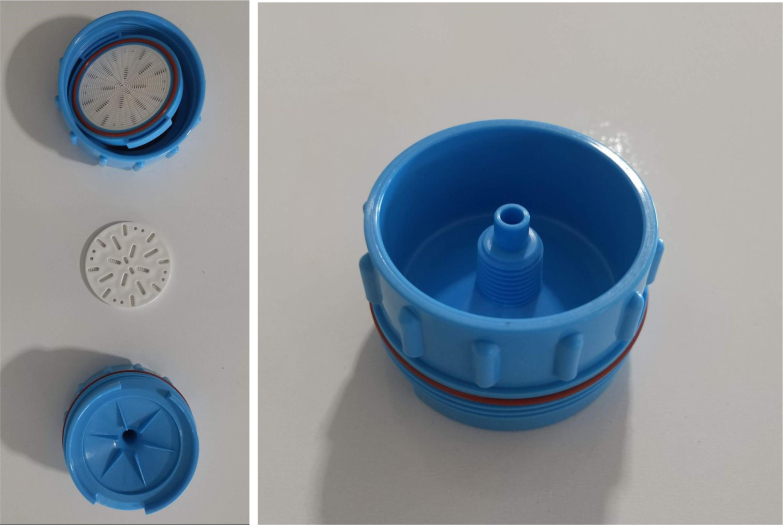
Disassembled Advantec filter housing.

**Fig. 18:**
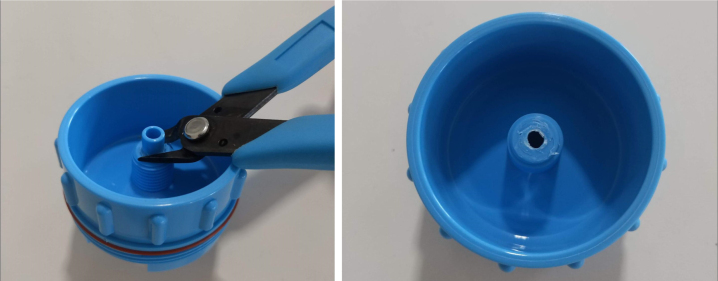
Removing filter housing extruded tube.

**Fig. 19:**
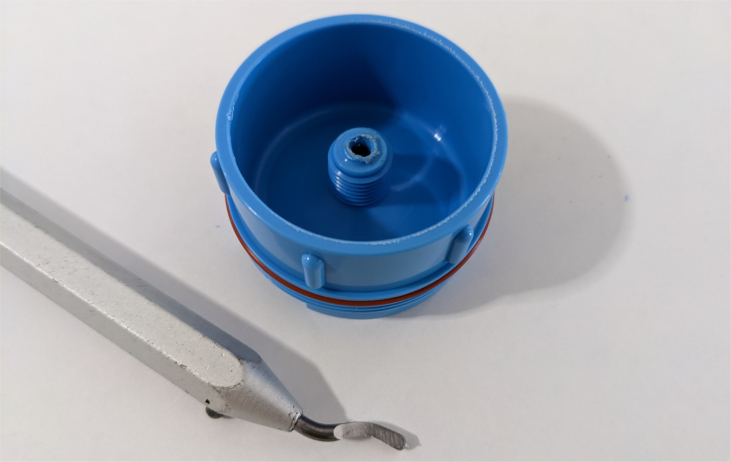
Deburring the modified filter housing.

#### 5.1.3 Modify the advantec filter

1. Take apart the filter and take out the inner filter holders. The larger piece of the housing is the one that you will be working with.
2. Use the flush cutter to cut the extruding tube off (the thin and unthreaded one).
  Rotate in a circle while you gently squeeze on the cutters in order for the filter not to crack.
**Note: You may need to use a deburring tool to clear off extra plastic bits:**
3. Slowly twist the **1/4” NC20** male tap into the now open hole while applying light pressure downward to get the tap to bite into the plastic:
  • **You may need to use a #10 drill to enlarge the hole before tapping.**
  • **When removing the tap, do not apply force vertically, let the threading guide the tap out.**
  • Blow the waste material off of the filter housing.
4. Screw the filter back together and make sure that the pieces all align:

#### 5.1.4 Assemble the filter housing

1. Apply 3–4 layers of Teflon tape onto the CPC Socket and the threaded Advantec filter housing.
  **Note:** When applying Teflon tape, apply it in the same direction as the thread.
2. Thread the CPC Soecket into the 316 SS 1/4” NPT Coupling until it is hand-tight.
  Use gloves (any kind will work) when working with machined metal parts.
3. Attach the coupling to the filter housing.
  Use a wrench to tighten everything fully onto the filter housing.
4. Apply Teflon tape to the threaded check valve and attach the valve to the interior thread of the filter housing until it snugs up against the filter housing and begins to resist turning slightly. **Do not over-tighten it — this can lead to the check valve thread breaking off.**
  **Note:** Keep in mind the directional nature of applying Teflon tape.
5. Put the 1” sections of heat shrink around where the check valve and housing meet. Make sure that the base of the heat shrink touches the bottom of the filter housing.
  Use 12 black and 12 red pieces in total; this is important later on.
  Use a Heat-gun or Reflow station set to 140 ℃ to fully shrink the heat-shrink.
6. Attach the **77 mm** length tube to the filters with the **black** heat shrink.
  Attach the **24 mm** length tube to the filters with the **red** heat shrink.
  Use a Heat-gun or Reflow Station set to 135 ℃ and heat up the edge of the tubing.
  When you start to notice the edges of the tubing start to melt, immediately push the tubing over the barbed end of the check valve. **Do not let the tubing melt too much, or it will not make a good seal.**

**Fig. 20:**
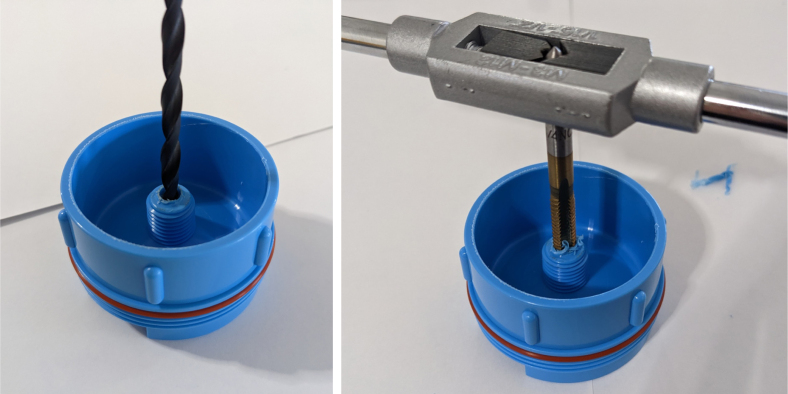
Tapping the modified filter housing.

**Fig. 21:**
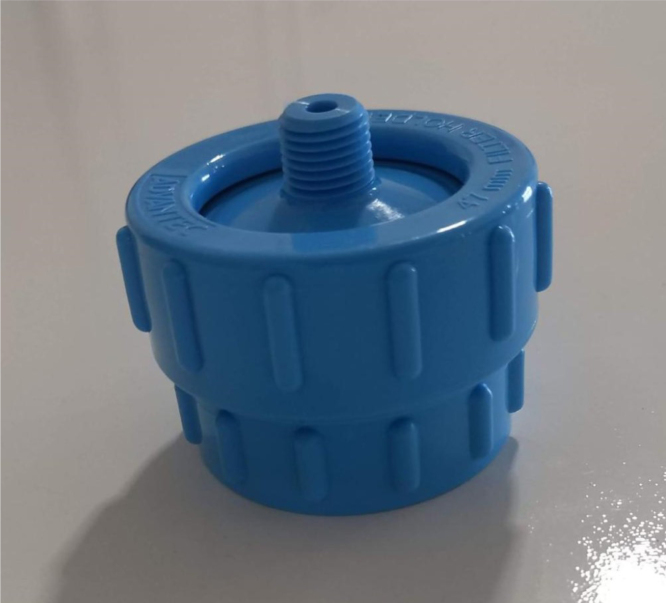
Reassembled modified filter housing.

**Fig. 22:**
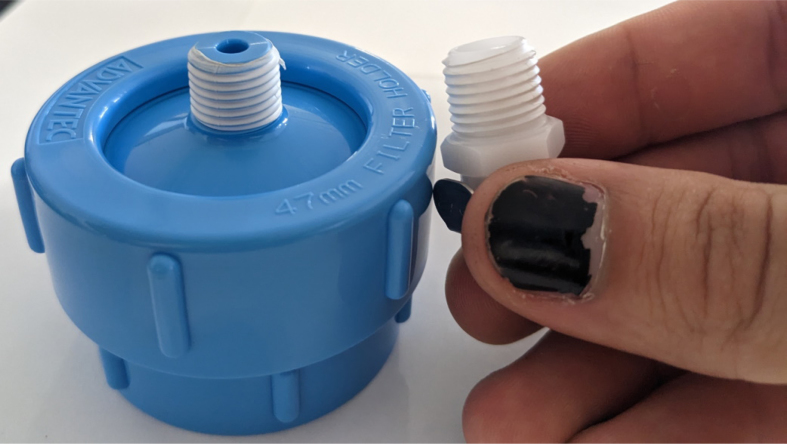
Threading with Teflon tape applied.

**Fig. 23:**
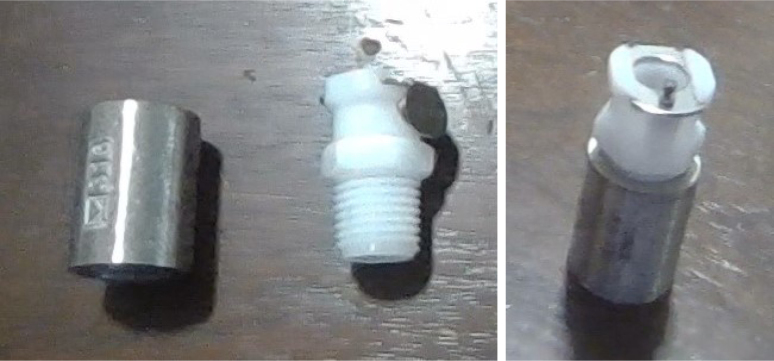
A completed filter housing coupling.

**Fig. 24:**
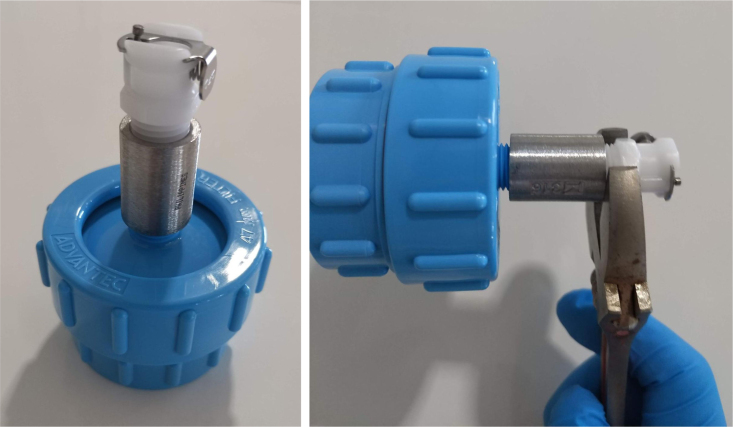
A modified filter housing with coupling.

**Fig. 25:**
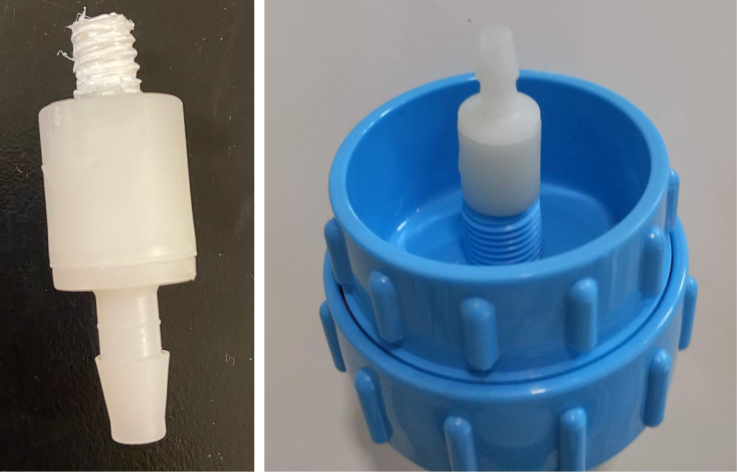
A modified filter housing with check valve.

**Fig. 26:**
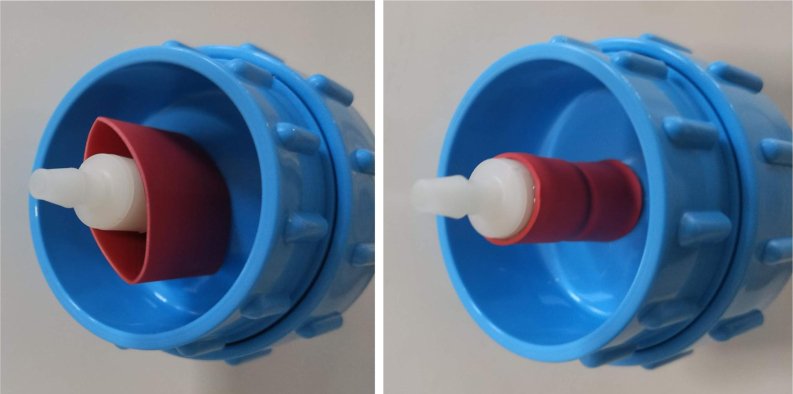
The modified filter housing with heat shrink applied to check valve.

**Fig. 27:**
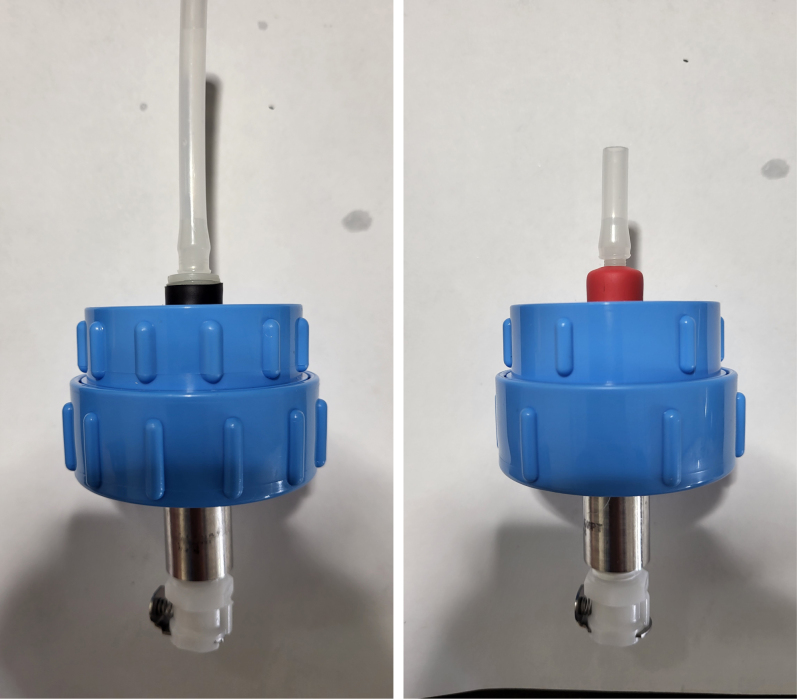
Fully assembled filter housings.

### 5.2 Electronics board

**Fig. 28:**
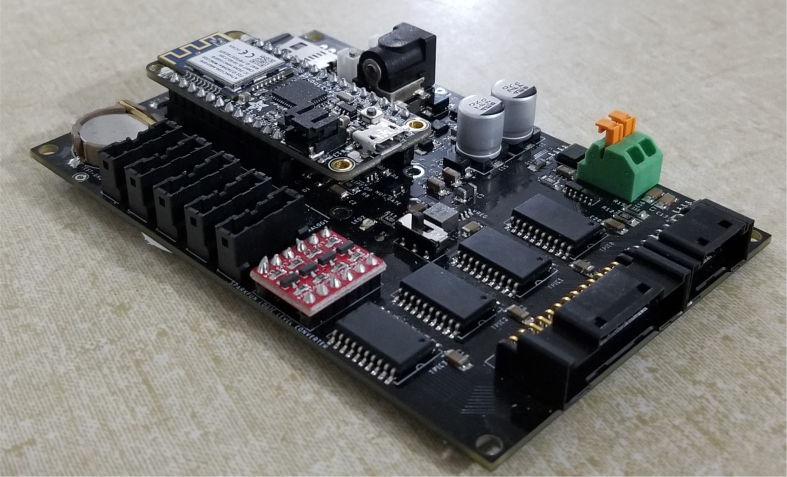
Fully assembled eDNA electronics board.

**Fig. 29:**
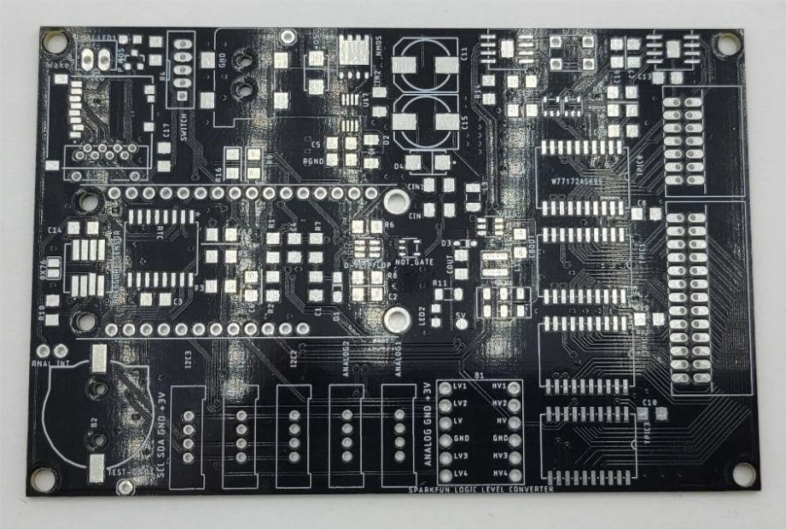
Bare PCB without SMD components.

**Fig. 30:**
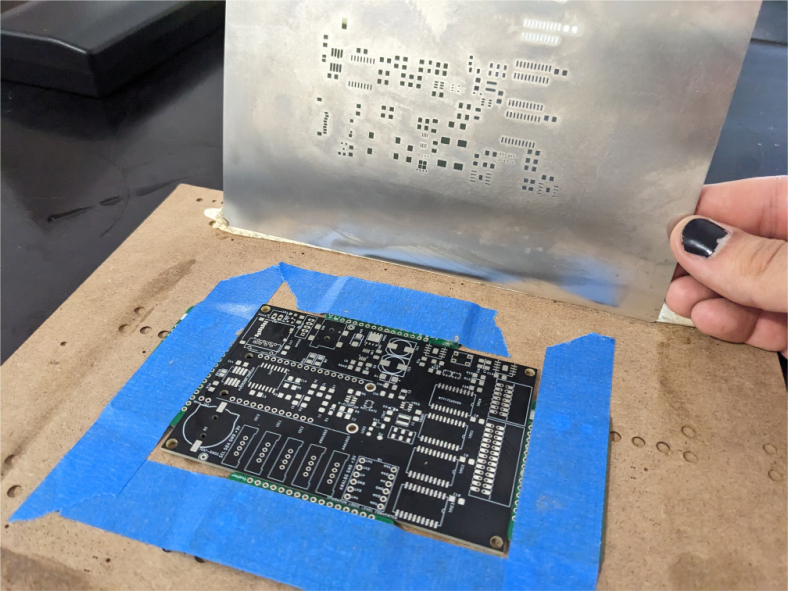
Solder Stencil Aligned over PCB.

**Fig. 31:**
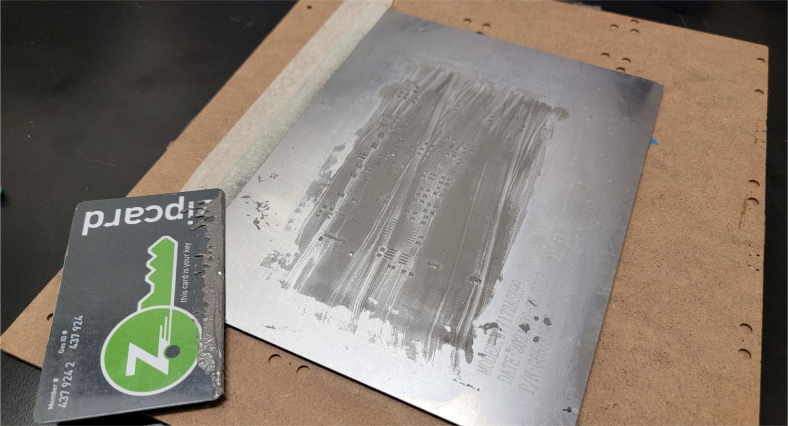
Solder Stencil with applied solder paste.

**Fig. 32:**
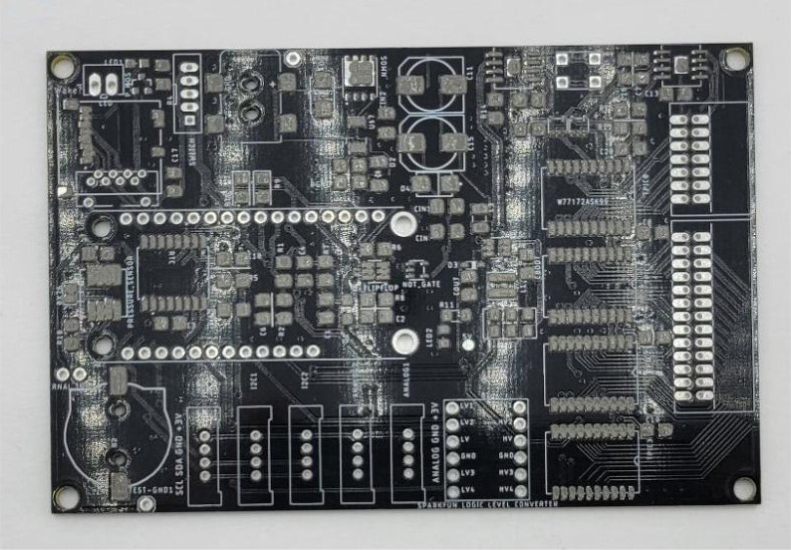
Bare PCB with applied solder paste.

**Fig. 33:**
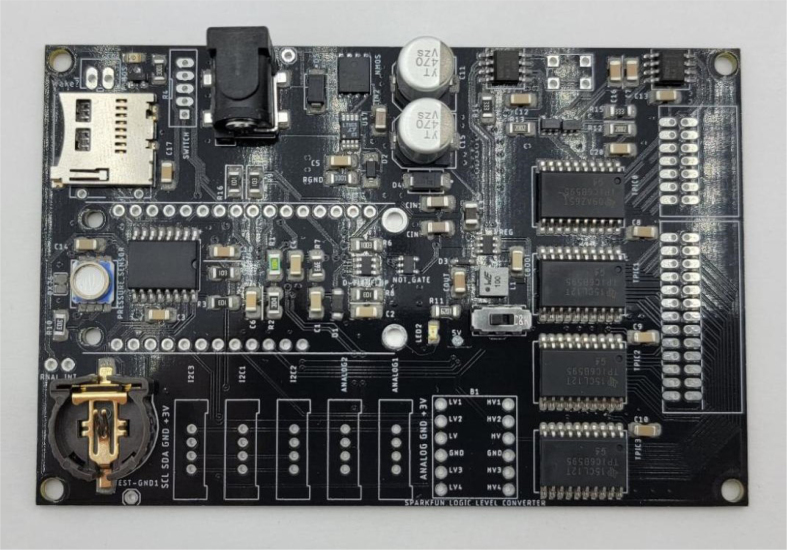
Filled PCB with SMD components applied to solder paste on board.

**Fig. 34:**
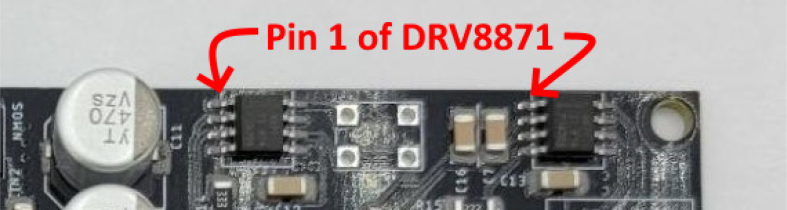
DV887 Pin 1 location with missing orientation designation.

**Fig. 35:**
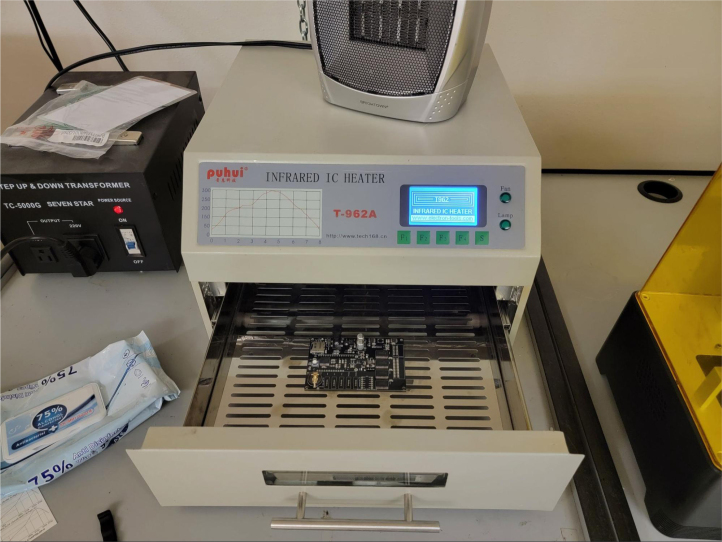
SMD oven with set components.

**Fig. 36:**
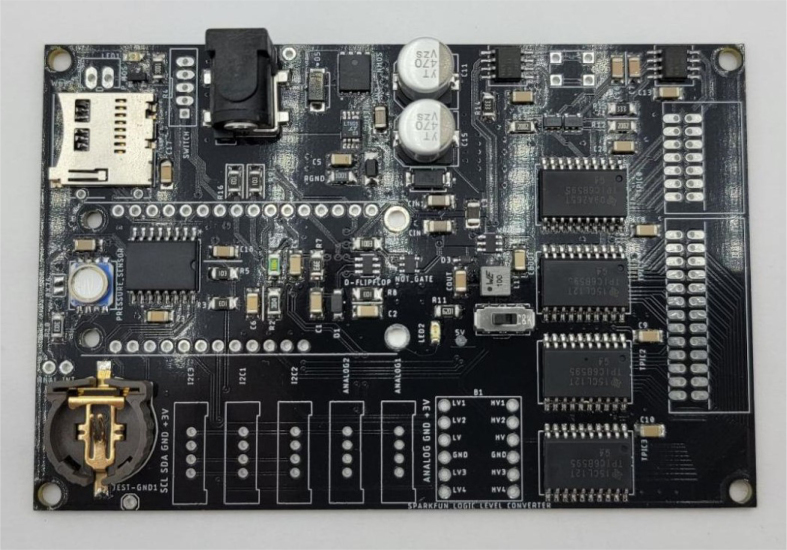
Baked PCB with assembled SMD components.

**Fig. 37:**
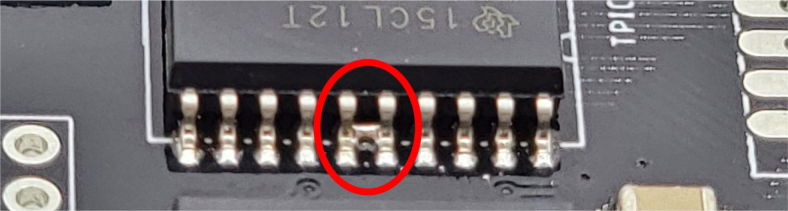
IC component displaying solder bridging.

**Fig. 38:**
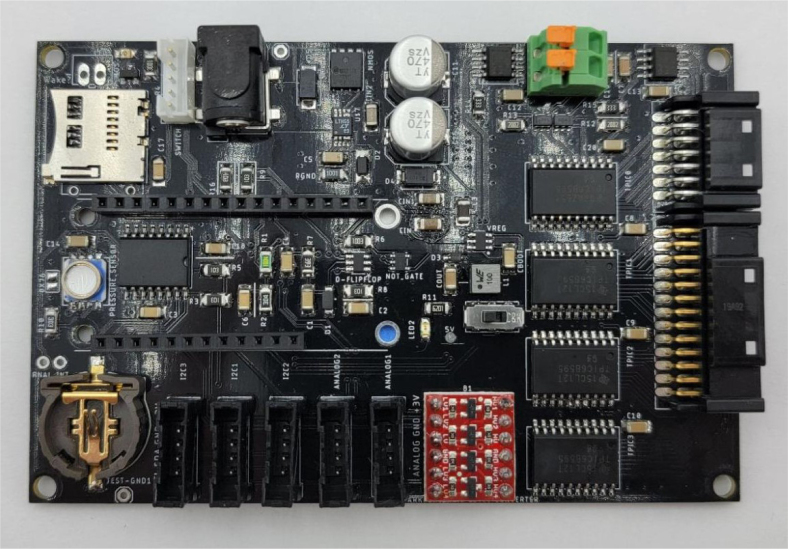
Fully assembled PCB with SMD and through-hole components.

**Fig. 39:**
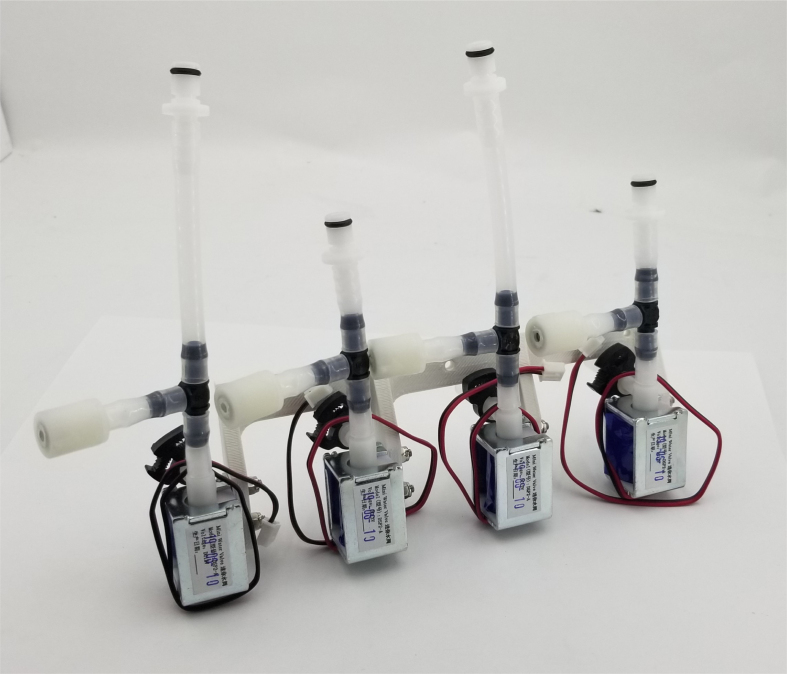
A fully assembled valve hanger.

**Fig. 40:**
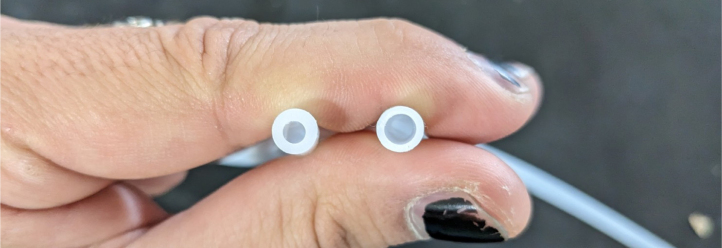
A comparison of thick-walled vs thin-walled tubing.

**Fig. 41:**
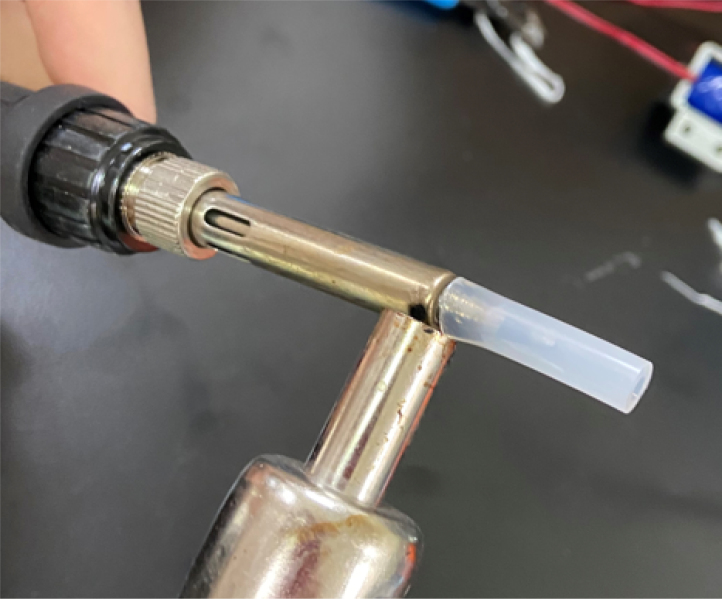
Thick-walled tubing being heated until flexible.

**Fig. 42:**
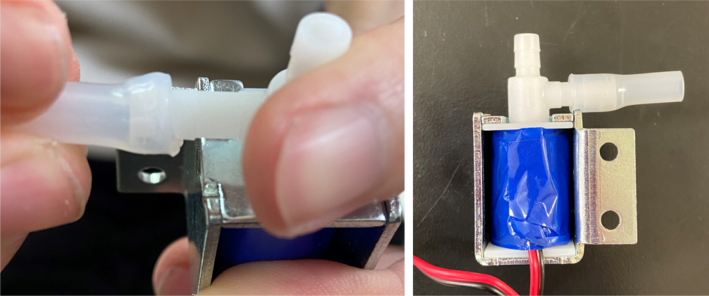
Thick-walled tubing connected to solenoid valve.

**Fig. 43:**
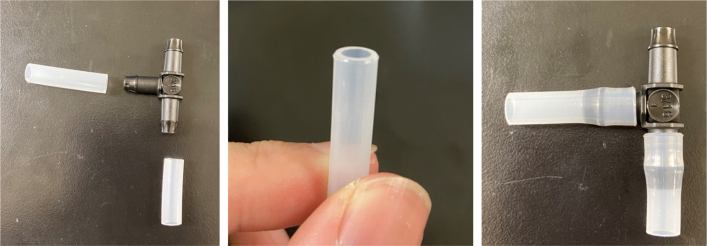
The barbed tee with attached thin-walled tubing.

**Fig. 44:**
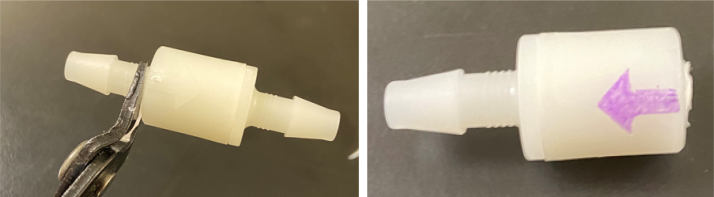
A cut one-way check valve with correct orientation.

**Fig. 45:**
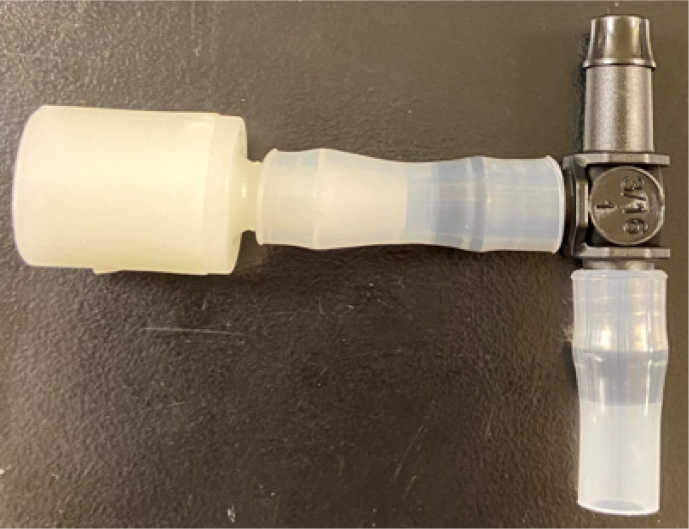
The Cut check valve attached to the barbed tee.

**Fig. 46:**
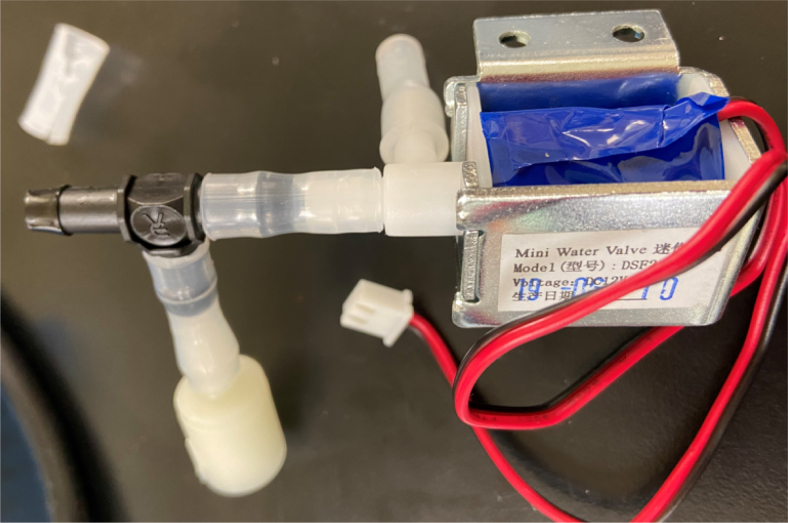
Barbed tee assembly connected to solenoid valve.

This section covers the assembly of the main electronics control board, a custom board designed by the OPEnS Lab, for the eDNA Sampler (Fig. 28). This board is the brain of the sampler and it is very important that it is assembled correctly. While custom boards can be bought pre-assembled, the OPEnS Lab, at the creation of this document, does not purchase the boards pre-assembled. This guide serves to explain how to assemble electronic boards and points out the important things to keep note of. If desired, the eCAD files in our zenodo repository contain everything needed to fabricate the edna electronics board with an assembly house of choice, such as PCBway (see Fig. 29, Fig. 30, Fig. 31, Fig. 32, Fig. 33, Fig. 34, Fig. 35, Fig. 36, Fig. 37, Fig. 38, Fig. 39, Fig. 40, Fig. 41, Fig. 42, Fig. 43, Fig. 44, Fig. 45, Fig. 46).

#### 5.2.1 SMD work

1. Get a bare PCB
2. Align the solder stencil with the PCB so that all of the pads on the board line up with the holes on the stencil. It is helpful to create a jig so that once the PCB is placed in the jig, the stencil/PCB will not move out of alignment.
3. Dispense a line of solder paste onto the stencil at one end of the board, and use a stiff but slightly flexible card to spread the paste across the stencil, forcing the paste to fill all the holes. Repeat if necessary. More solder paste may be needed to ensure that all of the holes are filled.
4. Carefully separate the stencil and the PCB, being very careful not to touch the surface of the PCB which now has solder paste on every pad.
5. Use Ethanol or Isopropyl Solutions and an abrasive sponge or towel to clean the stencil, making sure to remove as much solder paste as possible to ensure the stencil can be used again in the future.
6. Place the SMD components using the reference designators to determine which components go where. If you did not order the parts with reference designators already printed onto the parts bags we recommend using the EAGLE files and a Sharpie to add the reference designators for each part bag.
  • **NOTE 1:** Many parts have a specific orientation that they need to be placed in. Only resistors, inductors, and some capacitors can be placed in either direction. There are markings on the board itself as well as in the EAGLE Files.
  • **NOTE 2:** The bare PCBs we ordered are missing the orientation marking dot for the DRV8871 chips by the edge of the PCB. If this should happen, note that they should be placed as shown in the picture, with pin 1 towards the top left side of the board.
7. Once all of the SMD Components have been placed, insert the PCB in the SMD Oven tray (taking care not to dislodge any components).
8. Set the correct heating pattern (the specific heating pattern to use is determined by the solder paste used: many solder pastes come with a graph of the heating pattern). Set the SMD oven to this heating pattern and start the baking process.
9. Once the baking is finished and the PCB has had time to cool down, inspect the solder joints under a microscope.
  • Check the quality of the joints. They should be shiny and solid, not dull. If solder joints are poor, you can rebake the PCB at a higher temperature.
  • Check for solder bridges (solder bridging two pins where it should not). These can be fixed with desoldering braid and a soldering iron.
  • Check that all joints are solid by running a pair of tweezers lightly over the pins of ICs and make sure they do not move when brushed. These can sometimes be repaired by re-baking, or they can be spot-repaired with a fine-tip soldering iron under magnification.
10. Once any quality issues have been dealt with, solder all of the through-hole components to the PCB:
  • Hirose connectors
  • Feather M0 headers
  • Logic level converter
  • Switch connector
  • Motor terminal

#### 5.2.2 Conformal coating

A conformal coating can be applied to a PCB in order to prevent humidity and moisture from affecting the PCB in field conditions. To do so, coat the PCB using a conformal coating in a ventilated area. Make sure to avoid applying the conformal coating to any connectors on the PCB. It is recommended that all connectors are tapped off prior to applying the coating in order to prevent permanently damaging the connectors. After applying allow the conformal coating to set and cure over 24 to 72 h, depending on type used.

### 5.3 Valve hangers

This section covers the assembly of what is referred to as “Valve Hangers” internally. These are custom brackets designed to hold four solenoid valves which are used for controlling which filter is being sampled. The sampler has two mirrored sets of three valve hangers, six valve hangers in total for the eDNA Sampler.

**Fig. 47:**
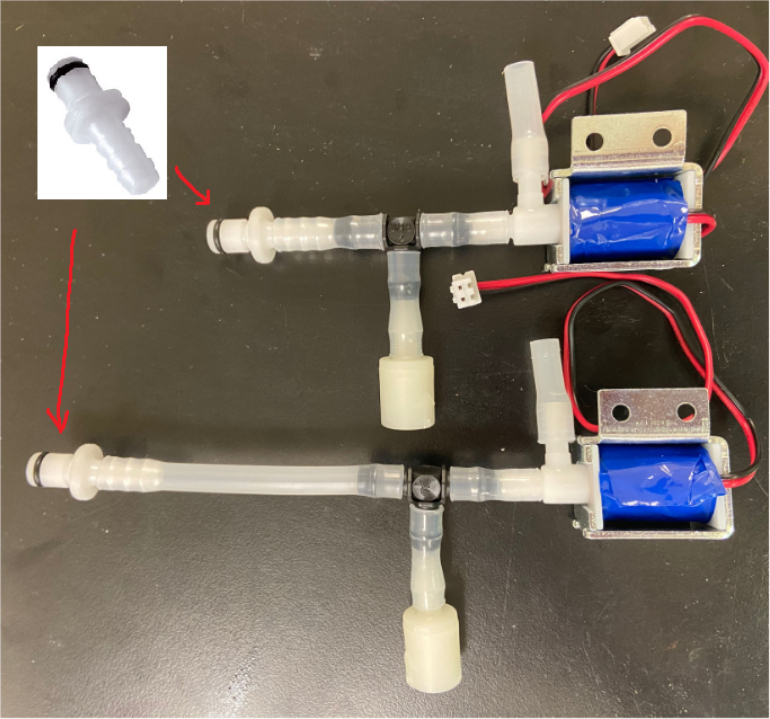
A complete solenoid assembly in both short and long configurations.

**Fig. 48:**
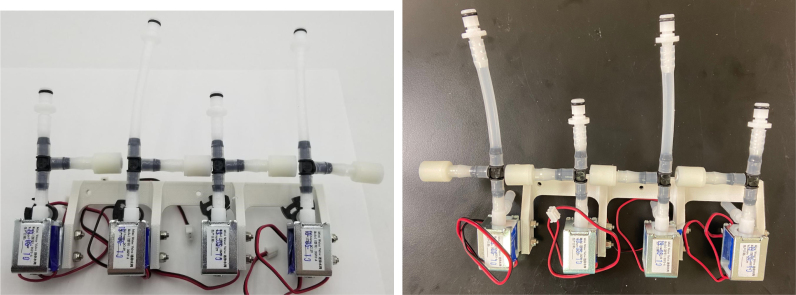
Assembled Valve hangers with mirrored configurations.

**Fig. 49:**
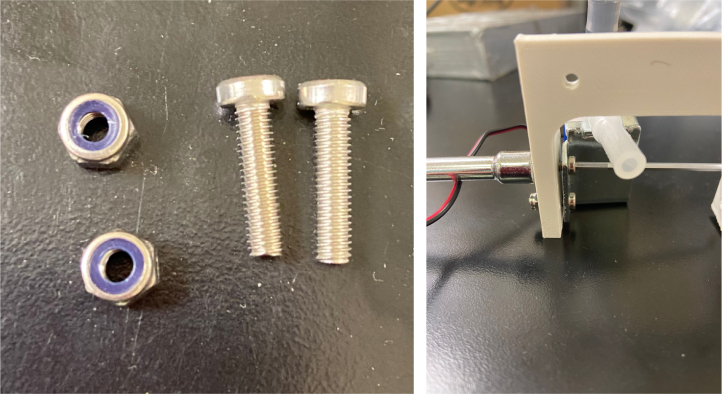
M3 × 12 mm bolts and M3 Nylock Nuts mounted in correct position.

#### 5.3.1 Assembling the individual valves

1. Cut 24 sections of **Thick**-Walled Tubing with a length of 26 mm.
2. Preheat the soldering iron to 100 ℃. Make sure that the soldering tip can fit inside the thick-walled tubing.
3. Set the heat Gun to 135 ℃.
4. Put the soldering iron inside the tube and heat the outside of the tub with the heat gun until the tubing begins to get clearer.
5. Once the tubing is flexible, put the tubing onto the side of the solenoid valve.
6. With the **thin**-walled tubing, cut 24 sections that measure 26 mm in length and 24 sections that measure 22 mm in length.
7. Use the heat gun to heat one of each size thin-walled tube and attach them to the $\frac{3}{16}$ Barbed Tee, heat and attach the tubes one at a time. The 26 mm tube attaches to the perpendicular barb while the 22 mm attaches to one of the parallel barbs.
8. Cut the “top” side of the One-Way check valves, the side that the arrow is pointing **away** from.
9. Heat the exposed end of the 26 mm tube, the one connected to the perpendicular barb of the tee, and attach the check valve.
10. Heat the exposed end of the 22 mm tube and attach the whole assembly to the solenoid valve.
11. With the **thin**-walled tubing, cut 12 sections that measure 74 mm in length and 12 sections that measure 28 mm in length.
12. Use the heat gun to attach these tubes to the last barb of the tees.
13. Heat the other end of these tubes and attach the CPC Sockets to the end of the tube.

#### 5.3.2 Assembling the valve hangers

There are a total of 6 valve hangers that go on the sampler, 3 for each side of the sampler. The Valves alternate between the ones with the long tubing and the ones with the short tubing.

**Note: The hangers that go on each side of the sampler are “mirrored” - see**Fig. 47**. While the 3D printed parts are identical, the side that the valves attach to and the pattern of long/short is mirrored** (see Fig. 48).

1. Use M3 × 12 mm bolts and M3 Nylock Nuts to attach the valves to the 3D-Printed Valves Hangers.
  Use a Hex Socket Screwdriver and a Hex Head Screwdriver to tighten the Nylock nut onto the bolt.
**NOTE: Do not over-tighten the nut as you could crack the 3D-Print. Tighten the bolt just enough to prevent the valve from moving and the bolt from spinning in place.**
2. Repeat the previous step for each valve being sure to alternate between long and short tubes and ensuring that 3 of the valve hangers start with the long tube, and 3 start with the short tube.

### 5.4 Frame

**Fig. 50:**
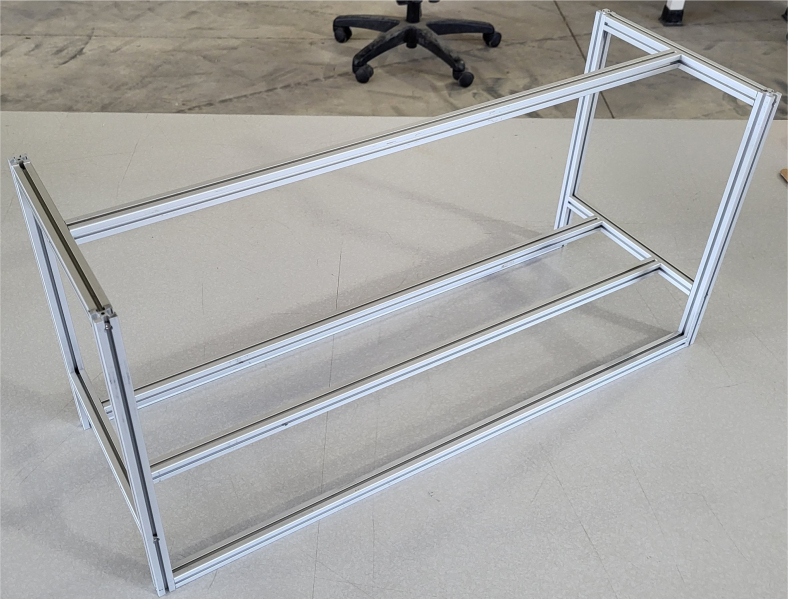
The fully assembled sampler frame.

This section covers the assembly of the eDNA Sampler’s frame, the physical backbone of the sampler. The frame is made with 15 mm x 15 mm aluminum extrusions that are modified to avoid using right-angled brackets, as shown in Fig. 49 (see Fig. 50).

#### 5.4.1 Tapping (hand tapping recommended)

The 610 mm, 200 mm, and 80 mm extrusions need to be threaded with the M3 tap at the ends of the extrusion.

**Fig. 51:**
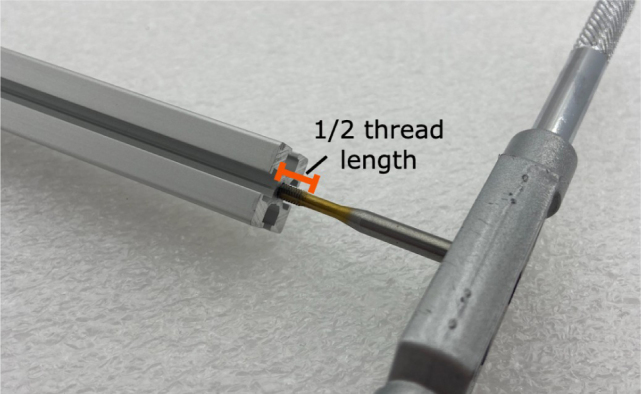
Tapping the extrusion pieces with the M3 tap at half thread length.

**Fig. 52:**
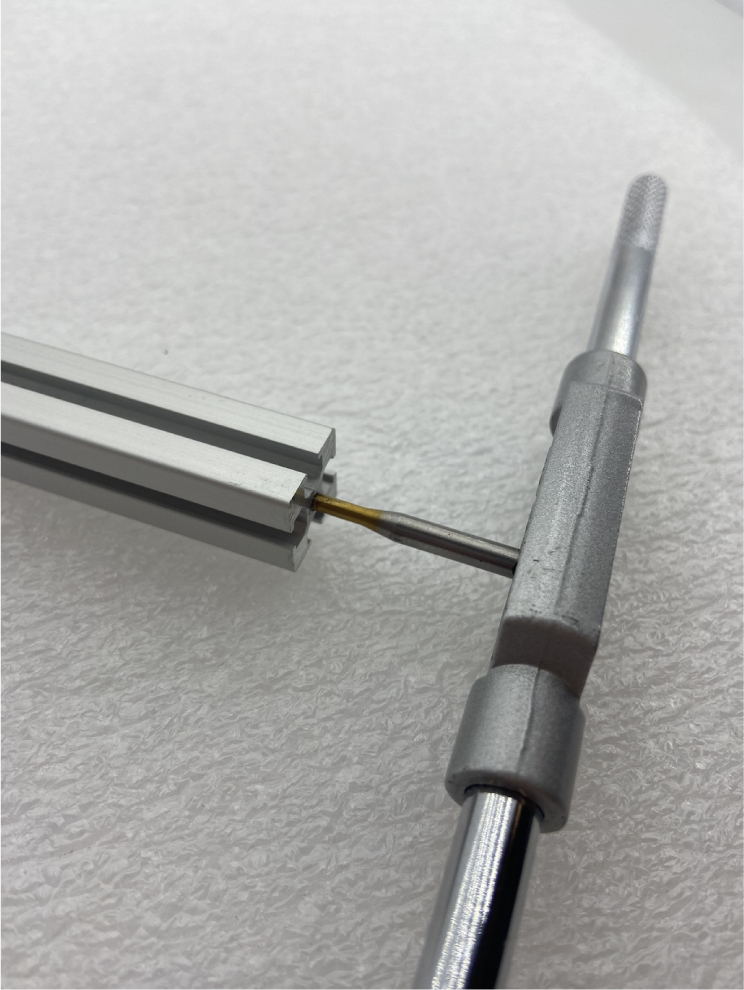
Tapping the extrusion pieces with the M3 tap at entire thread length.

**Fig. 53:**
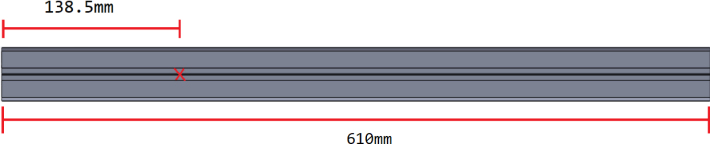
Tap hole locations for two of five 610 mm extrusions.

**Fig. 54:**
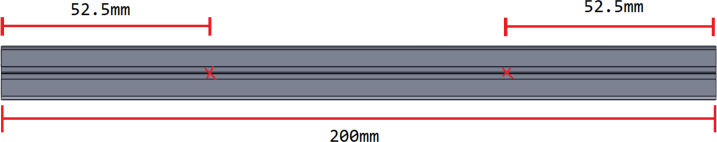
Tap hole locations for two of four 200 mm extrusion.

**Fig. 55:**
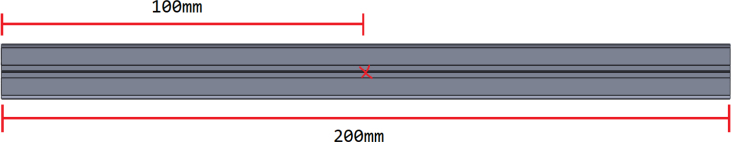
Tap hole locations for final two 200 mm extrusion.

1. Insert an extrusion into the vice.
2. Apply cutting fluid to the tap.
3. **Slowly** tap the extrusion, making sure the tap and extrusion are **parallel** with each other.
  For the first pass, thread **halfway** up the threading before backing the tap out.
  **Note:** If there is too much resistance at any point while tapping, back the tap out, discard excess aluminum, reapply tapping fluid, realign, and continue.
4. Back the tap out, discard the aluminum shavings and reapply the cutting fluid. Repeat step 3. This time, tap the **entire** length of the threading into the extrusion.
5. Repeat until the 610 mm, 200 mm, and 80 mm extrusions are all tapped (see Fig. 51, Fig. 52, Fig. 53, Fig. 54, Fig. 55).

#### 5.4.2 Hole placement

**Fig. 56:**
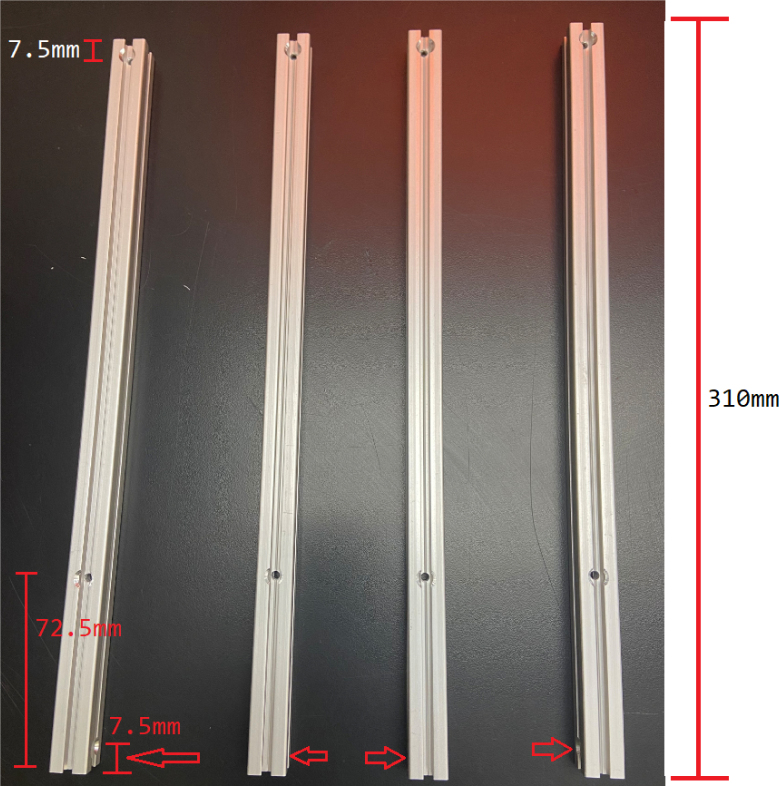
Hole placement locations for 310 mm extrusions.

**Fig. 57:**
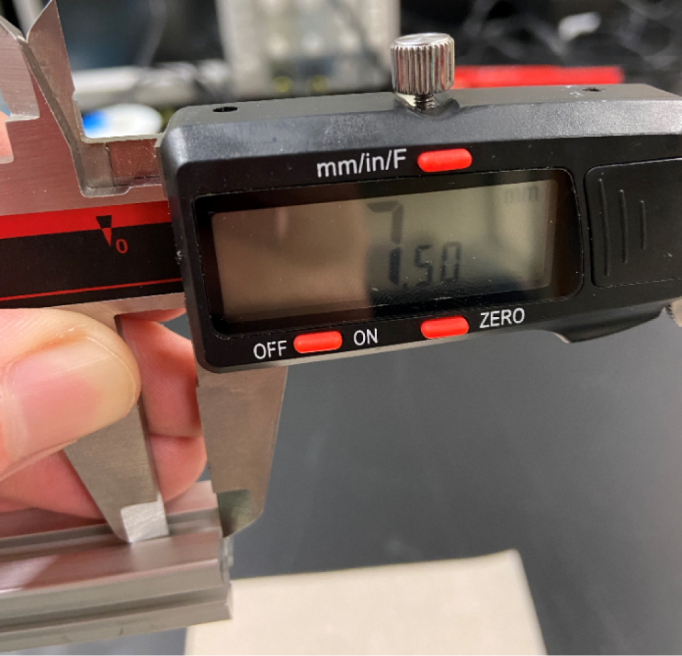
Caliper used with extrusion to determine hole locations.

**Fig. 58:**
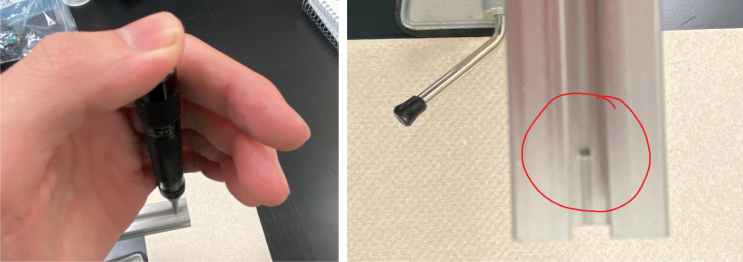
The center punch mark in hole location.

**Fig. 59:**
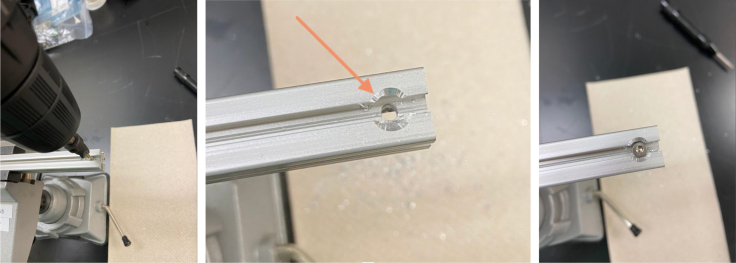
Extrusion with 3.2 mm hole from counter-sink.

1. For **two of the five** 610 mm Extrusions:**1x** hole **138.5 mm** from one side
2. For **two of the four** 200 mm Extrusions:**2x** hole **52.5 mm** from either side
3. For **the other two** 200 mm Extrusions:**1x** hole in the center of the extrusion (**138.5 mm** from each side)
4. For **all four** 310 mm Extrusions:**1x** hole **7.5 mm** from one side **1x** hole **72.5 mm** from other side
  (a) For **two of the four** 310 mm extrusions: Rotate the extrusion 90 degrees **clockwise** and on the end with the 72.5 mm hole **1x** hole **7.5 mm** from the edge
  (b) For the **other two** 310 mm extrusions: Rotate the extrusion 90 degrees **counterclockwise** and on the end with the 72.5 mm hole **1x** hole **7.5 mm** from the edge (see Fig. 56, Fig. 57, Fig. 58, Fig. 59).

#### 5.4.3 Drilling the holes

**Fig. 60:**
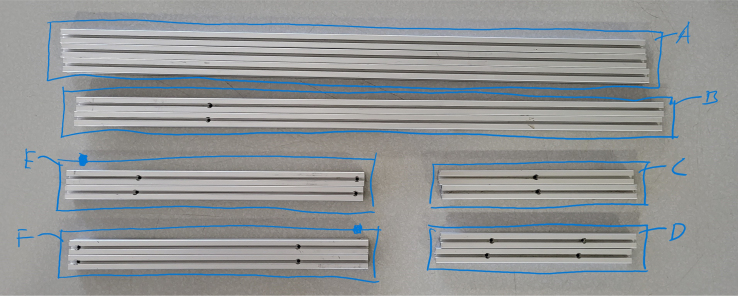
Hole locations for the pieces of extrusions.

1. Use the tip of the calipers to scratch a mark into the extrusion.
2. Use the center punch to create a nick at the precious marked location. This will help with drilling.
3. Drill a hole into the extrusion using the 3.2 mm Drill Bit with a countersink. Make sure that the counter-sink hole is large enough to fit an M3 bolt.
4. Repeat steps for the remainder of the hole locations indicated in the previous section (see Fig. 60).

#### 5.4.4 Assembling the frame

**Fig. 61:**
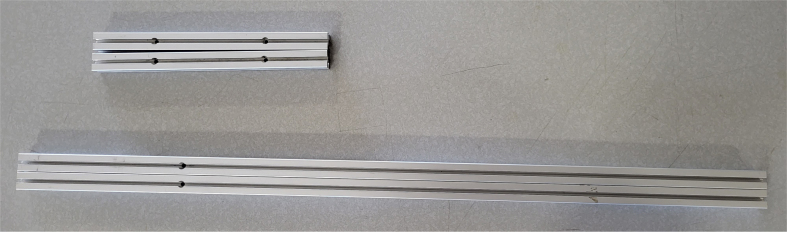
610 mm and 200 mm extrusion pieces.

**Fig. 62:**
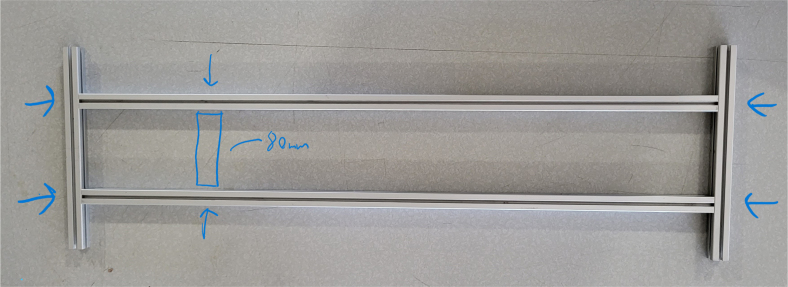
610 mm and 200 mm assembly with location of 80 mm extrusion.

**Fig. 63:**
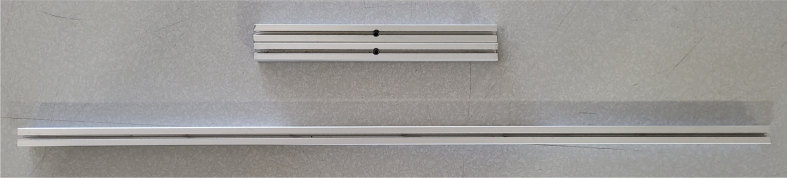
The 610 mm and 200 mm modified extrusion pieces.

**Fig. 64:**
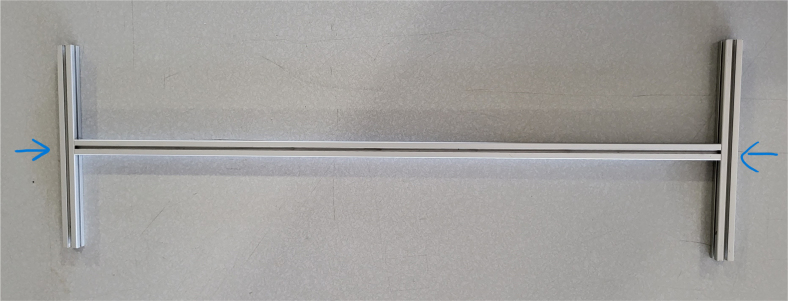
The 200 mm and 610 mm extrusion top frame sub-assembly.

**Note:** The arrows in the images denote the location/side of the countersunk holes. These arrows also indicate where the bolts should be put to attach the extrusions together. Do not tighten the bolts too much at this stage. The bolts will need to be taken out in later sections of the build guide (see Fig. 61, Fig. 62, Fig. 63, Fig. 64).

**Fig. 65:**
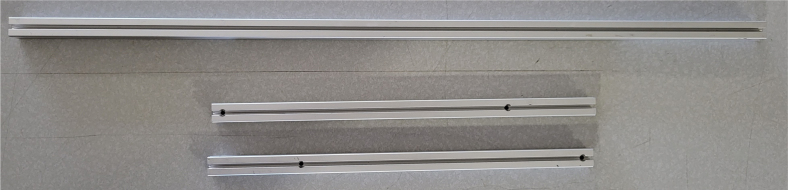
Extrusion A, Extrusion F, and Extrusion E.

**Fig. 66:**
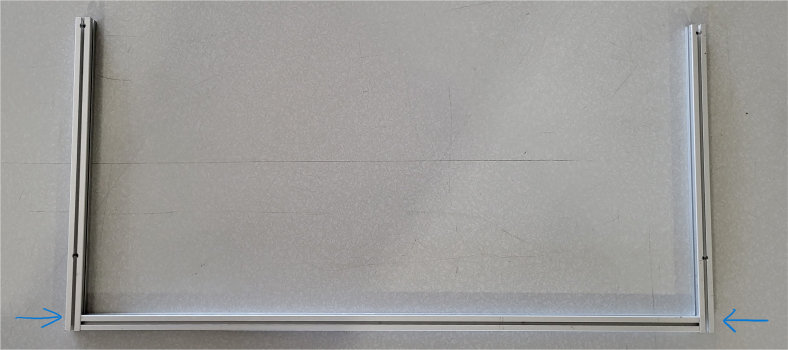
The 310 mm and 610 mm extrusion side frame sub-assembly.

**Fig. 67:**
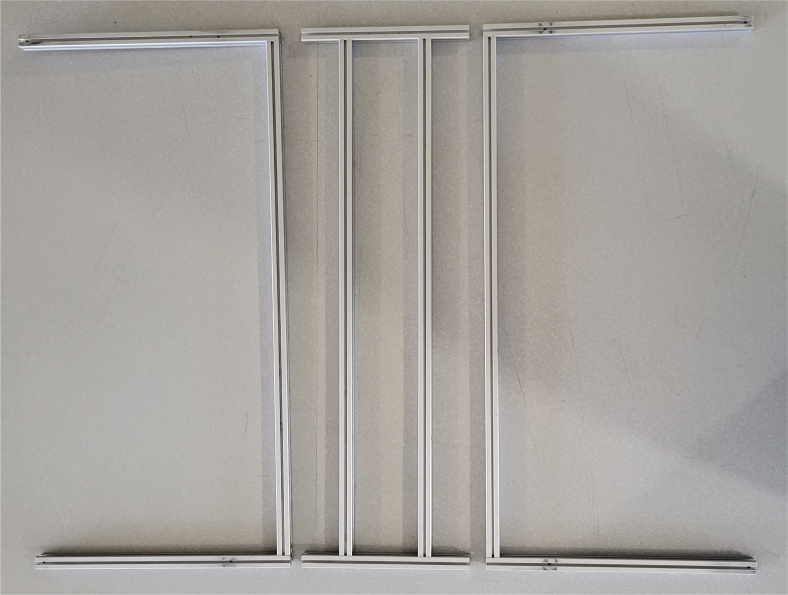
The left, bottom, and right frame sub-assemblies.

**Fig. 68:**
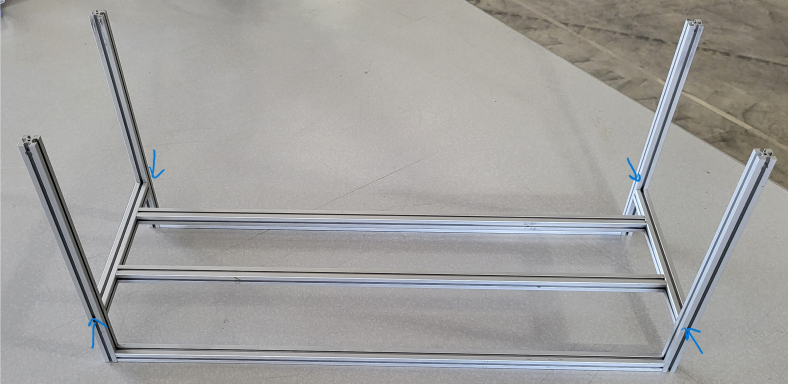
The lower frame sub-assembly.

**Fig. 69:**
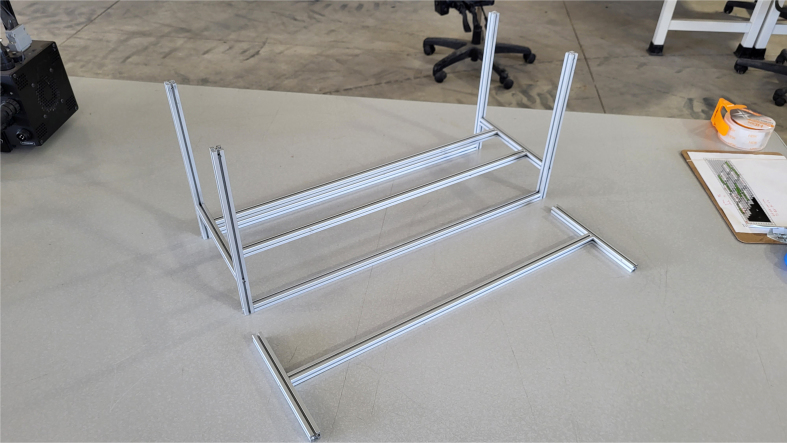
The lower and upper frame sub-assemblies.

**Fig. 70:**
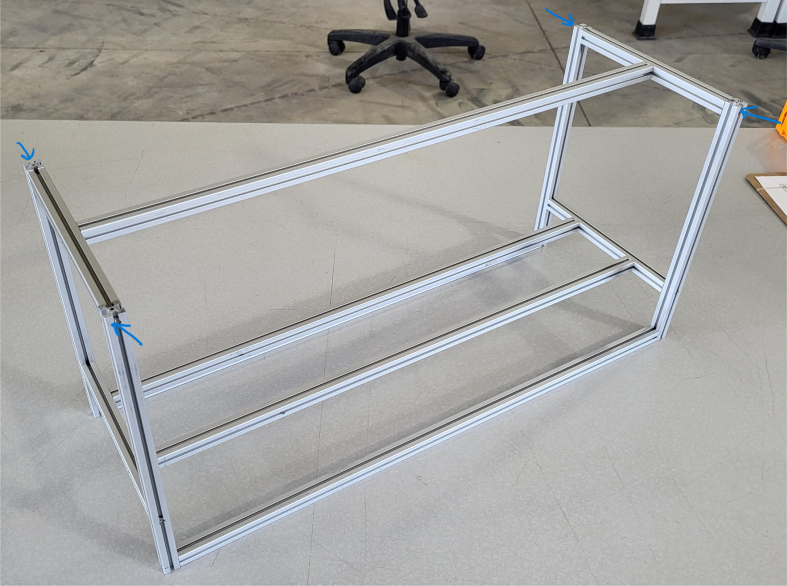
The fully assembled frame for the sampler.

1. Gather the following Extrusions:
  • 80 mm Extrusion
  • 2x Modified 610 mm Extrusion (Extrusion B)
  • 2x Modified (Two Holes) 200 mm Extrusion (Extrusion D)
2. Screw the two 610 mm extrusions that have holes in them into the 80 mm extrusion. Screw the **200 mm extrusions with two holes** on each side of the 610 mm extrusions.
3. Gather the following Extrusions:
  • Unmodified 610 mm Extrusion (Extrusion A)
  • 2x Modified (One Hole) 200 mm Extrusion (Extrusion C)
4. Screw the remaining two 200 mm extrusions on either side of a 610 mm extrusion.
5. Gather the following Extrusions:
  • Modified 310 mm Extrusion (Extrusion E)
  • Modified 310 mm Extrusion (Extrusion F)
  • Unmodified 610 mm Extrusion (Extrusion A)
6. Screw the 310 mm extrusions onto the remaining 610 mm extrusions.
  Make sure that the secondary holes of the 310 mm face outwards.
7. Gather the side and bottom sub-assemblies:
8. Screw the part made in Step 2 onto the parts from Step 4.
9. Gather the final two sub-assemblies:
10. Screw the part from Step 3 onto the part from Step 5 (see Fig. 65, Fig. 66, Fig. 67, Fig. 68, Fig. 69, Fig. 70).

### 5.5 Hydraulic rails and frame assembly prep

**Fig. 71:**
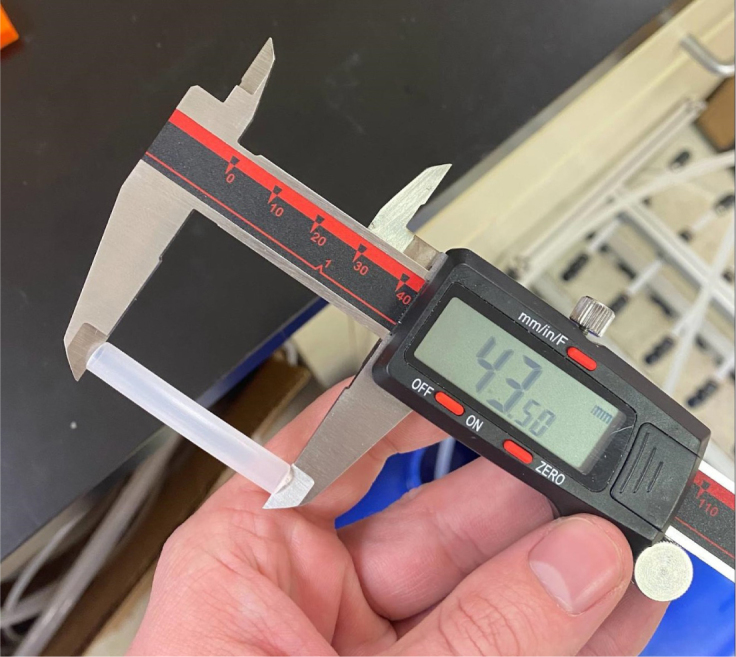
The Caliper measurements for hydraulic thin-walled tubing.

**Fig. 72:**
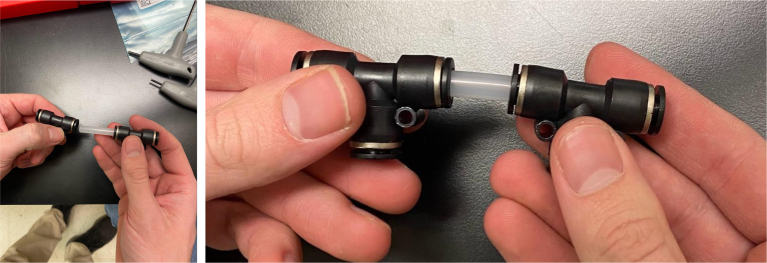
The top hydraulics push to connect cross sub-assembly.

The section covers the assembly of the three Hydraulic Rail, the hydraulic backbone of the sampler, and the addition of square nuts to the frame in preparation for the upcoming sections. The square nuts are added in this stage because the frame has to be partly disassembled anyway to add the hydraulic rails (see Fig. 71, Fig. 72).

#### 5.5.1 Top hydraulics

**Fig. 73:**
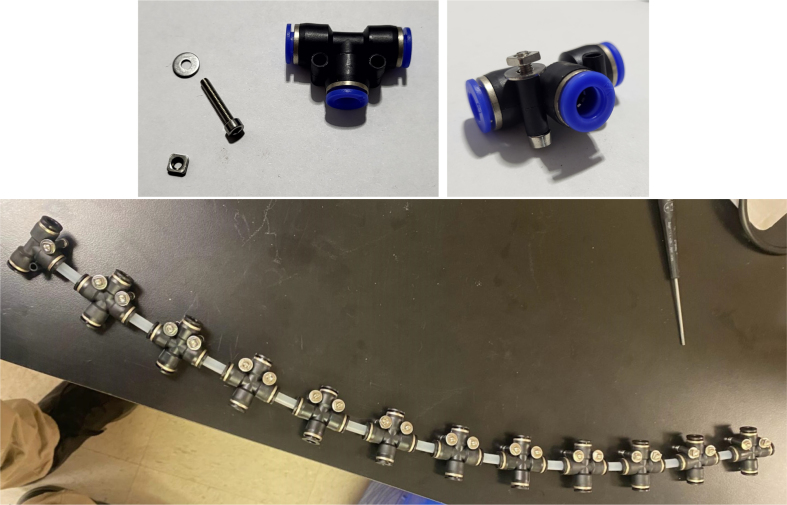
The bolts, washers, and square nuts inserted into The-push to connect cross sub-assembly.

**Fig. 74:**
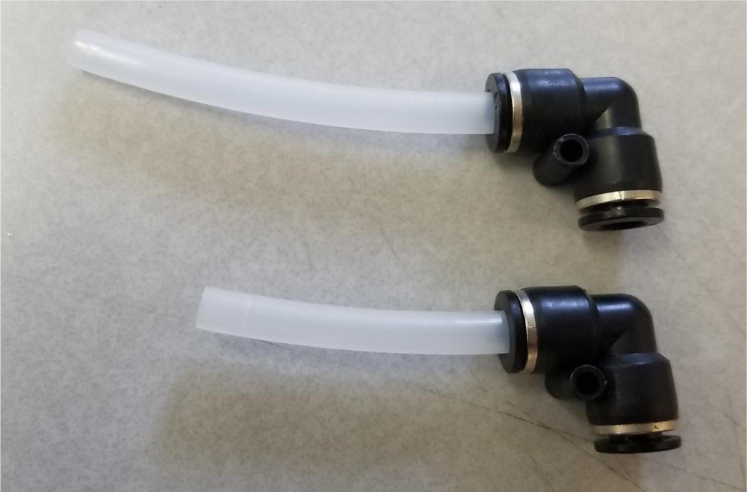
The top hydraulic 60 mm and 80 mm push to connect corner sub-assemblies.

**Fig. 75:**
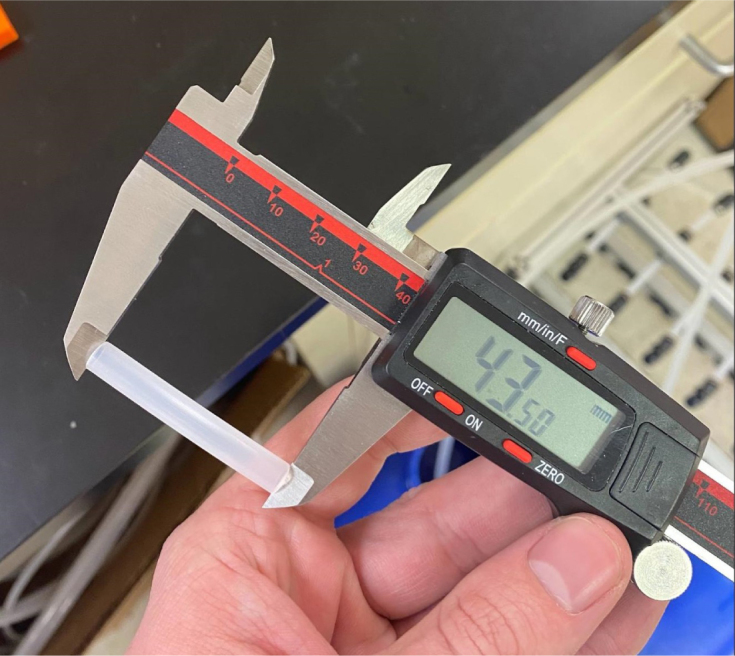
The Caliper measurements for hydraulic thin-walled tubing.

**Fig. 76:**
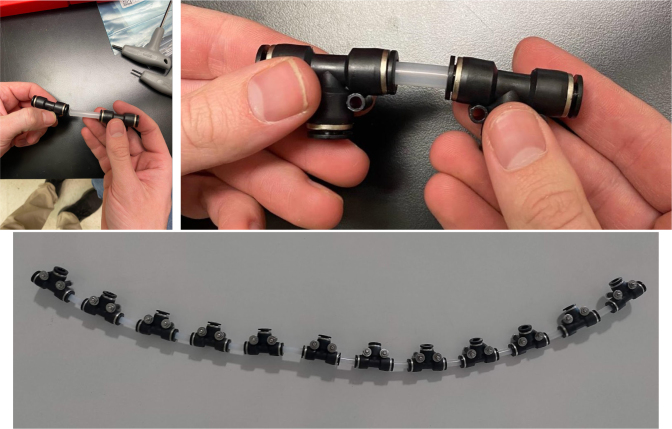
The bottom hydraulic push to connect tee sub-assemblies.

1. Using the calipers, verify that all of the Thin-Walled tubings are within 0.25 mm of the specified measurements.
2. Connect the 43.5 mm Thin-Walled Tubing to the Push-to-Connect Cross, creating one line of 11. Make sure that each tube is fully pushed in and is not kinked or damaged in any way.
3. Put the bolts, washers, and square nuts onto each of the connectors.
4. Take the 60 mm and 80 mm Thin-Walled Tubing and attach the Push-to-Connect Corners to each of the tubes (see Fig. 73, Fig. 74, Fig. 75, Fig. 76).

#### 5.5.2 Bottom hydraulics

**Fig. 77:**
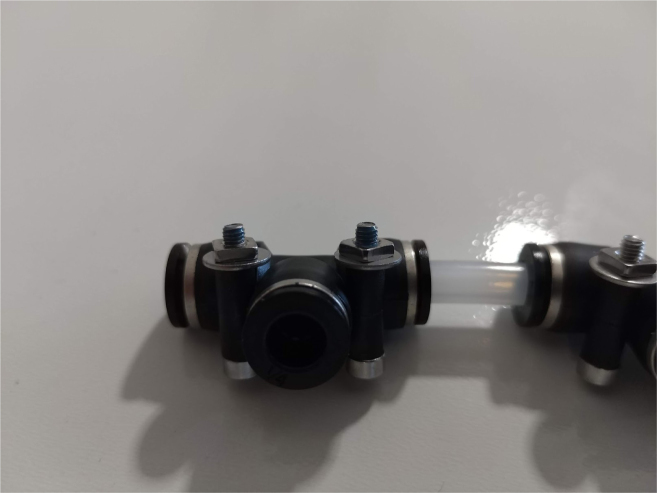
The M3 bolt, washers, and square nuts applied to the bottom hydraulic push to connect tee sub-assemblies.

**Fig. 78:**
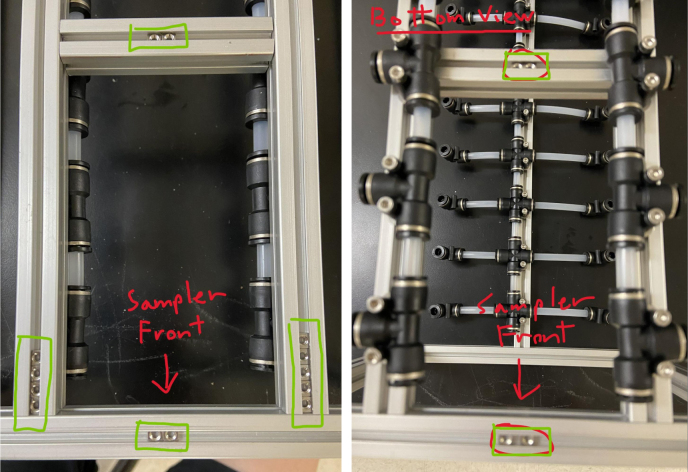
Square nuts applied to the top and bottom sampler frame.

**Fig. 79:**
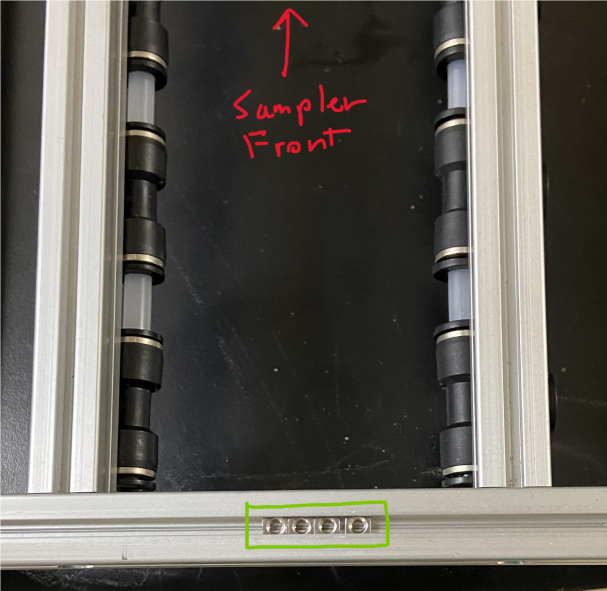
Square nuts applied to the top sampler frame.

**Fig. 80:**
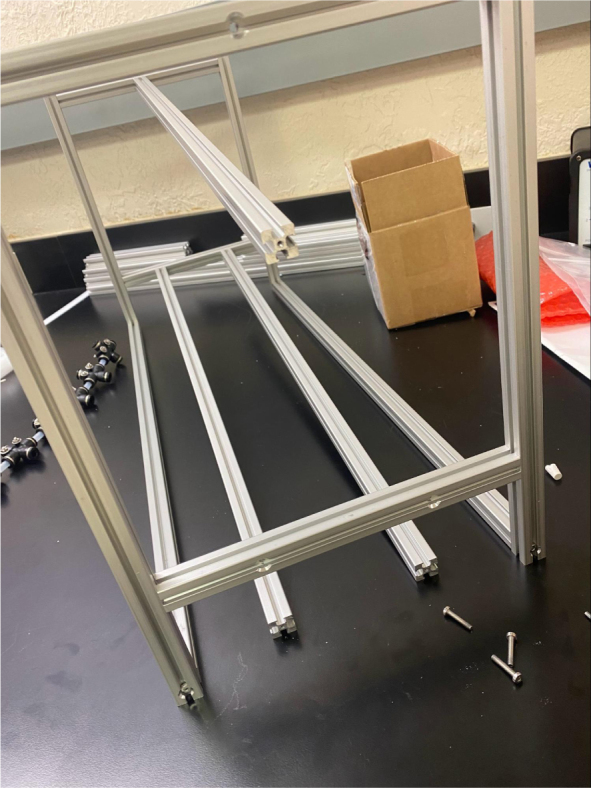
The sampler frame with bottom extrusion bolts removed.

1. Using the calipers, verify that all of the Thick-Walled tubing is within 0.25 mm of the specified measurement.
2. Make two lines of 12 Push-to-Connect Tees with tubing in between each Tee. Make sure that all of the tubing is fully pushed in and ensure that there are no kinks or damage done to the tubes.
3. Add M3 bolts, washers, and square nuts to each of the Push-to-Connects (see Fig. 77, Fig. 78, Fig. 79, Fig. 80).

#### 5.5.3 Frame assembly preparation

**Fig. 81:**
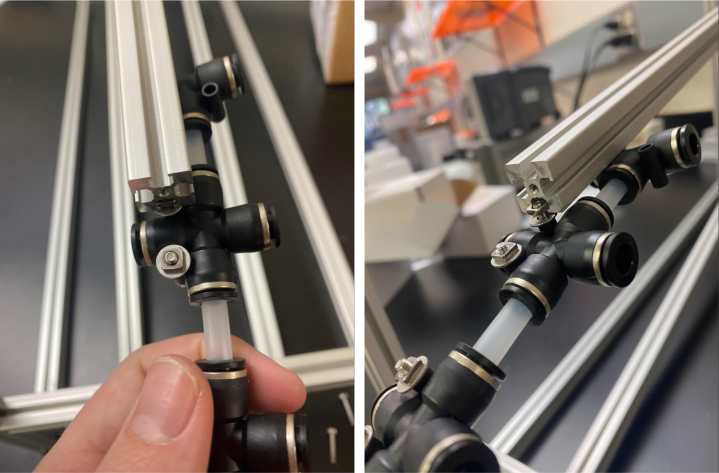
The top hydraulics inserted into the upper sampler frame.

**Fig. 82:**
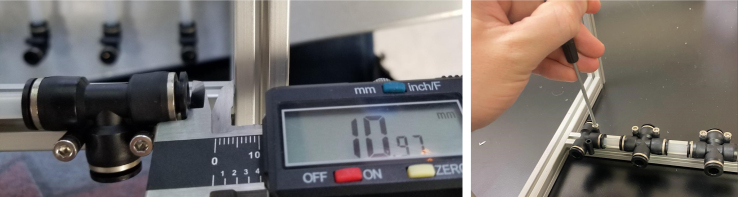
The upper hydraulics in correct frame position.

**Fig. 83:**
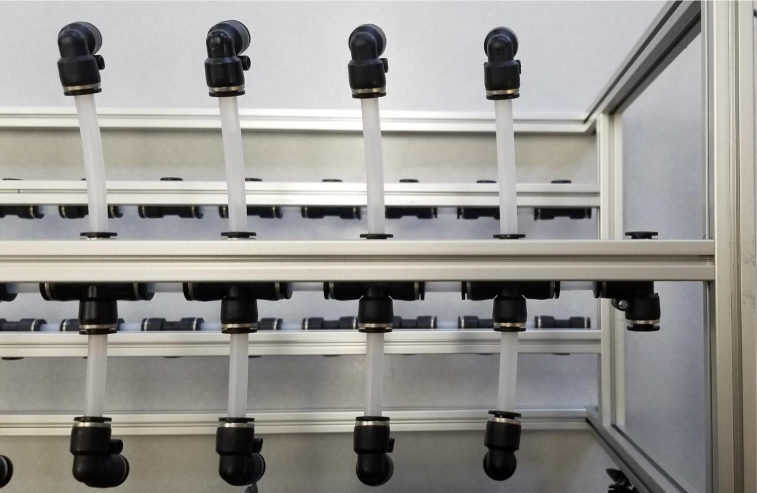
The corner push to connect sub-assemblies attached to the upper hydraulics.

**Fig. 84:**
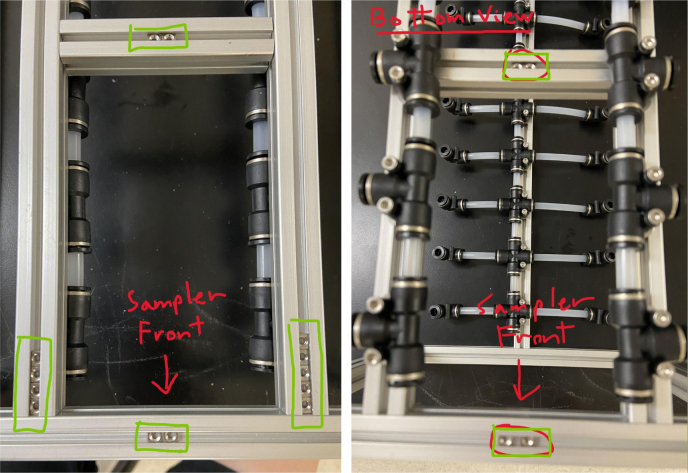
Square nuts applied to the top and bottom sampler frame.

**Fig. 85:**
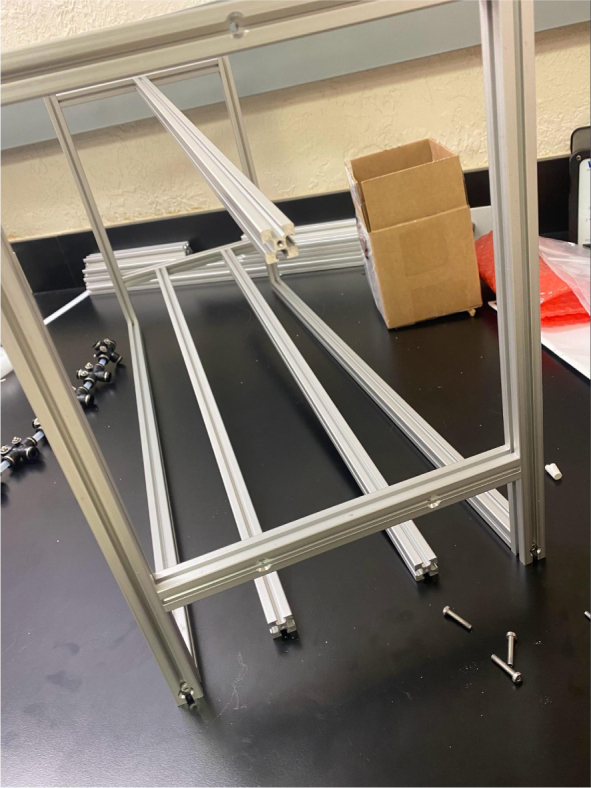
The sampler frame with bottom extrusion bolts removed.

In this section, we will be partially disassembling the frame to add the necessary components for later assembly sections. The “Top”, “Bottom”, etc positional descriptions are relative to the orientation of the Sampler Frame (see Fig. 81, Fig. 82, Fig. 83, Fig. 84, Fig. 85).

**Fig. 86:**
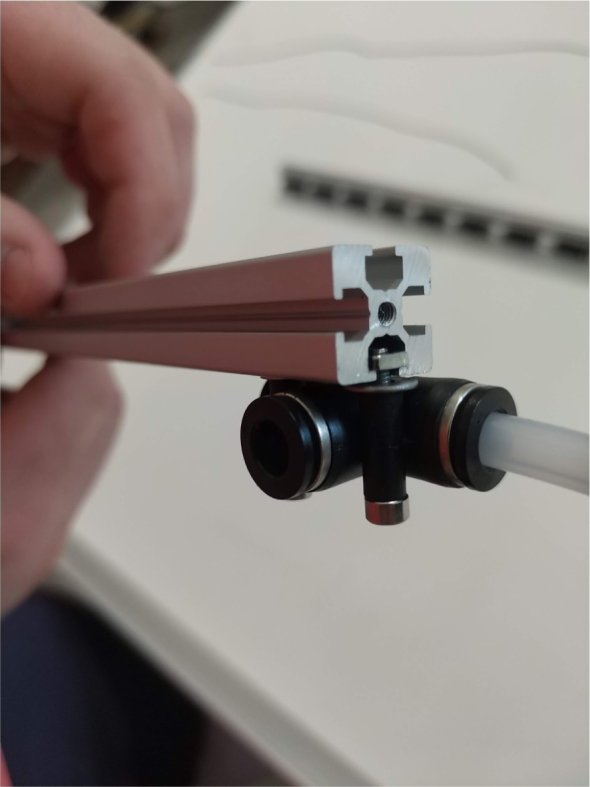
The bottom hydraulics inserted into the central bottom extrusion.

**Fig. 87:**
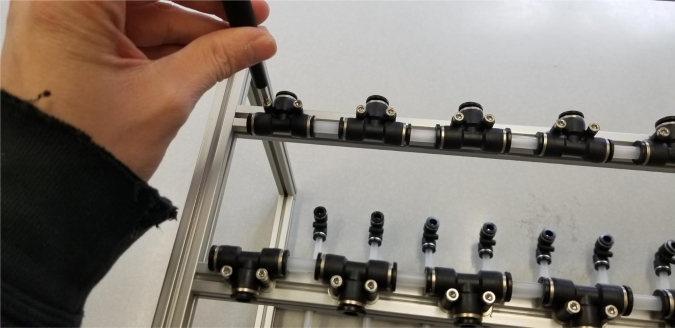
The bottom hydraulics tightened in correct position on frame.

**Fig. 88:**
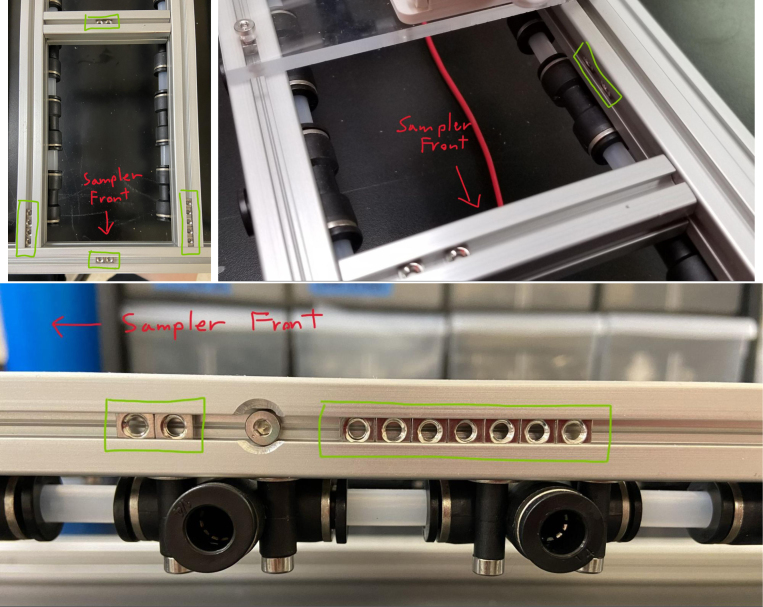
Square nuts applied to the top, left, and right of sampler frame.

**Fig. 89:**
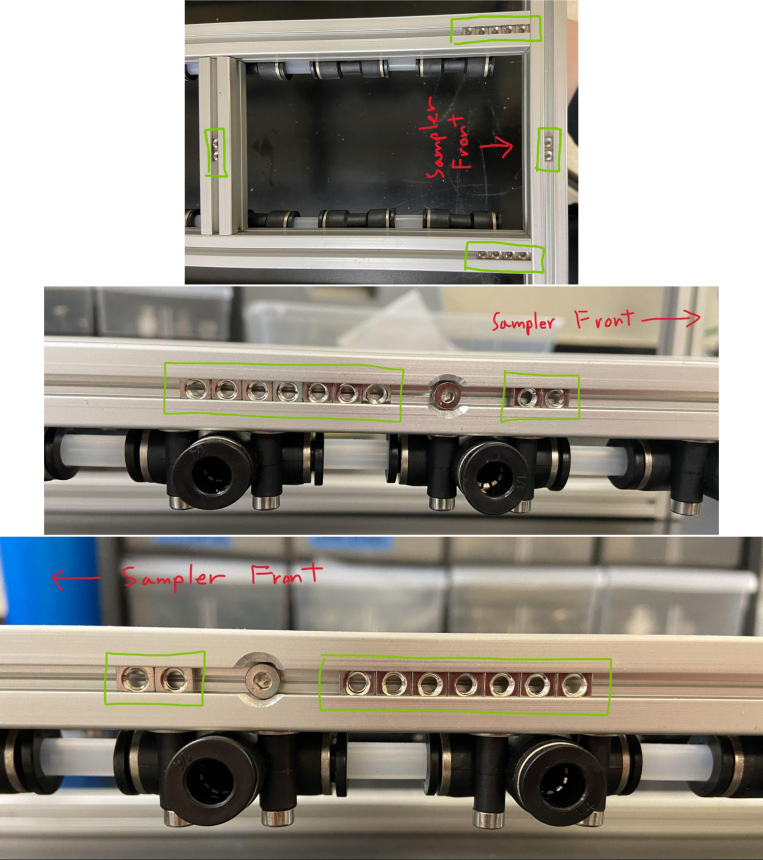
Square nuts applied to the top, and left of sampler frame.

**Fig. 90:**
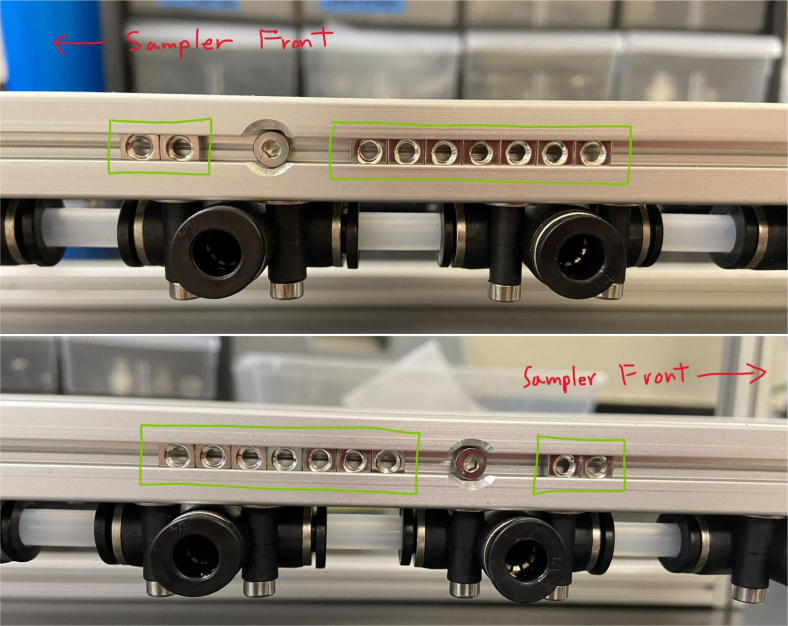
The final square nuts applied to the sampler frame.

1. Remove the Front-Bottom 200 mm Extrusion and add:
  2 Square nuts to the “Top” of the extrusion
  2 Square nuts to the “Bottom” of the extrusion
  Then reattach the extrusion.
2. Remove the Rear-Bottom 200 mm Extrusion and add:
  4 Square nuts to the “Top” of the extrusion
  Then reattach the extrusion.
3. Expose the Top 610 mm Extrusion by removing the bolt on one side of the sampler and tilting the extrusions downward.
4. Slowly slide the top Hydraulics onto the top metal extrusion of the frame. Be careful and make sure that the square nuts do not come off in the process.
5. Tighten the bolts down, making sure that the rail is placed 10 mm away from the perpendicular railing on the front of the frame (The side with the 80 mm extrusion).
  **NOTE:** The exact placement is less important than the alignment of the top and bottom hydraulics.
6. Take the 60 mm and 80 mm tube extensions that were made earlier and attach them to the top Hydraulics.
  Place the 60 mm extensions on the long side of the Push-to-Connects.
  Place the 80 mm extensions on the short side of the Push-to-Connects.
7. Reattach the Top 610 mm Extrusion
8. Remove the 80 mm Extrusion and add:
  2 Square Nuts to the “Top” of the extrusion
  2 Square Nuts to the “Bottom” of the extrusion
  Wait to reattach the 80 mm extrusion.
9. Expose the two Central 610 mm Extrusions by removing the bolts on one side of the sampler and tilting the extrusions downward.
10. Slowly slide the bottom Hydraulic rails onto the Central Extrusions carefully making sure the square nuts do not come off in the process.
11. Tighten the screws light so the rail will not move. Make sure that the rail is placed approximately 10 mm away from the perpendicular railing on the front (the side where the 80 mm extrusion is). This measurement is less important than making sure that the Bottom Hydraulic Rails are aligned with the Top Hydraulic Rail.
12. Add the following Square Nuts to the Central-Right 610 mm Extrusion:
  9 Square Nuts to the “Right” of the extrusion
  5 Square Nuts to the “Top” of the extrusion
  1 Square Nut to the “Left” of the extrusion
13. Add the following Square Nuts to the Central-Left 610 mm Extrusion:
  9 Square Nuts to the ”Left of the extrusion
  4 Square Nuts to the “Top” of the extrusion
14. Reattach the central 610 mm extrusions.
15. Reattach the 80 mm extrusion.
  Make sure that all of the Square Nuts are toward the back of the sampler **except** for 2 of the 9 Square nuts on both central extrusions. This is because the bolt for the 80 mm extrusion blocks the movement of the Square Nuts (see Fig. 86, Fig. 87, Fig. 88, Fig. 89, Fig. 90).

### 5.6 Central assembly

**Fig. 91:**
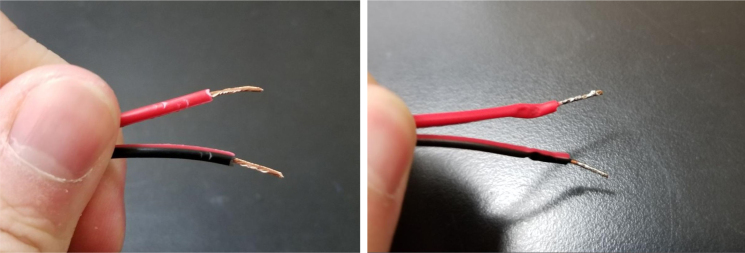
Stripped AWG wire with solder applied.

**Fig. 92:**
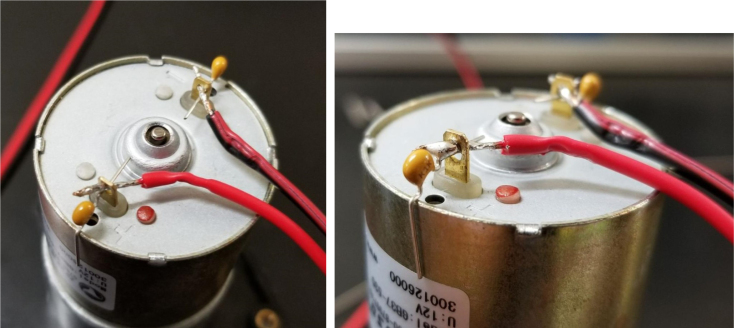
The pump with soldered to the AWG wires and 150pF capacitors.

**Fig. 93:**
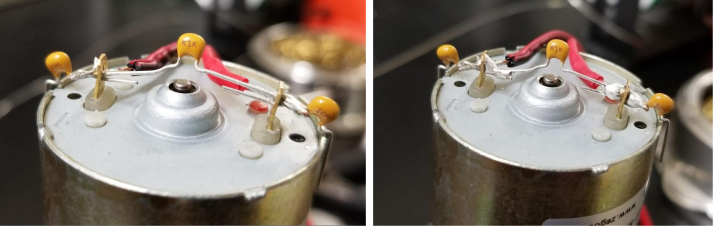
A third capacitor soldered bridging the original 2 capacitors.

This section covers the assembly and attachment of all of the components that sit in the center of the sampler to the frame. This includes the Battery, Pump, Ball Valve, Alcohol Valve, Air Valve, Purge Valve, and all the hydraulics to connect them all together (see Fig. 91, Fig. 92, Fig. 93).

#### 5.6.1 Preparing the pump

**Fig. 94:**
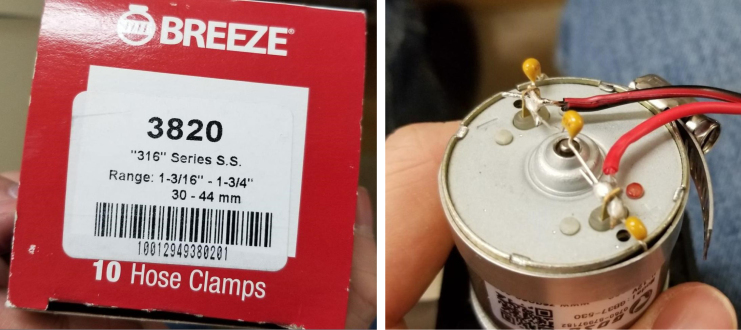
A hose clamp applied to the pump assembly.

**Fig. 95:**
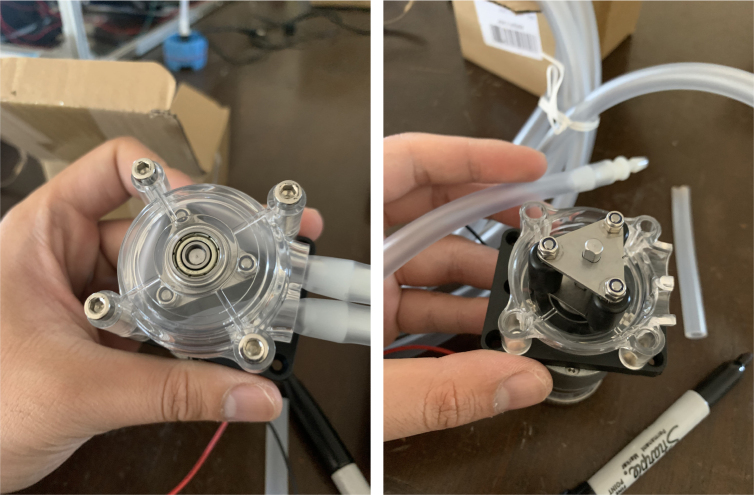
The pump opened by removing the 4 top bolts.

**Fig. 96:**
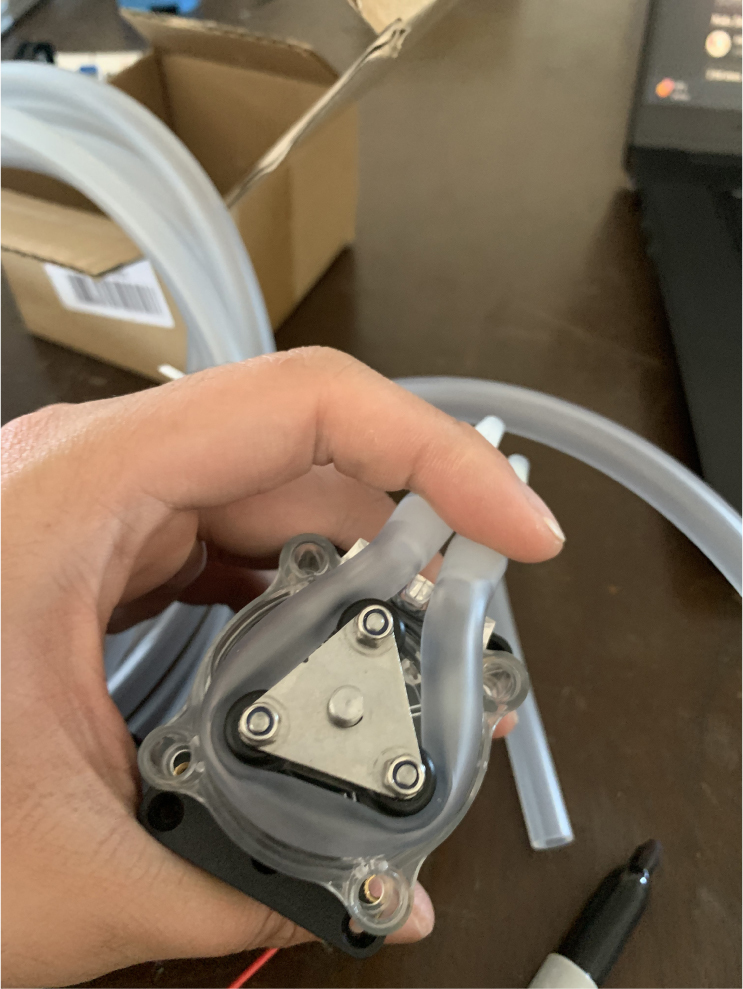
Pump tubing inserted into pump.

**Fig. 97:**
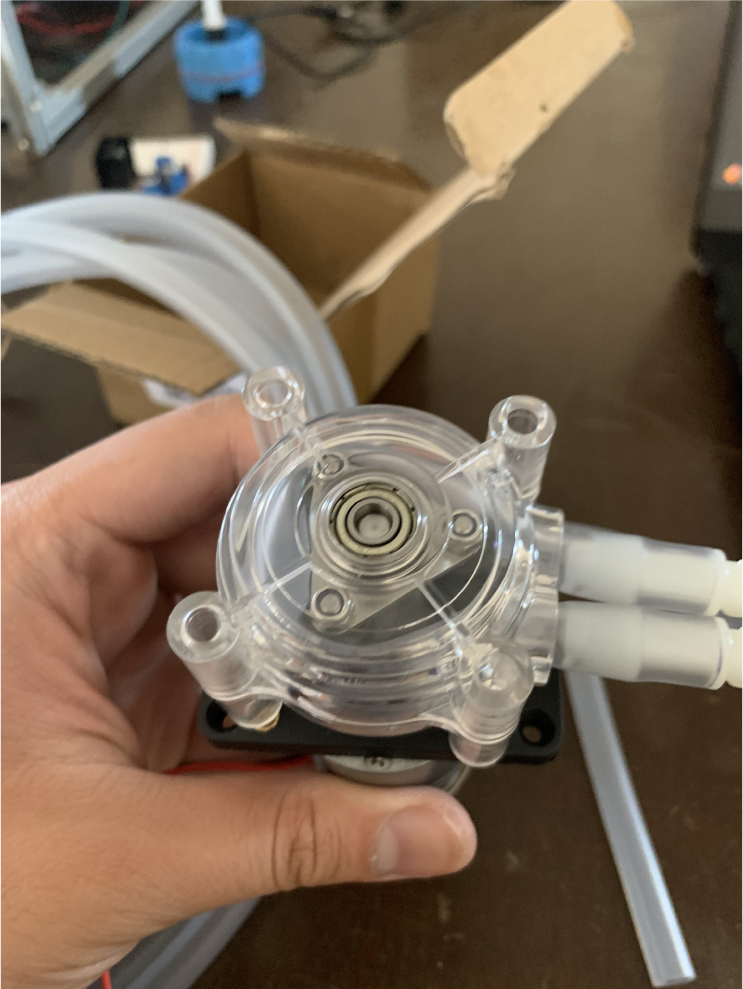
Closed-pump with barbed tubing.

**Fig. 98:**
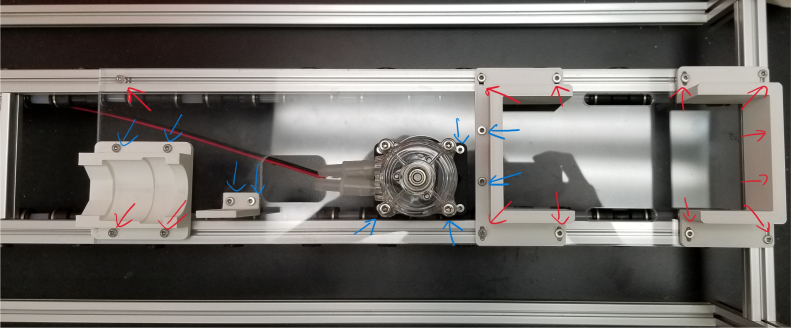
Outlining how the 3D Prints are placed and which bolts use the Square Nuts.

**Fig. 99:**
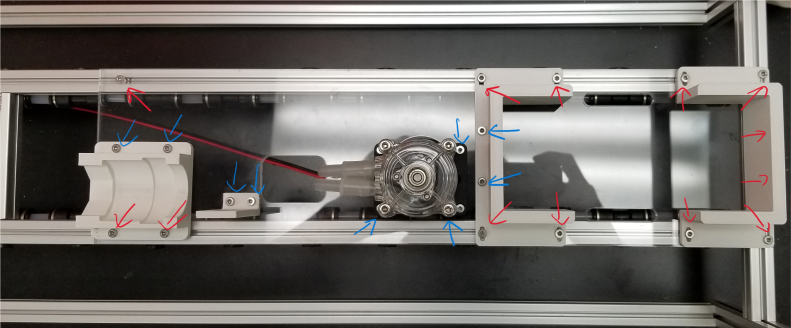
Outlining how the 3D Prints are placed and which holes use the Nylock Nuts.

**Fig. 100:**
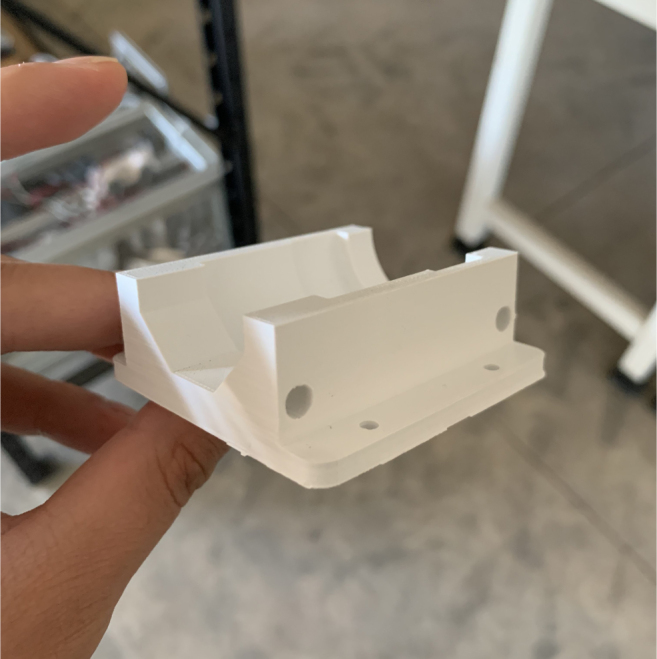
The ball valve mount with supports removed.

**Fig. 101:**
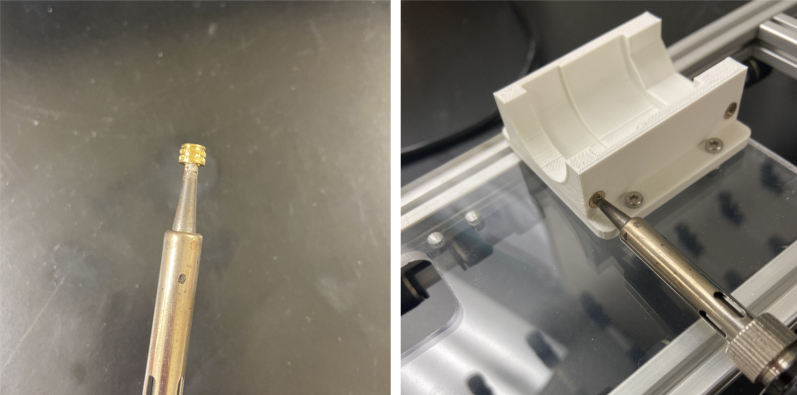
The ball valve mount with heated insets.

**Fig. 102:**
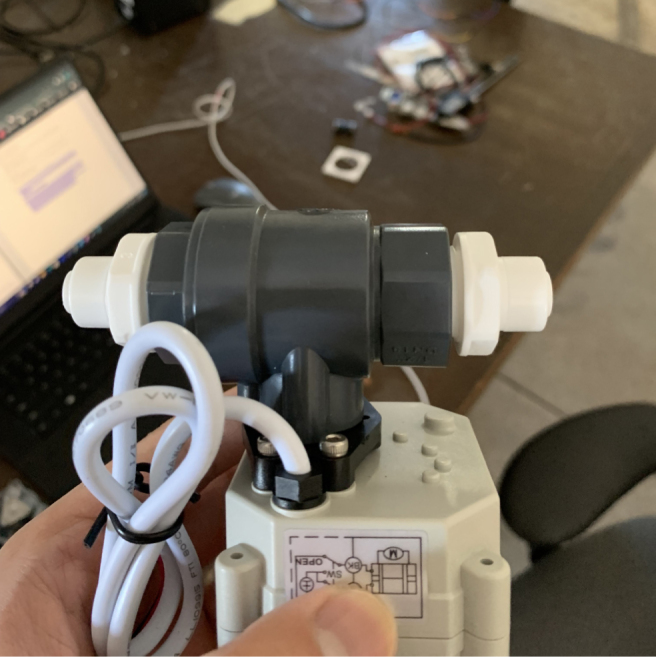
The ball valve with push to connect converters.

**Fig. 103:**
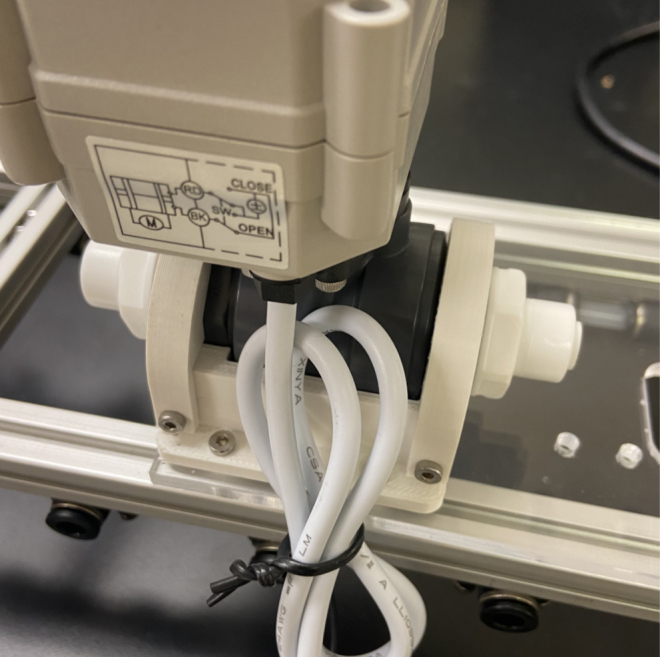
The ball valve mounted with M3x8 mm bolts.

**Fig. 104:**
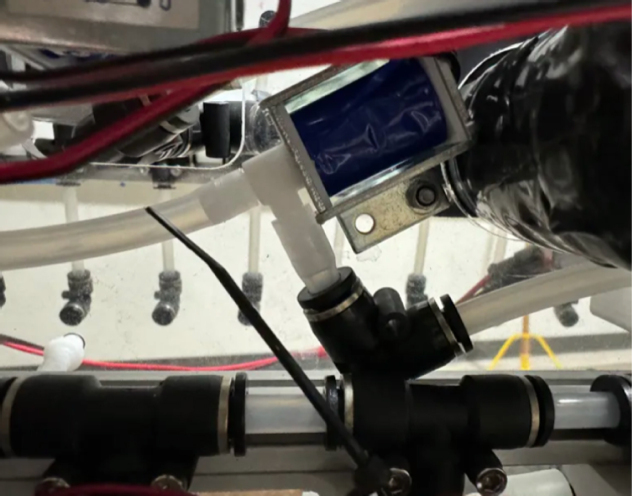
The air valve mounted to the sampler.

1. Cut a $\frac{1}{2}$ meter length of 22 AWG wire and prepare the Wire by stripping the insulation and tinning the wire (adding solder to the exposed wire).
2. Solder the wires and 2 150pF capacitors to the terminals of the pump.
  One lead of the capacitor will be attached to the terminal and the other will touch the side of the pump.
3. Use a multi-meter to verify that everything is electrically connected correctly.
4. Use a multi-meter to verify that everything is electrically connected correctly.
5. Attach a Hose Clamp onto the pump being sure that the leads of the side capacitors are being clamped to the side of the pump.
6. Unscrew the bolts holding the top of the pump down and remove the top of the pump and the tubing.
7. Cut a piece of pump tubing long enough to fit inside the pump with 1 in remaining on each side and attach the barb converter to the ends of the tubing.
8. Insert the Tubing into the pump housing and reattach the top of the pump.
9. Insert the M3 × 16 mm Counter-sunk bolts into the mounting holes of the pump (see Fig. 94, Fig. 95, Fig. 96, Fig. 97, Fig. 98, Fig. 99, Fig. 100, Fig. 101, Fig. 102, Fig. 103, Fig. 104).

#### 5.6.2 Assembling the central components

**Fig. 105:**
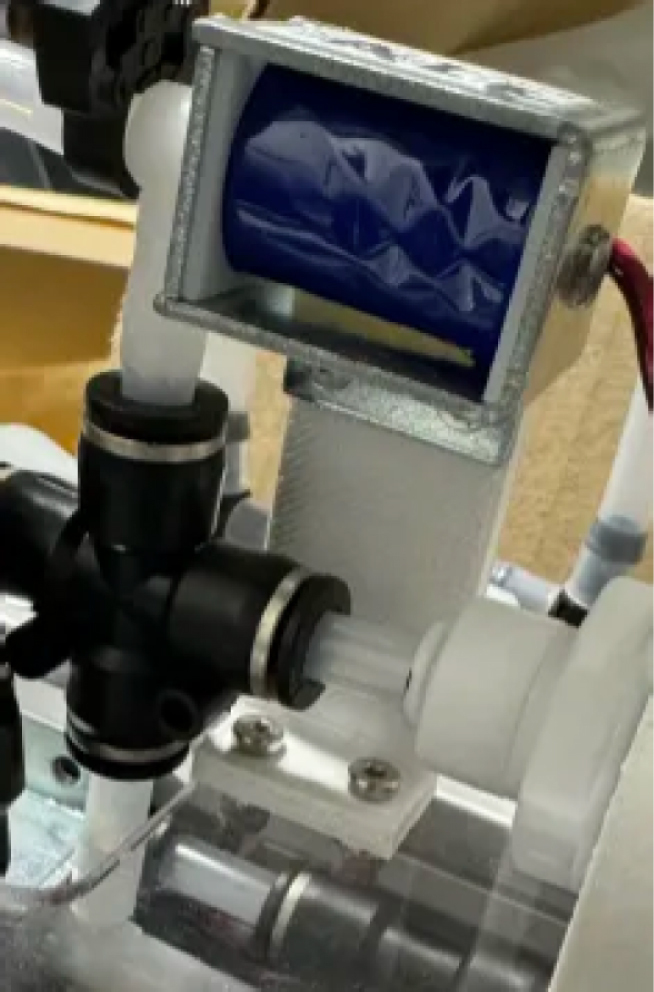
The L-bracket mounted to the Acrylic base plate.

**Fig. 106:**
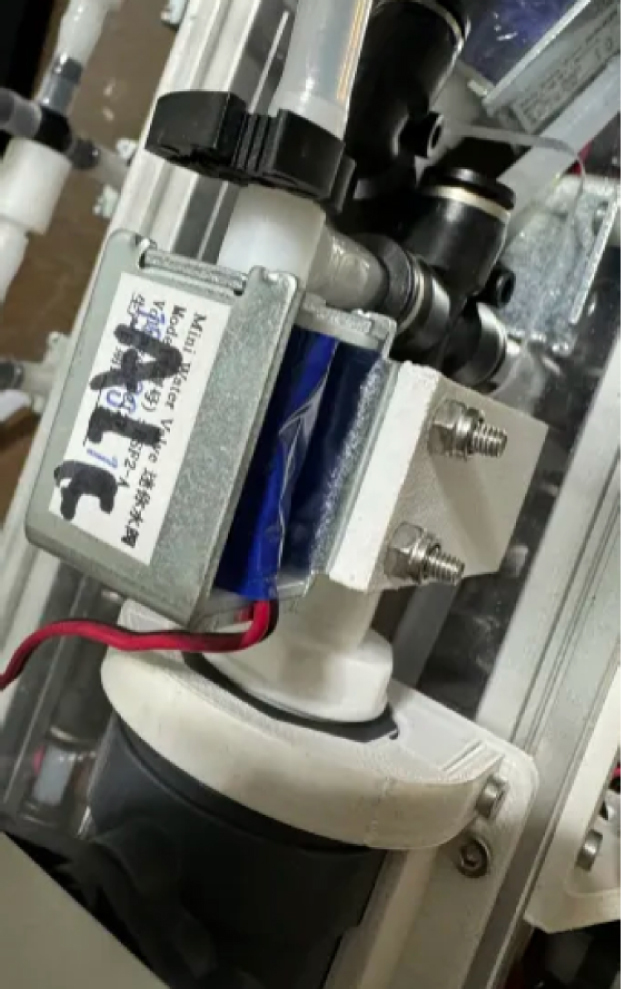
The alcohol valve mounted to the L-bracket.

1. Align all of the Square Nuts on the tops of the two Central Extrusions and the rear extrusion so that they line up with the holes on the Acrylic Base Plate.
2. When attaching the Acrylic Base Plate, the Battery Holder, and Ball Valve Mounts need to be attached at the same time. This is because the 3D Prints share mounting hardware with the Acrylic Plate.
  -Red indicates the bolts that go into the M3 Square Nuts
3. Some of the mounting holes for the Battery and Ball Valve mounts also need the Nylock Nuts which will be added in this step.
  The Pump and Preservative Valve Hanger will also be added in this step since there are mounting with Nylock Nuts as well.
  -Blue indicates the bolts that go into the M3 Nylock Nuts
  **Note:** The top-let Pump bolt does not have an arrow indicator. This is because a Nylock Nut will be added later in this section.
4. On the sides of the Ball Valve Mount, there should be four holes (two per side) that may be filled with 3D Printing Support Material. Clear out the support material as this space is the location for the M3 heated inserts.
5. Set the soldering iron to 160 ℃. Once heated, use the soldering iron to push the heated inserts into the Ball Valve Mount. Do not force it in, apply light pressure and allow the heated insert to melt its way in. Ensure that the heated inserts are flush to the side of the mount.
  **WARNING:** Use caution in this step to avoid burn injuries.
6. Attach the two $\frac{1}{4}$” NPT to Push-to-Connect converters to the Ball Valve.
7. Attach and secure the Ball Valve to the Mount using M3 × 8 mm Bolts.
  **Note:** Insert Orientation Note.
8. Attach air valve to the last motor bolt
  Use one M3 × 12 screw with a Nylock nut to secure it to one of the motor bolts near the large, square-rounded hole, positioning it at approximately a 45-degree angle to the left, as shown in the picture.
9. Attach 3d printed L bracket to acrylic
  Use two M3 × 12 screws with M3 nylon nuts to secure the component to the acrylic base.
10. Secure the alcohol valve to the L-bracket using two M3 × 12 screws with M3 nylon nuts (see Fig. 105, Fig. 106).

#### 5.6.3 Assembling the central hydraulics

Since there are a lot of tubes that need to be cut in this section, the format of many steps will contain a short description of each tube needed in the step, the length of that tube, and what kind of tube it is. The description will simply be where the two ends of the tube connect i.e. Barb Converter (Pump) to Push-to-Connect Corner: **34 mm** (Thin-Walled).

While some tubes will be attached to Push-to-Connects, other tubes will need to be attached to barb fittings. Simply heat the ends of the tubing with a heat gun and push them onto the barbs like in the previous sections of this build guide.

**Fig. 107:**
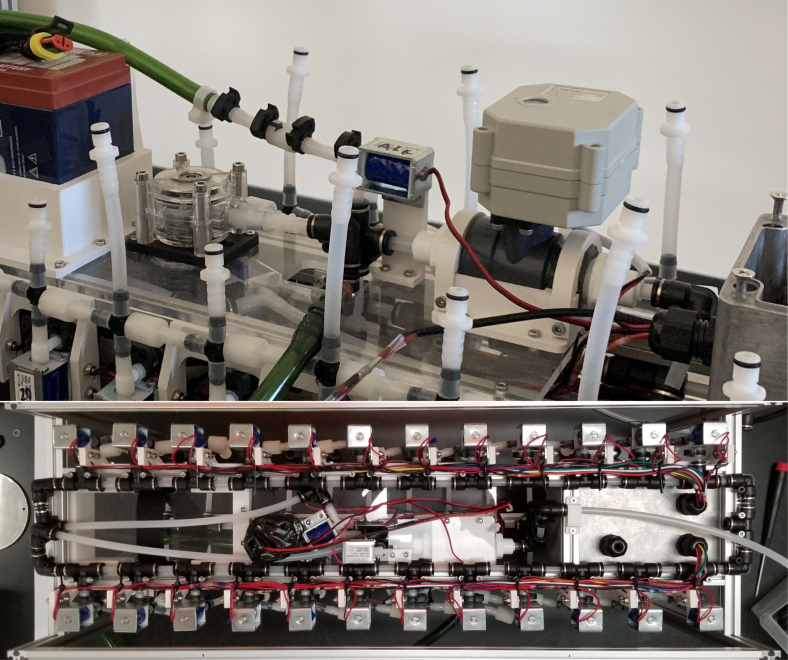
The hydraulics mounted above the acrylic plate and below the sampler.

**Fig. 108:**
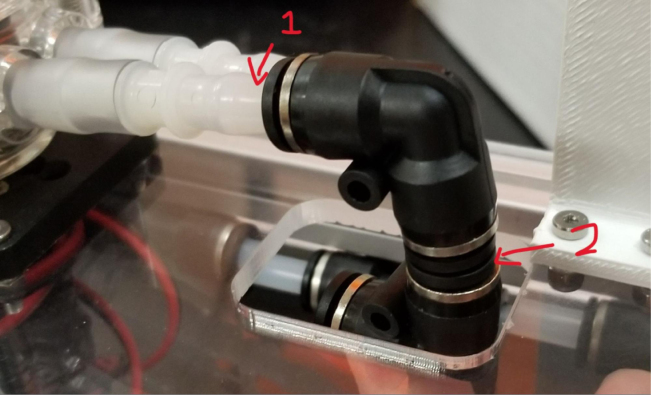
The pump barb converter connected to push to connect corners.

**Fig. 109:**
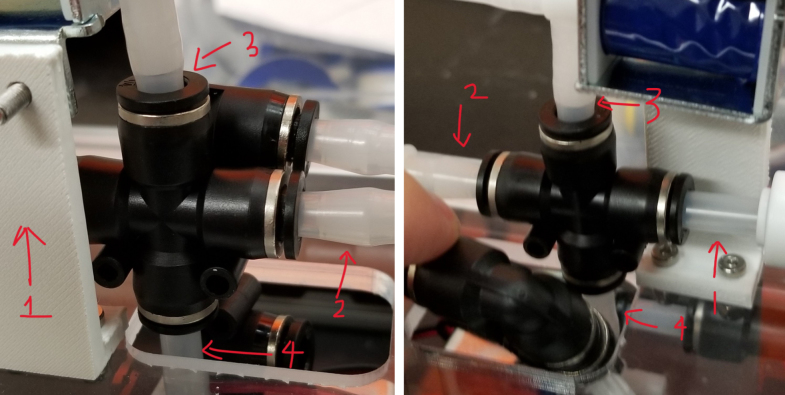
The pump input hydraulics connected to push to connect crosses.

**Fig. 110:**
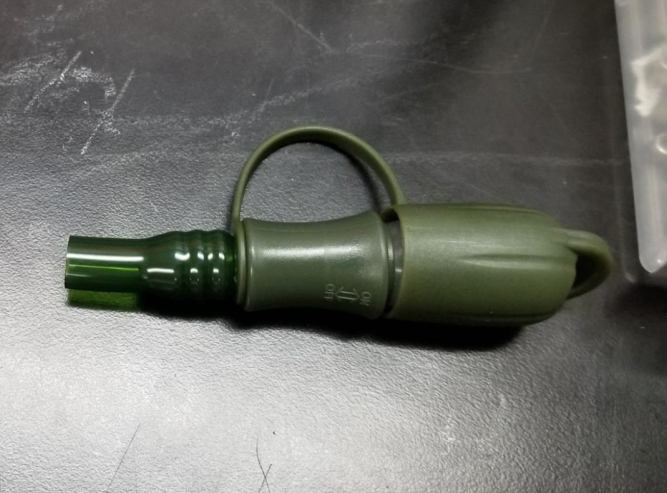
The Preservative tube component to be removed.

**Fig. 111:**
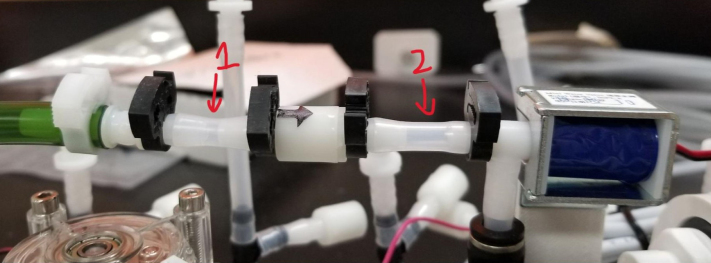
The preservative hydraulic sub-assembly.

**Fig. 112:**
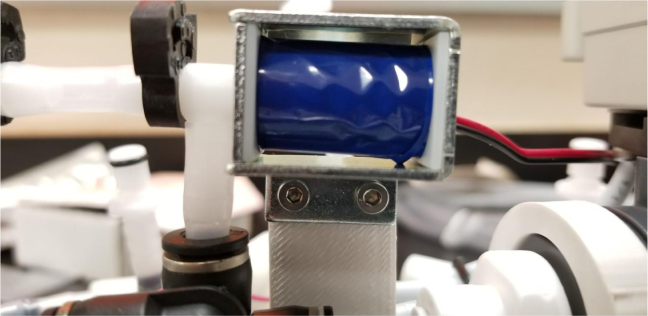
The preservative valve mounter in the correct position.

**Note:** PtoC will be used as a shorthand for Push-to-Connect as many are used in this section (see Fig. 107, Fig. 108, Fig. 109, Fig. 110, Fig. 111, Fig. 112).

**Fig. 113:**
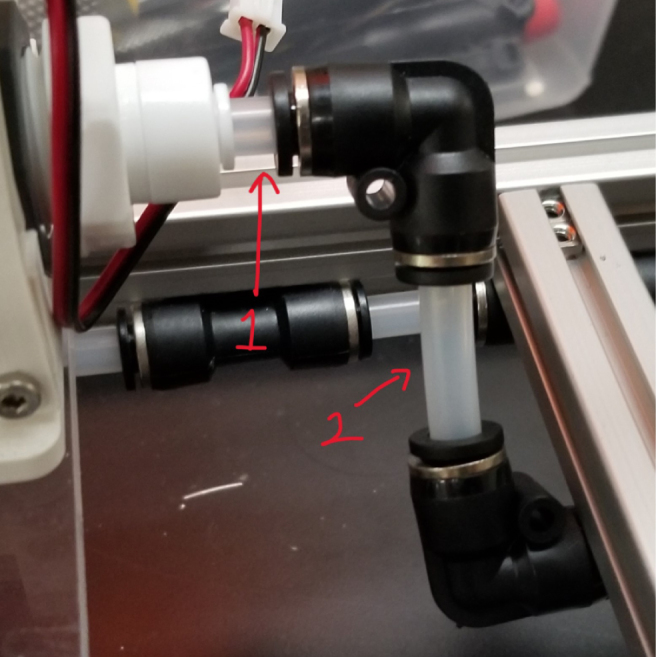
The ball valve input hydraulic sub-assembly.

**Fig. 114:**
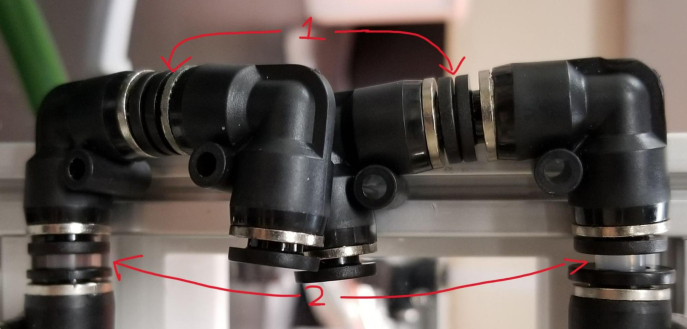
The back end lower hydraulic sub-assembly.

**Fig. 115:**
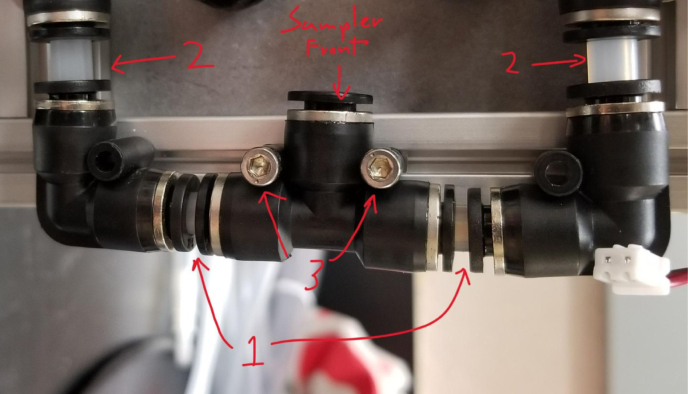
The front end lower hydraulic sub-assembly.

**Fig. 116:**
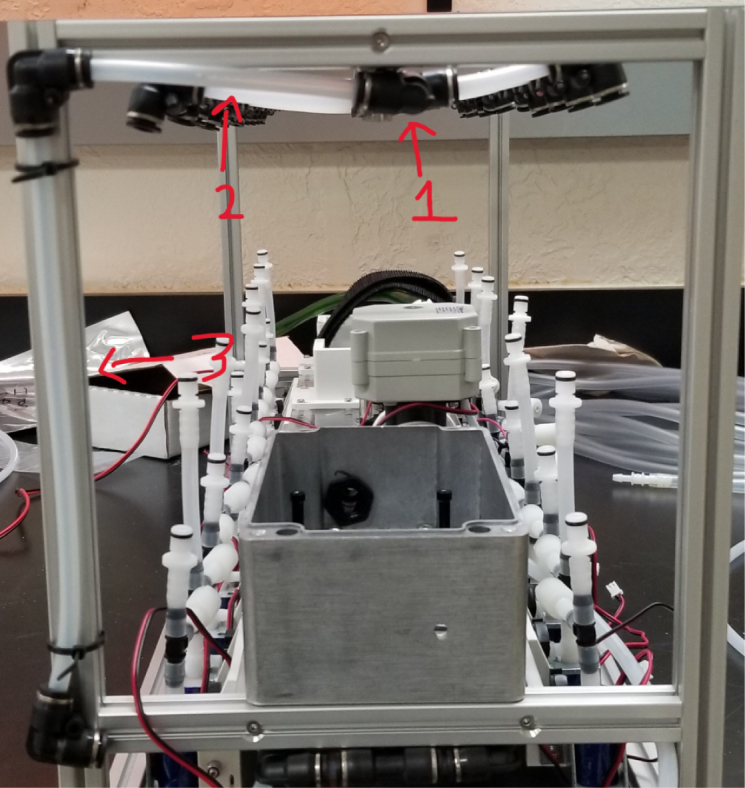
The output hydraulic sub-assembly.

**Fig. 117:**
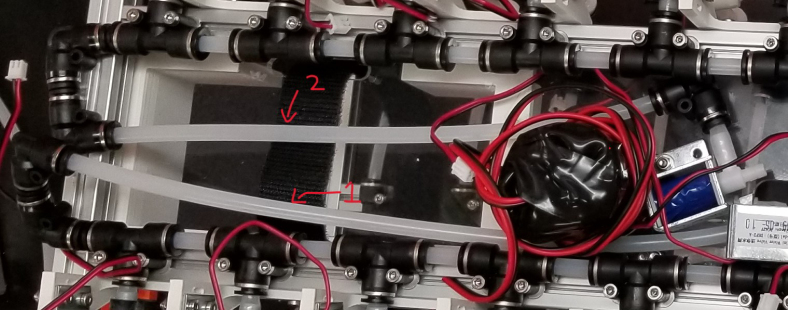
The final central hydraulics assembled.

1. **Pump Output Hydraulics**
  **Tube 1:** Barb Converter (Pump) to Top PtoC Corner - **34 mm** - Thin-Walled
  **Tube 2:** Top PtoC Corner to Bottom PtoC Corner - **34 mm** - Thick Walled
2. **Pump Input Hydraulics**
  **Tube 1:** Ball Valve to PtoC Cross - **45 mm** - Thin Walled
  **Tube 2:** Barb Converter (Pump) to PtoC Cross - **34 mm** - Thin Walled
  **Tube 3:** PtoC Cross to Top (Preserve) Valve - **32 mm** - Thin Walled
  **Tube 4:** PtoC Cross to Bottom (Air) Valve - **40 mm** - Thin Walled
  **Note:** It is recommended that Tube 1 is attached first. Tube 2 can then be inserted afterward since the pump tubing is very flexible
  **Note:** Tubes 3 and 4 require adding the tubes to the valves before they are attached.
3. Cut off the thicker end of the Preservative Tube as pictured.
  Attach the now exposed end of the Preservative Tube to a Barb Converter and add a Tube Clamp.
4. **Preservative Hydraulics**
  **Tube 1:** Barb Converter to Check Valve - **34 mm** - Thin Walled
  **Tube 2:** Check Valve to Preservative Valve - **34 mm** - Thin Walled
  **Note:** Ensure that the Check Valve is pointing towards the Valve (note the arrow direction).
5. Attach the Preservative Valve to its Mount using 2 M3 × 12 mm Bolts and 2 Nylock Nuts.
6. **Ball Valve Input Hydraulics**
  **Tube 1:** Ball Valve to PtoC Corner - **38 mm** - Thin Walled
  **Tube 2:** PtoC Corner to PtoC Corner (input side) - **50 mm** - Thin Walled
7. **Back End Lower Hydraulics**
  **2x Tube 1:** PtoC Corner to PtoC Corner - **34 mm** - Thick Walled
  **2x Tube 2:** PtoC Corner to Lower Rail (PtoC Tee) - **34 mm** - Thick Walled
8. **Front End Lower Hydraulics**
  **2x Tube 1:** PtoC Corner to Pto C Tee (For Sensor) - **32 mm** - Thick Walled
  **2x Tube 2:** PtoC Corner to Rail (PtoC Tee) - **37 mm** - Thick Walled
  Attach the PtoC tee (the one for the pressure sensor) to the extrusion using 2 M3 × 18 Bolts with washers. The square nuts should already be in place.
9. **Output Hydraulics**
  **Tube 1:** Top Rail (PtoC Cross) to PtoC Corner - **34 mm** - Thin Walled
  **Tube 2:** PtoC Corner (Rail Side) to PtoC Corner (Top-Left) - **113 mm** - Thin Walled
  **Tube 3:** PtoC Corner (Top-Left) to PtoC Corner (Bottom-Right) - **215 mm** - Thin Walled
  **Tube 4:** PtoC Corner (Bottom-Right) - **??mm** - Thin Walled
10. **Final Central Hydraulics**
  **Tube 1:** Motor Output to Bottom Hydraulics
  **Tube 2:** Bottom Hydraulics to Purge Valve
  **Tube 3:** Purge Valve to output (Purge Tube) (see Fig. 113, Fig. 114, Fig. 115, Fig. 116, Fig. 117).

### 5.7 Final mechanical assembly

**Fig. 118:**
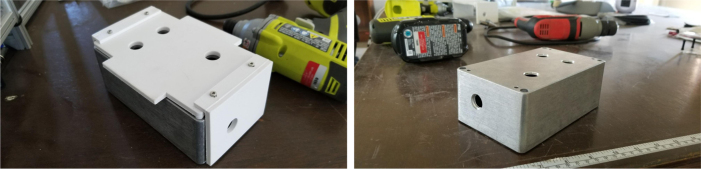
Electronics box holes cut using the 3D printed Jig.

**Fig. 119:**
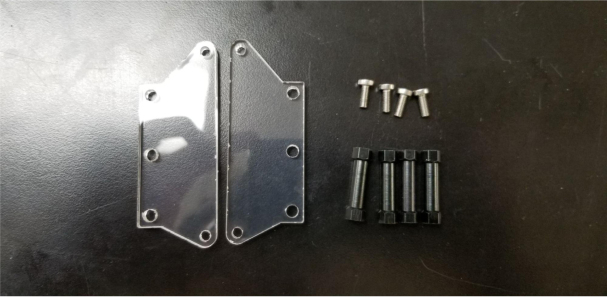
Electronics box assembly components.

**Fig. 120:**
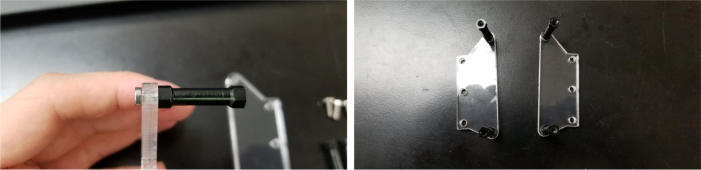
Acrylic pieces with M3 standoffs attached.

**Fig. 121:**
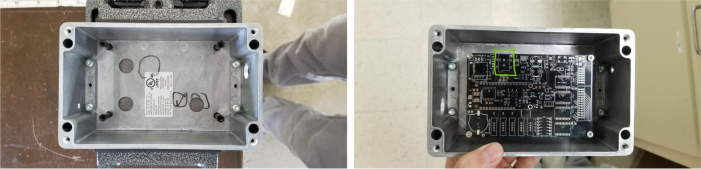
The self-threading screws mounting the acrylic stand and electronics board.

**Fig. 122:**
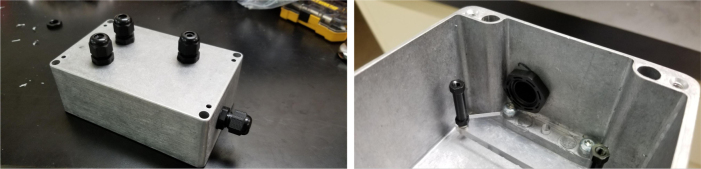
The cable glands attached to the electronics box.

This section covers the “final” bits of assembly to the frame of the sampler, minus wiring and other electronics-related assembly. This includes the assembly/attachment of the Valve Hangers, Electronics Box, and Pre-Filter (see Fig. 118, Fig. 119, Fig. 120, Fig. 121, Fig. 122).

#### 5.7.1 Assembling the electronics box

**Fig. 123:**
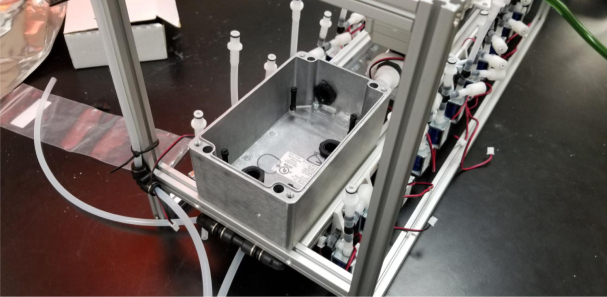
The electronics box mounted to the frame.

**Fig. 124:**
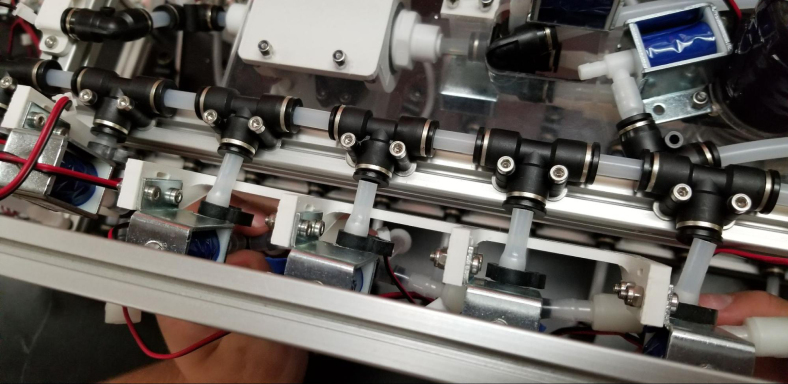
The valve hangers aligned with the lower hydraulic rails.

**Fig. 125:**
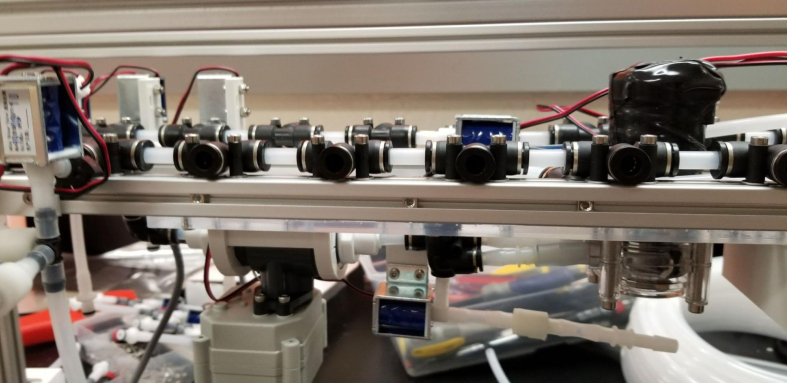
The frame square nuts aligned with the valve hangers.

1. Use the 3D Printed Template/Jig to cut the necessary holes in the electronics box.
2. Gather the parts pictured below.
3. Attach the 25 mm M3 Standoffs to the acrylic using the M3 × 8 Bolts.
  Make sure that both acrylic pieces are mirrors of each other.
4. Use the Self-Threading Screws to attach the acrylic mounts to the electronics box.
  **Note:** The mounts are specifically oriented like in the images below to make room for the power plug.
5. Attach the PG9 Cable glands to the Electronics Box.
  The rear PG9 Cable gland will need to be attached slightly differently due to the greater wall thickness. The nut needs to be flipped around for the threads to catch.
6. Pre-align the nuts on the frame with the electronics box mounting holes.
  Attach the Electronics Box to the Frame using M3 × 12 mm bolts.
  Make sure that the smaller hole (the one for the switch) is pointing toward the front of the sampler (see Fig. 123, Fig. 124, Fig. 125).

#### 5.7.2 Attaching the valve hangers

**Fig. 126:**
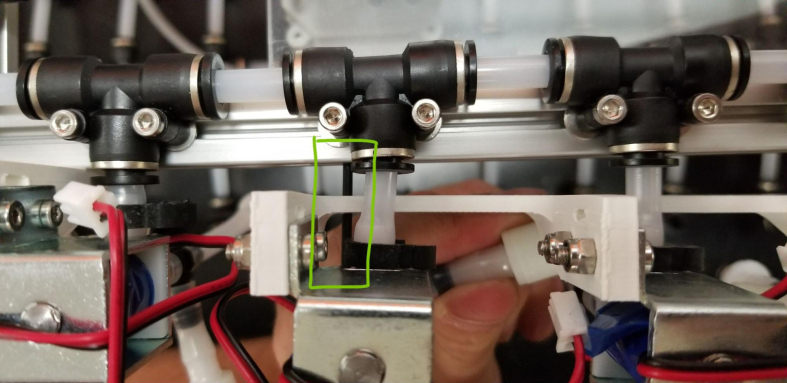
The valve hanger aligned with corresponding square nuts.

The following steps will need to be repeated for each Valve Hanger. Be careful not to accidentally trap any square nuts when attaching the Valve Hangers. There are just enough square nuts to attach the valve hangers and it is difficult to add more, so if any are trapped, anything between the trapped nut and where it needs to go will need to be removed (see Fig. 126).

**Note:** When attaching the Valve Hangers, the valves with the short tubes need to face the front of the sampler. This is where the orientation of the Valve Hangers matter.

**Fig. 127:**
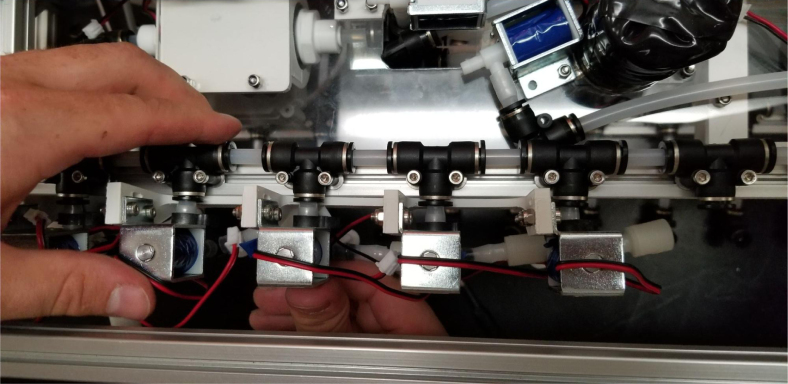
The valve hanger mounted to the frame.

**Fig. 128:**
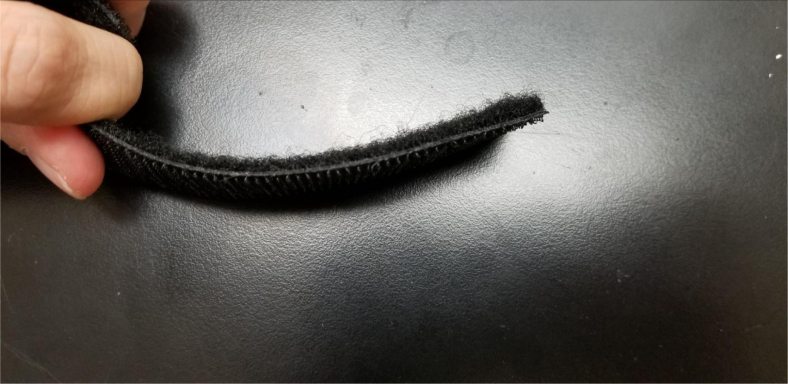
The assembled battery Velcro.

**Fig. 129:**
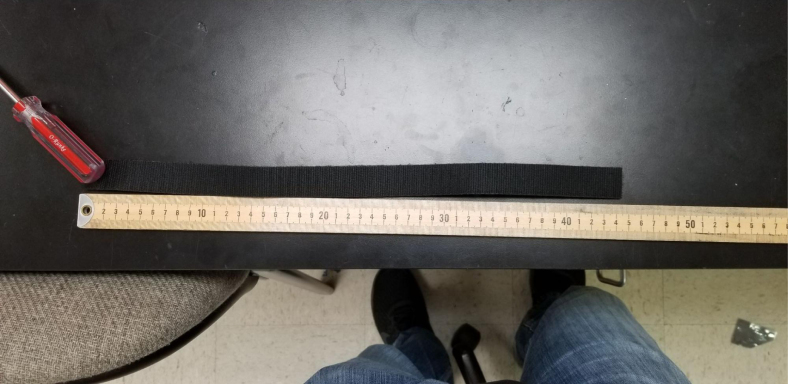
The assembled Velcro cut to 45 mm.

1. Align the tubes on the Valve Hanger with the PtoC Tees of the Hydraulic Rails.
2. Position the square nuts approximately where the valve hanger’s holes are located.
3. Use the screwdriver to fully align the square nuts with the holes in the valve hanger assembly.
4. Once all three nuts are aligned correctly, fully insert the valve hanger into the frame. Use the M3 × 8 mm bolts to secure the valve hangers to the frame.
  **Note:** If any of the square nuts are misaligned while attaching the bolts, it is recommended to either retry the previous steps or to tilt the sampler slightly and try to shift the nuts into place (see Fig. 127, Fig. 128, Fig. 129).

#### 5.7.3 Battery velcro

**Fig. 130:**
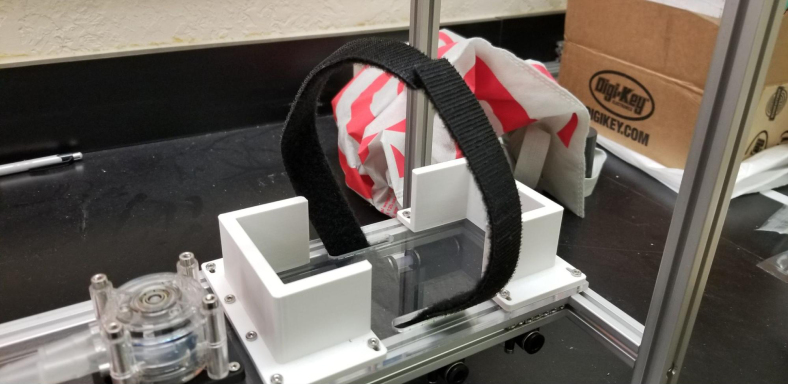
The battery Velcro in correct position on battery mount.

**Fig. 131:**
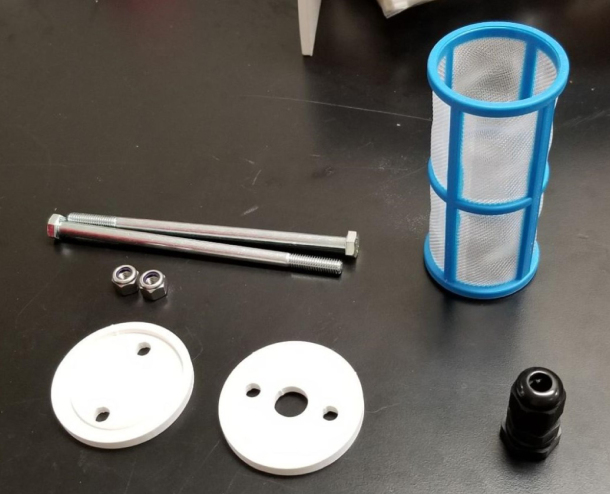
The pre-filter sub-assembly components.

**Fig. 132:**
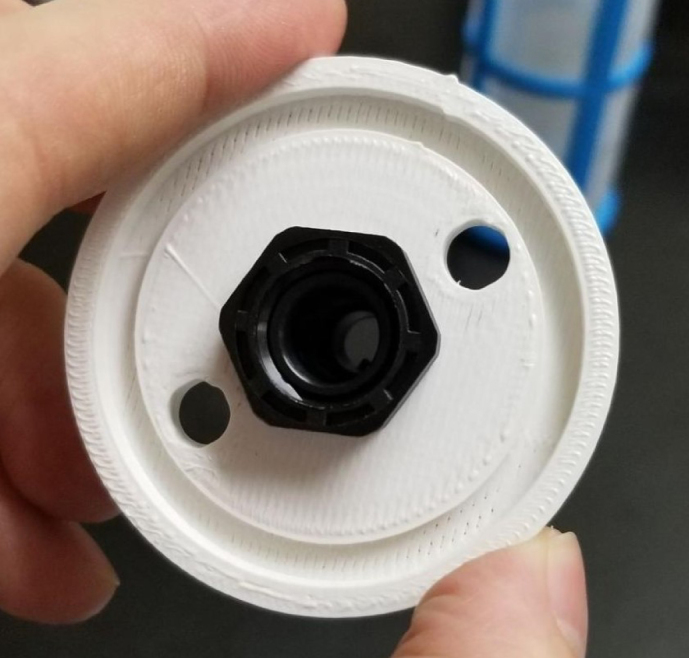
The PG7 glad mounted to the 3D printed lid.

1. Take the two sides of the Velcro and attach them back-to-back (Adhesives side together).
2. Cut a length of about 45 mm (18in). This does not need to be exact.
3. Push the piece of Velcro through the slits between the Battery Mounts (see Fig. 130, Fig. 131, Fig. 132).

#### 5.7.4 Assembling the pre-filter

**Fig. 133:**
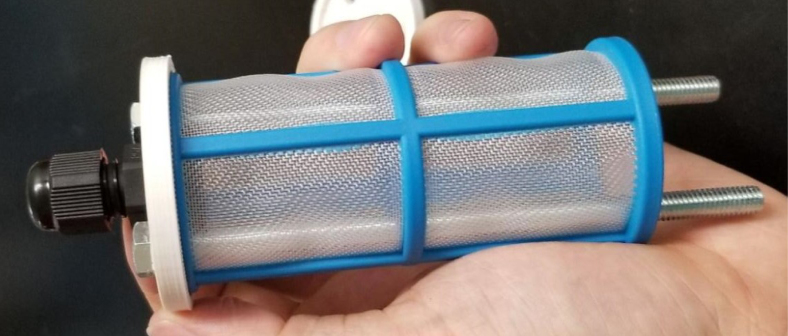
The bolts and filter assembled on lid.

**Fig. 134:**
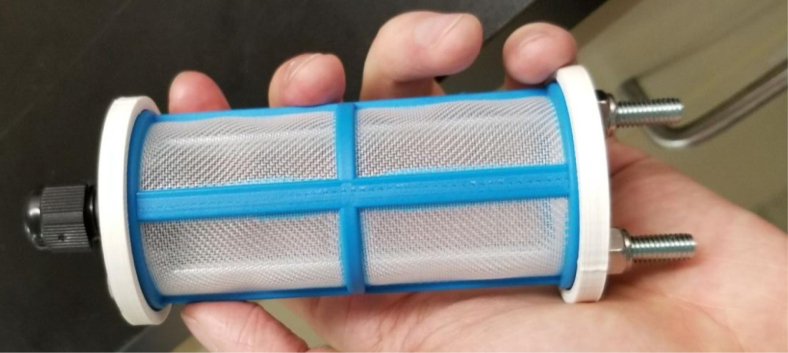
The final pre-filter sub-assembly.

1. Gather the parts shown below.
2. Attach the PG7 Gland to the 3D Printed lid with the hole in the center.
3. Slide the bolts through the other two holes and slide the filter over the bolts.
4. Slide the other 3D Printed Cap onto the bolts and secure them with the Nylon Locking Nuts (see Fig. 133, Fig. 134).

### 5.8 Wiring

This section covers the wiring, crimping, and other electronics assembly tasks that need to be completed for the eDNA Sampler (see Fig. 135, Fig. 136, Fig. 137, Fig. 138, Fig. 139, Fig. 140, Fig. 141, Fig. 142, Fig. 143).

#### 5.8.1 Wiring the sampling valves (positive (red) wires)

1. Bunch up the Pump, Air, and Purge Valve Wires so that they are out of the way.
2. Cut the JST Connectors from all of the sampler valves and separate the Red and Black Wires.
3. Strip the ends of the wires and power each valve to make sure that they are functional.
  We recommend that you use the Multimeter clips to provide power to the valves.
4. Move the black wires away from the extrusions and the red wires toward the extrusions.
  We recommend temporarily binding the black wires together so that they are out of the way.
5. Use a sharpie or tape and mark the middle of a set of 6 valves (3 valves on either side of the mark.
  **Note:** The blue lines denote the center marks in question. There are 4 sets of 6 valves, 2 sets on either side of the sampler.
6. Starting from the rear of the sampler, bundle up the red wires in sections of three valves.
7. Line up the bundle of wires with the nearest center mark from 2 steps ago.
  Be sure to cut the wires a little long so that they are easier to work with Strip the ends of the wires.
8. Repeat this step until every set of three wires is cut and stripped.
9. Cut a length of 22 AWG 2 color wires that is roughly as long as the sampler.
  Separate the 2 wires and strip the ends.
10. Take one blue solder seal connector (SSC) and two sets of three wires cut a few steps earlier.
  Insert two of the three wire bundles and the 22 AWG wires into the blue SSC.
  Make sure all the stripped ends meet where the solder band is.
11. Using the heat gun at 275°F and heat/melt the blue SSC.
  While heating the SSC, move the heat gun around so that every part gets heated.
  It is recommended to heat the glue first and then the solder so that the solder does not escape the SSC.
12. Place the 22 AWG wire onto the frame and mark the wire where the second center mark is.
  Strip the wire where the mark is.
13. Repeat the previous three steps for the next set of wires.
14. Repeat for the other side of the sampler.
  Be mindful as the other side will be a mirrored version of what was just done.
  Always make sure that the 22 Wire goes towards the electronics box/the front of the sampler.
15. Retest all of the valves by powering the combined red wires and sequentially powering/testing the black wires (see Fig. 144, Fig. 145, Fig. 146, Fig. 147, Fig. 148, Fig. 149, Fig. 150, Fig. 151, Fig. 152, Fig. 153, Fig. 154, Fig. 155, Fig. 156, Fig. 157, Fig. 158).

**Fig. 135:**
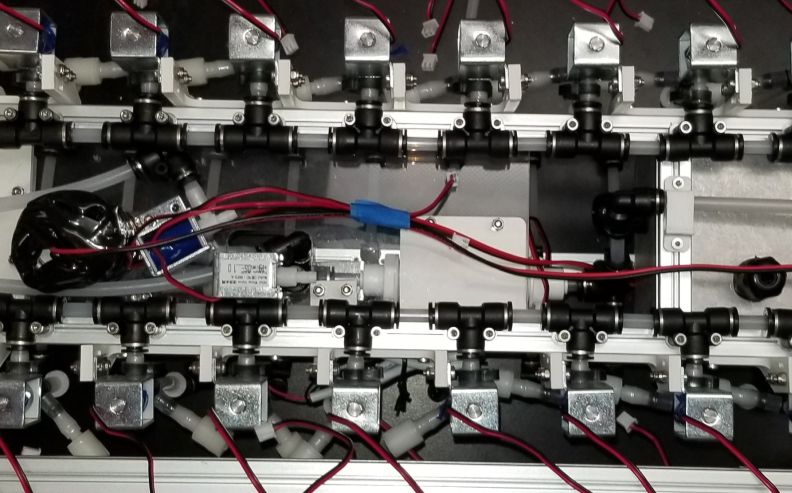
The pump, air, and purge valve wires separated.

**Fig. 136:**
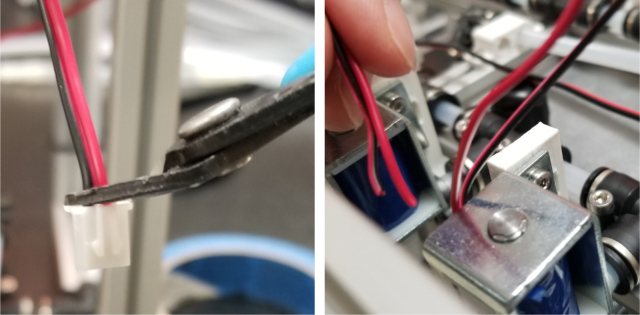
The JST connectors removed from all valve wires.

**Fig. 137:**
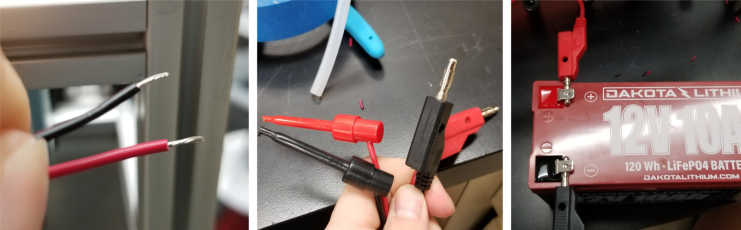
The stripped wires powered by the battery and alligator clips.

**Fig. 138:**
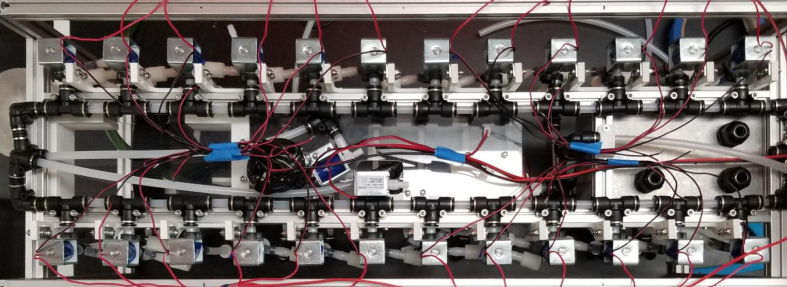
The valve ground and power wires separated.

**Fig. 139:**
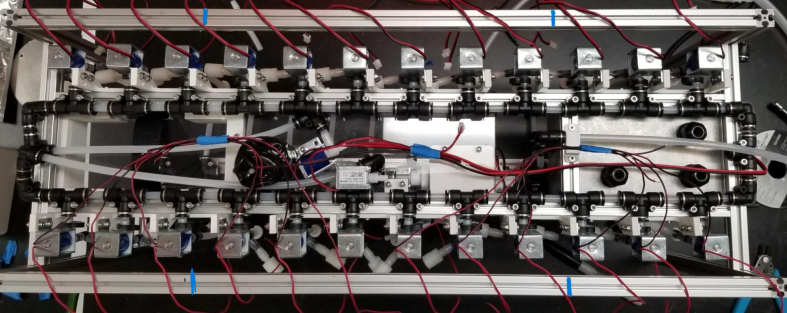
The sampler valve power wires grouped into sets of 6.

**Fig. 140:**
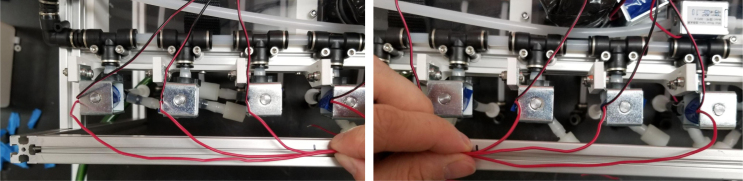
Sets of three power wires bundled.

**Fig. 141:**
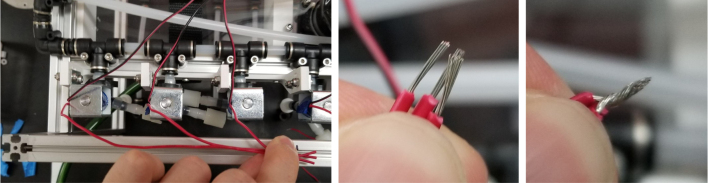
The bundle of wires stripped and connected in groups of 3.

**Fig. 142:**
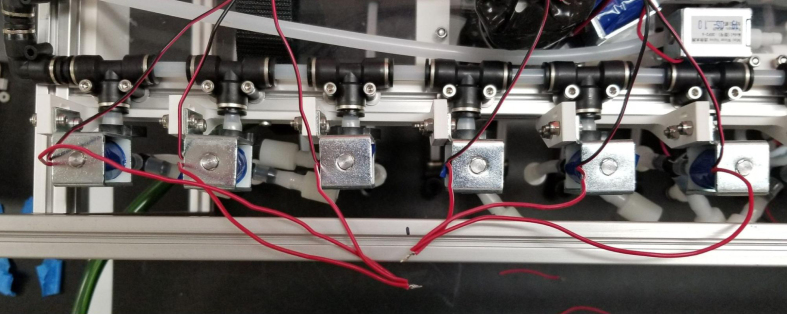
2 sets of bundled wires aligned on the marked frame.

**Fig. 143:**
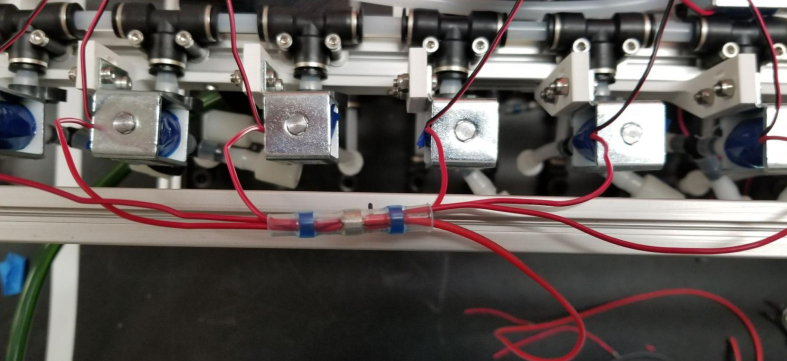
The 6 wires per mark inserted into a solder seal.

**Fig. 144:**
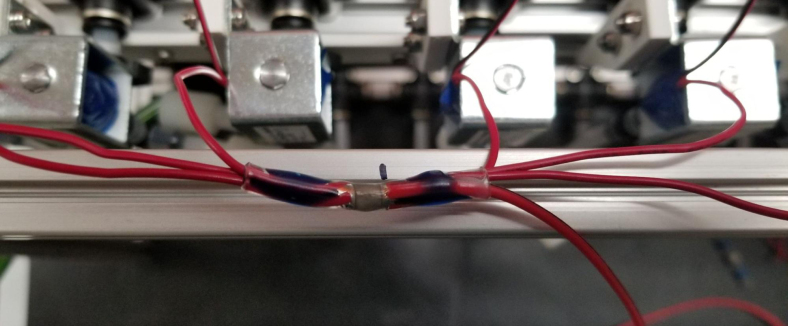
A soldered set of 6 wires.

**Fig. 145:**
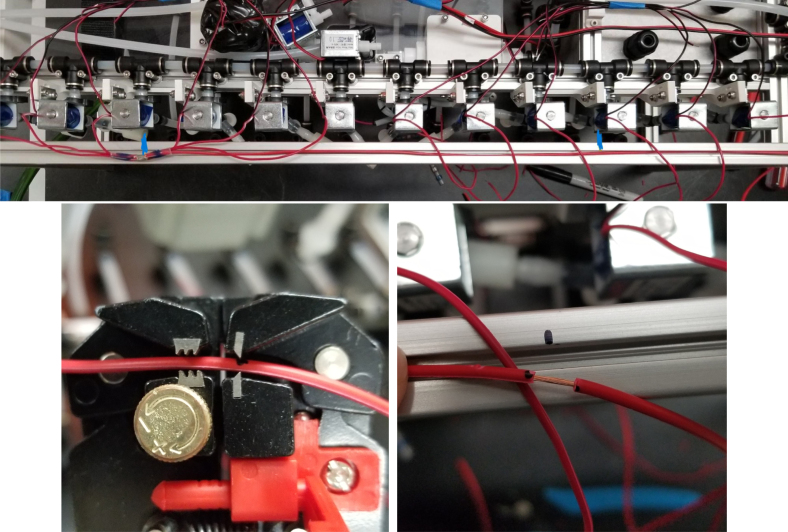
The wire stripped at the center mark.

**Fig. 146:**
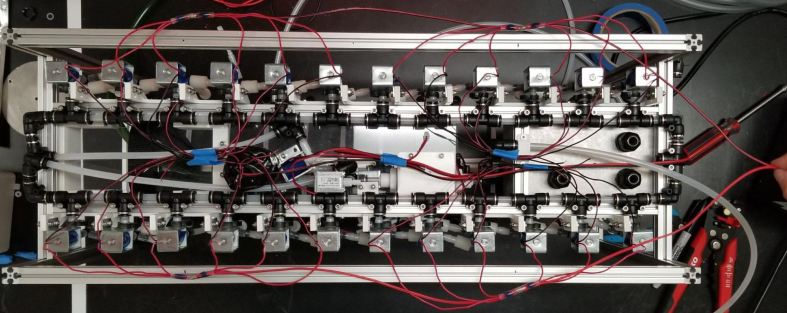
The sampler with all wires connected via solder seals.

**Fig. 147:**
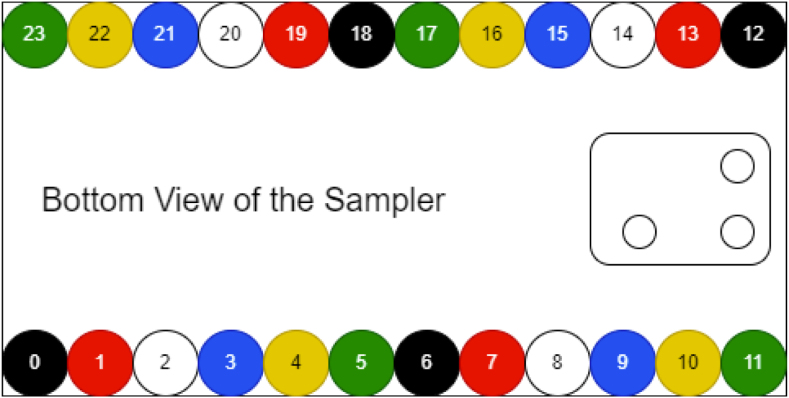
All sampler solenoid valves with color codes.

**Fig. 148:**
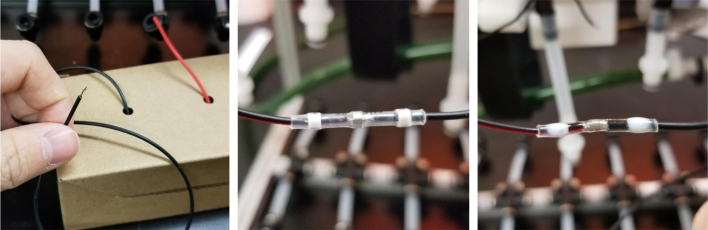
The color wires stripped and connected via solder seals.

**Fig. 149:**
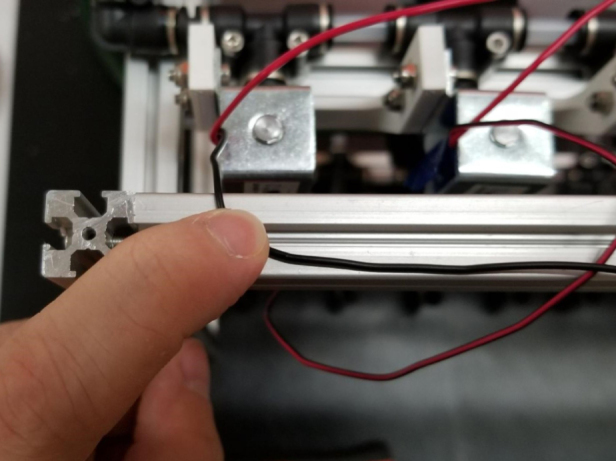
Wire at right angle.

**Fig. 150:**
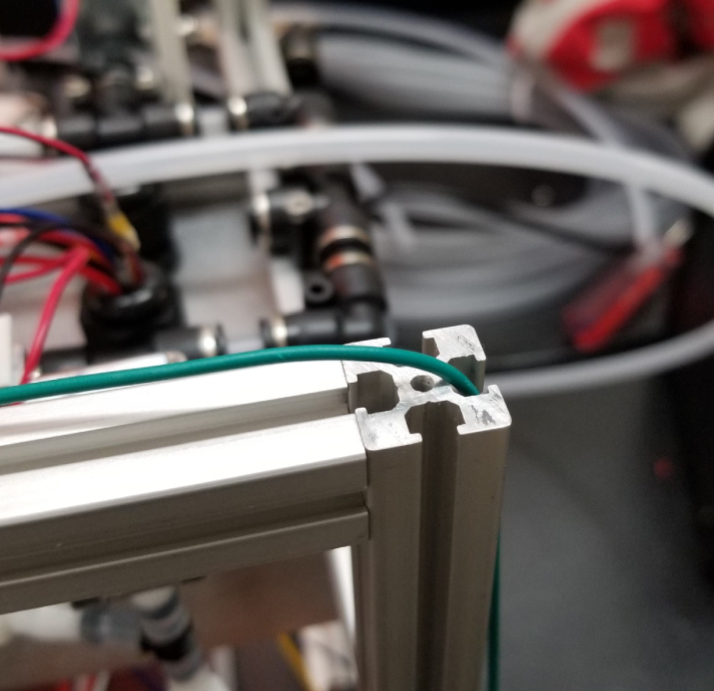
Wire hooked into extrusion.

**Fig. 151:**
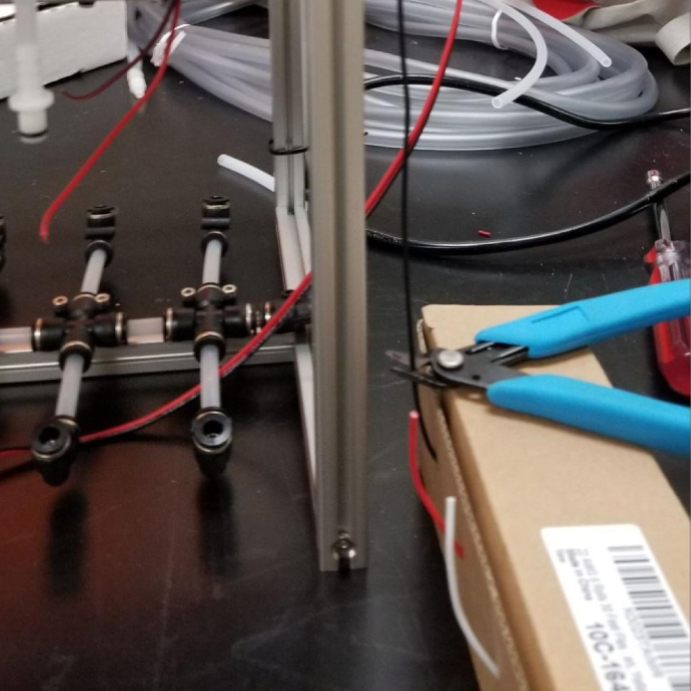
Wire cut from box of wires.

**Fig. 152:**
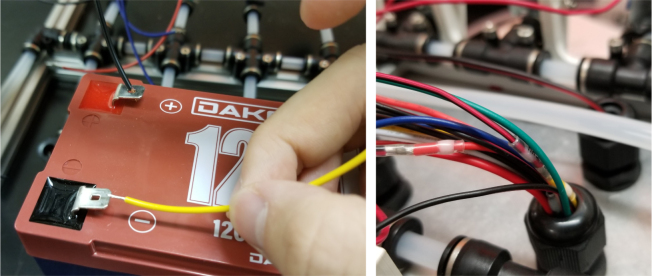
The wires tested and inserted into cable gland.

**Fig. 153:**
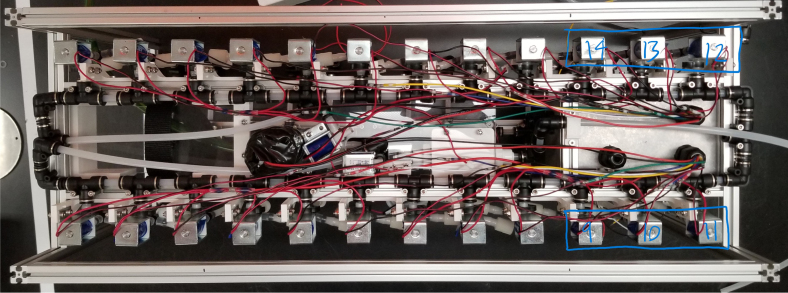
The fully wired valves for sampler.

**Fig. 154:**
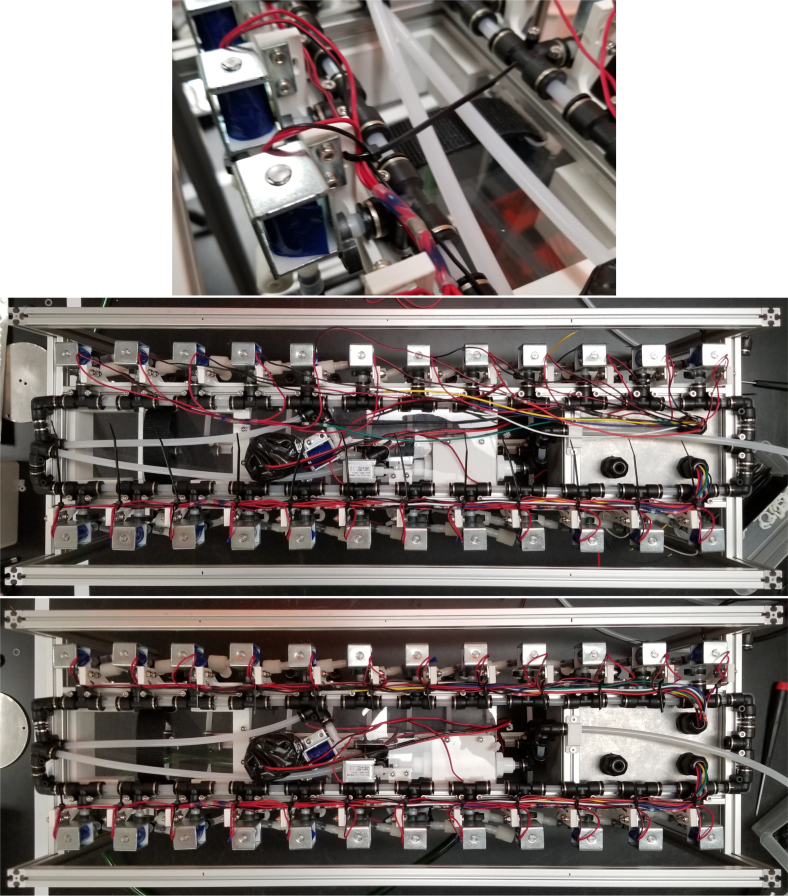
The wires zip tied for managing space.

**Fig. 155:**
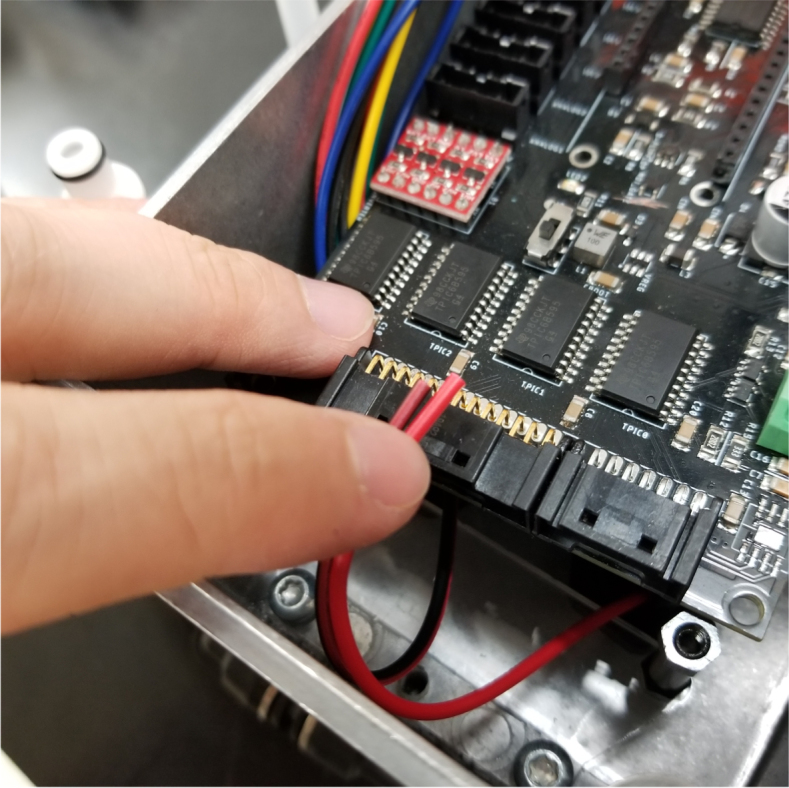
The wires aligned on PCB connectors.

**Fig. 156:**
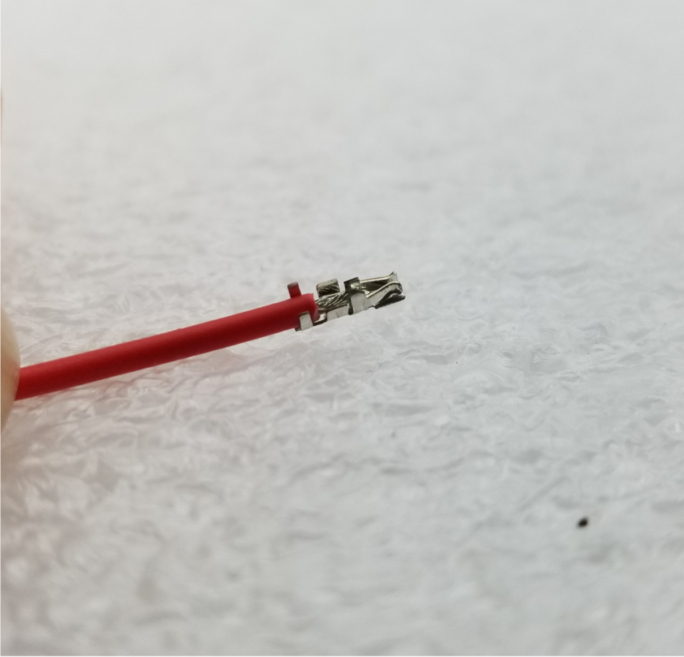
The wire with crimp attached.

**Fig. 157:**
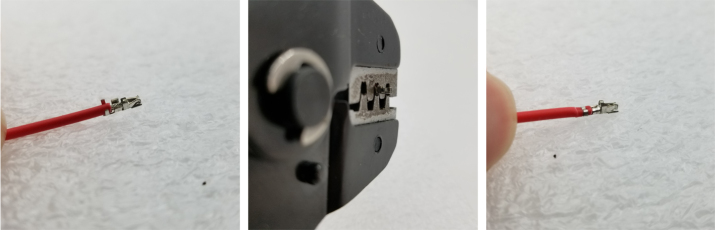
The crimp secured via crimping tool.

**Fig. 158:**
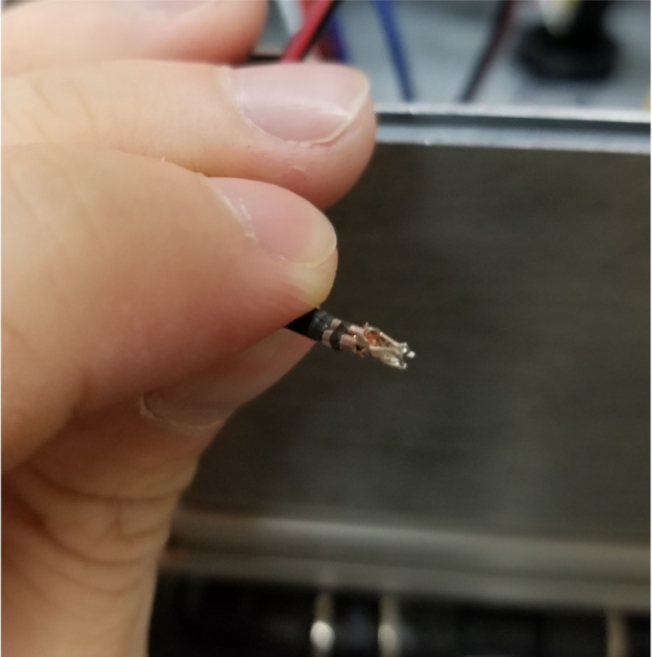
A flattened crimp.

#### 5.8.2 Wiring the sampling valves (negative (black) wires)

1. To make the wiring easier, we will be color-coding the black wires.
  The following image shows the valve number and what color wire will be used for each valve.
2. Strip the end of the colored wire and connect it with the valve wires using the White Solder Seal Connectors (SSC).
3. Once the SSC has cooled, pull the wire box to the front end of the sampler.
  Hold the wire so that it makes a right angle at the valve (First Image).
4. See Fig. 149.
5. Hook the end of the wire with the extrusion (Second Image).
6. Cut the Wire near the box of wires (Third Image)
7. Test the valve to make sure that the electrical connections are good.
  If they are, put the wire through the cable gland.
8. Repeat the previous three steps for all of the valves except for valves 9–14.
  Since these wires are so close to the electronics box, they may need to be cut shorter.
  Cut the black wires for valves 9–14 about half of the length and then repeat the previous steps.
9. Once all the wires have been color-coded and extended, use 4-inch zip ties to cable manage the wires. This is to make them look neat as well as hold the wires in place. There are Zip Tie points on the 3D Printed Valve Hangers.

#### 5.8.3 How to crimp a wire (general)

**Fig. 159:**
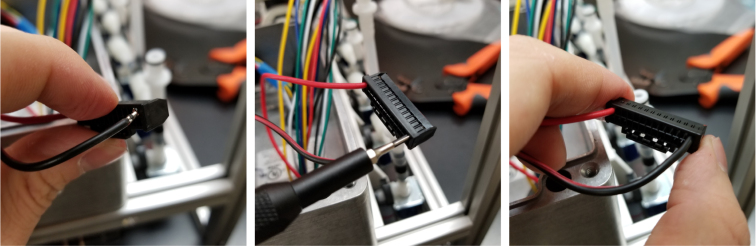
The crimped wire inserted into connector.

1. Use an electronics PCB to approximate where the Valve Connectors reside and cut the wires a little long.
  Use the wire strippers to strip the wire.
2. Take a crimp and the wire you wish to crimp and line them up like in the image below.
  Make sure the insulation lines up with the rear set of “wings”.
  Cut the exposed part of the wire so that the end barely goes past the middle set of “wings”.
  If you used the red backstop on the auto-stripper (in its nearest position), this is about half the length of the exposed wire.
3. Once the exposed wire is cut to length, place the wire into the crimp. The insulation should hold the crimp in place long enough to use the auto-strippers.
  If not, place the crimp into the crimping tool, tighten the grip, and then slide the wire into the crimp. It can be hard to see if the wire is in the right spot, so practice this on wire scraps and get a feel for doing it.
4. Once the wire is crimped, the crimped portion of the wire will be slightly wider than the crimp should be. Use a set of Needle Nose Pliers to compress the wide part of the crimp so that it can be put into the connector.
  **Note:** If the front set of “wings” is crimped by accident, then the crimp needs to be removed and the wire re-crimped.
5. Place the now-crimped wire into the corresponding spot of the connector.
  If it does not go in, check if any part of the crimp is too wide or too tall.
  If the crimp looks fine, take a thin pin (like a paper clip or a SIM card tool) to push the crimp in. Be sure to push on the crimp itself and not the wire.
  Make sure that the front set of “wings” pushes past the plastic flap of the connector (see Fig. 159).

#### 5.8.4 Crimping the wires

Since there are six colors of wire and 24 valves, there are four valves for every color. To determine which valve is which, only two wires (of the same color) need to be tested at any given time. This is because the valves are split 12 per side meaning each side has two wires of the same color. Use a 12 V battery to power the wires to determine which wire corresponds with which valve.

Valves 0–11 reside on the right side of the sample.

**Fig. 160:**
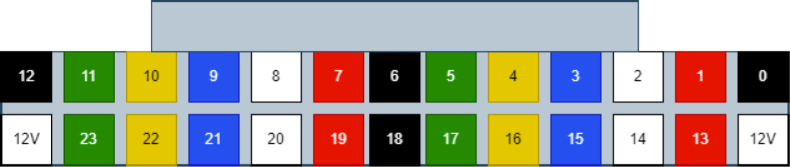
The connector mapped for each wire.

**Fig. 161:**
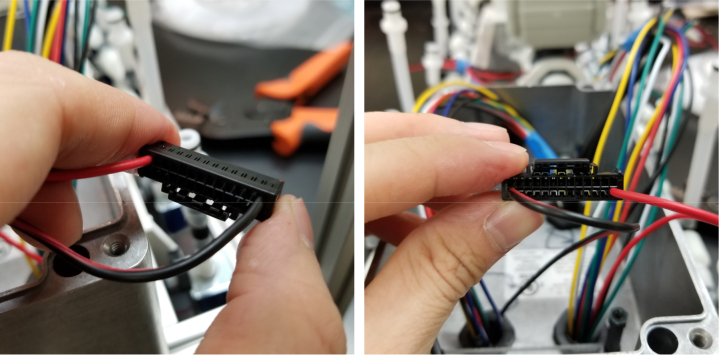
The fist two wires inserted into the connector.

Valves 12–24 reside on the left side of the sampler (see Fig. 160, Fig. 161).

**Fig. 162:**
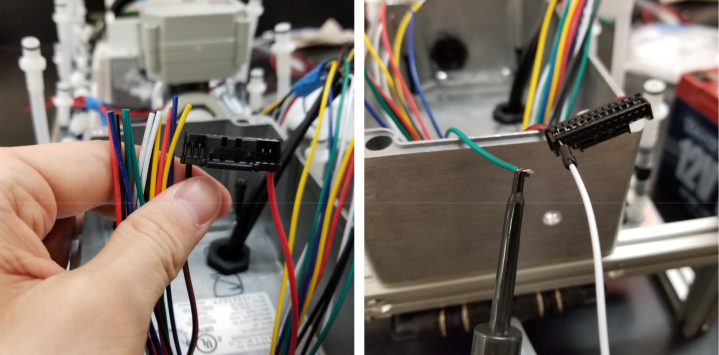
The crimped wires inserted into connector.

**Fig. 163:**
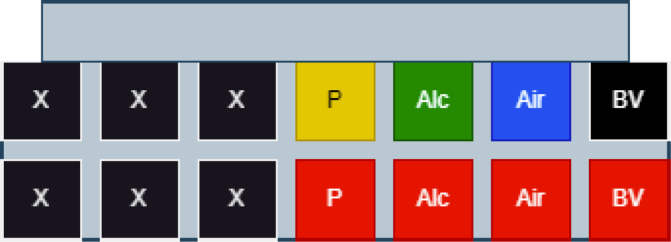
The central component connector mapped for each wire.

**Fig. 164:**
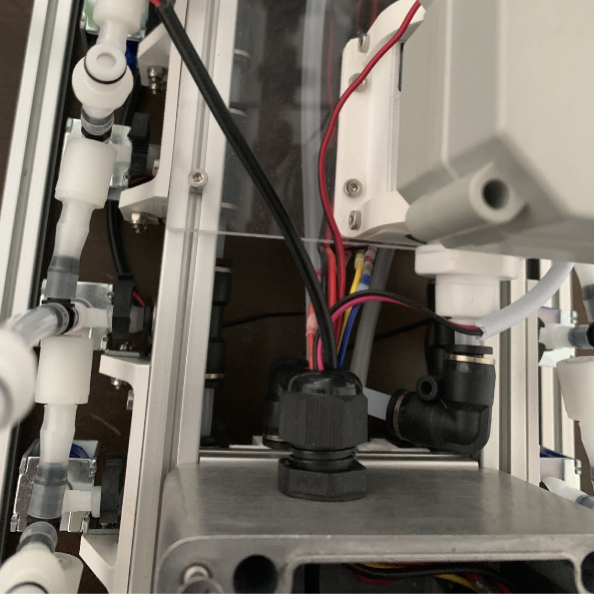
The central component wires inserted into cable gland.

**Fig. 165:**
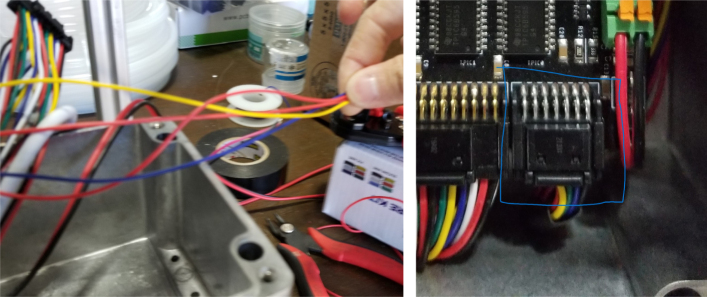
The wires cut to length.

**Fig. 166:**
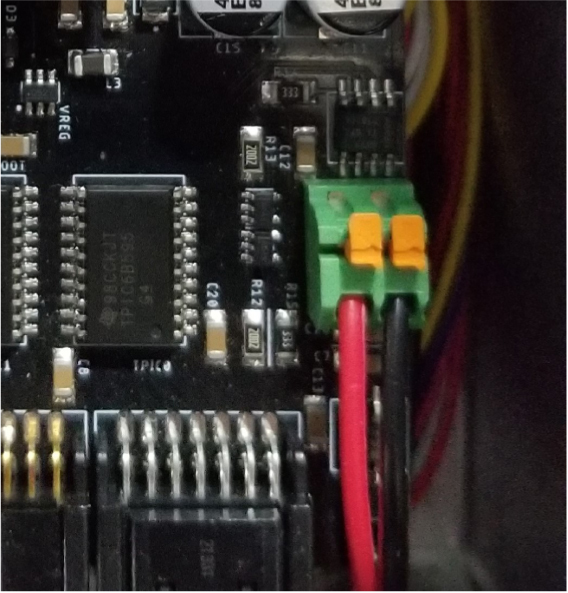
The stripped motor wires inserted into PCB connector.

**Fig. 167:**
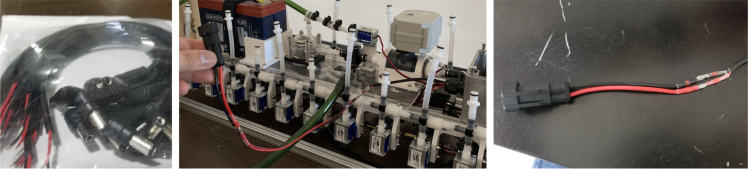
The waterproof connectors connected to battery.

1. Start by crimping the two 12 V (positive (red) wires) and inserting them into the connector.
2. Take a set of the GND (Color coded) wires and begin crimping them. We recommend crimping the GND wires for valves 12–23 first, starting from the rear of the sampler and moving towards the front (23 -> 12).
  Since two valves share the same color, use a male-to-male wire to connect to the 12 V wire connected to the set of wires being worked on. Connect the 12 V and GND Wire to the battery to figure out which valve the wire is connected to.
3. Repeat with the next side of the valves, again starting from the rear of the sampler and going forward. Use the same checking technique to figure out which wires go to which valve.
4. Repeat until all the wires have been attached to the connector (see Fig. 162, Fig. 163, Fig. 164, Fig. 165, Fig. 166, Fig. 167).

#### 5.8.5 Wiring the central components

**Fig. 168:**
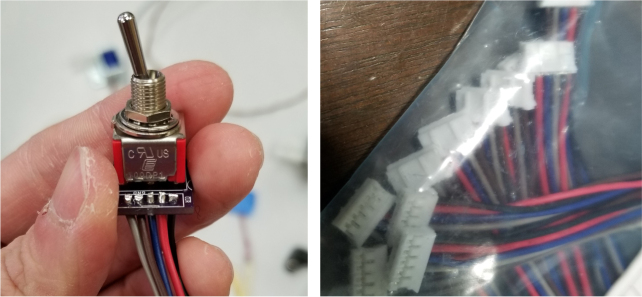
The DPDT switch and PCB connector.

**Fig. 169:**
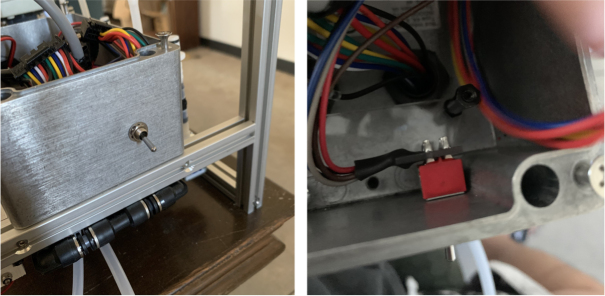
The switch mounted to electronics box.

**Fig. 170:**
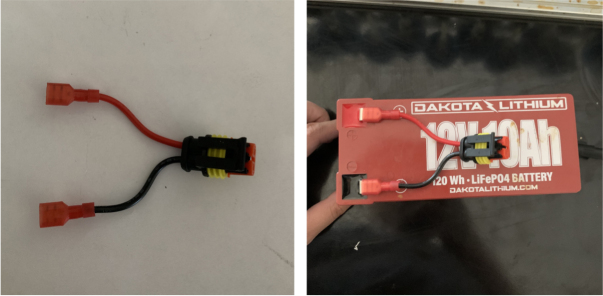
The male 2 pin connector attached to battery.

**Fig. 171:**
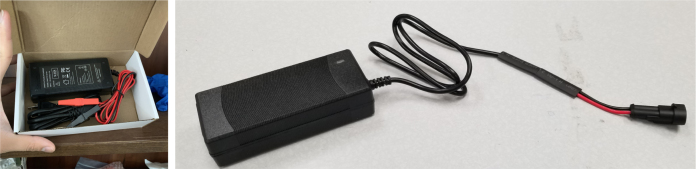
The Battery charger and final modifications.

**Fig. 172:**
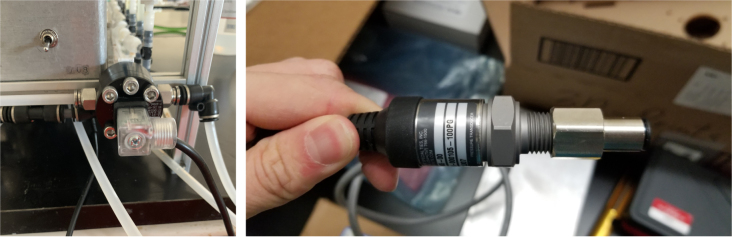
The pressure sensor with required fittings.

**Fig. 173:**
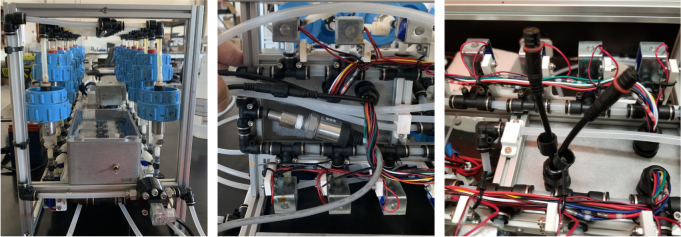
The flow meter and pressure sensor mounted in their respective locations.

**Fig. 174:**
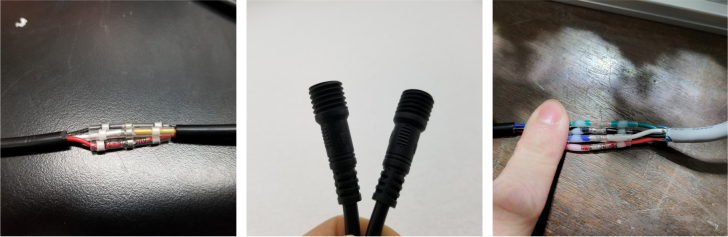
The sensor wires solder sealed to waterproof connectors.

1. Extend the wires of the three solenoid valves and the Ball Valve, so that they go past the electronics box about 3in.
  Use the Red Wires to extend the positive (red) wires of the solenoids. Color the following color code for the GND (black) wires.
  • Green for the Preserve Valve
  • Yellow for the Purge Valve
  • Blue for the Air Valve
  • Black for the Ball Valve
2. Push the wires of the solenoids, ball valve, and pump through the rear cable gland.
3. Like with the Sampling Valves, Cut all the wires to length, cutting them a little longer to be safe.
  The valves will go into the 14-pin Hirose connector.
4. Like with the Sampling Valves, crimp the valve wires with the DF-51K 22 AWG Crimps.
  The image below shows where each cable needs to go.
5. The motor wires do not need to be crimped, only stripped.
  The orange tabs of the connector are spring-based and need to be depressed for the wire to slide in. Once the wire is in, let go of the orange tab, the springs should hold the wire in place.
  The red wire goes into the **left** side of the connector.
  The black wire goes into the **right** side of the connector.
6. After that, take a right-angled barrel plug and push it through the rear cable gland from the inside of the electronics Box.
  Use the 22AWG Wire to extend the barrel plug wires toward the battery.
  Solder a female 2-pin Waterproof connector to the extended power wire.
7. Solder the DPDT Switch to the custom switch PCB.
  Solder the 5-pin JST wire to the switch PCB. The black wire will solder to the hole with the square pad.
8. Mount the Switch to the electronics box and plug the connector into the main control board.
9. Crimp the Male 2-pin waterproof connector with a red spade connector and plug it into the battery
10. Cut off the Alligator clip ends off of the battery charger and solder a female 2-pin waterproof connector to it and cover with heat shrink (see Fig. 168, Fig. 169, Fig. 170, Fig. 171, Fig. 172, Fig. 173, Fig. 174).

#### 5.8.6 Wiring the sensors

**Fig. 175:**
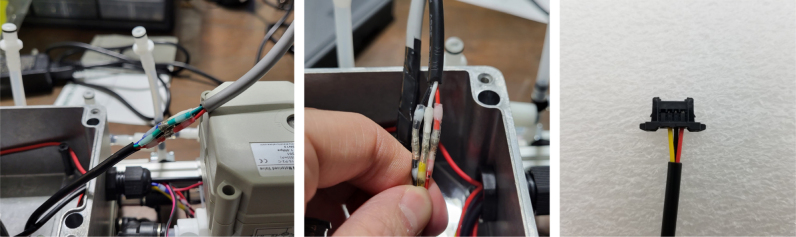
The sensor wires crimped and inserted into connectors.

1. Add the necessary connectors to the Flow Meter and Pressure Sensor.
  Be sure to use Teflon Tape for the Pressure Sensor Fittings.
2. Mount the Flow Meter and Pressure Sensor in their respective locations.
  Place the cap side of the 3-pin and 4-pin waterproof connectors into the sensor Cable Glang (Bottom rear).
3. Route the sensor wires toward the waterproof connectors.
  Once routed, cut the sensor wires near the connectors.
  Solder the Threaded side of the waterproof connectors to the sensors wire.
  Be sure to match the colors when possible and for the 4-pin connector match.
4. Use the sensor wire cutoffs to extend the sensor connectors inside the electronic box.
  Crimp the sensor wires and insert them into the 4-pin Hirose connector.
  **Note:** The Hirose connector is directional and the Flow Meter and Pressure sensor have different pin-outs. The pin-out of the connector is marked on the silkscreen of the Main control board (see Fig. 175).

#### 5.8.7 Final PCB connections

1. Attach the Sensors connectors to the PCB connectors indicated in the eda schematic.
2. Attach the valve connectors to the PCB connectors indicated in the eda schematic.
3. Attach the Arduino feather Micro-controller to the PCB connectors indicated in the eda schematic.
4. inset a micro SD card into the PCB’s micro sd card connector indicated in the eda schematic.
5. Attach the PCB power connector to the PCB connectors indicated in the eda schematic.

## 6 Operation instructions

### 6.1 User interface setup & browser configuration

The codebase for the user interface (UI) is designed to be compiled into files that are put into the SD card so that the sampler machine can read. As a npm package, Node.js, specifically Node.js 10, and npm are required to run the scripts to compile. Information for installing both is available here. After downloading the codebase repository, it can be installed using the npm CLI commands install and compiled using the build script with the command run-script. The compiled files are created in the /dist subdirectory of the repository.

Note that the sampling machine uses the HTTP protocol, and not HTTPS. This means that depending on your browser, you will have to ensure that it does not fallback to HTTPS when trying to load the UI from the sampler. The WiFi network name and password are set within the configuration.hpp file of the sampler codebase, and the UI is always hosted on the IP 192.168.1.1 on that network.

### 6.2 Sampler code upload

**Fig. 176:**
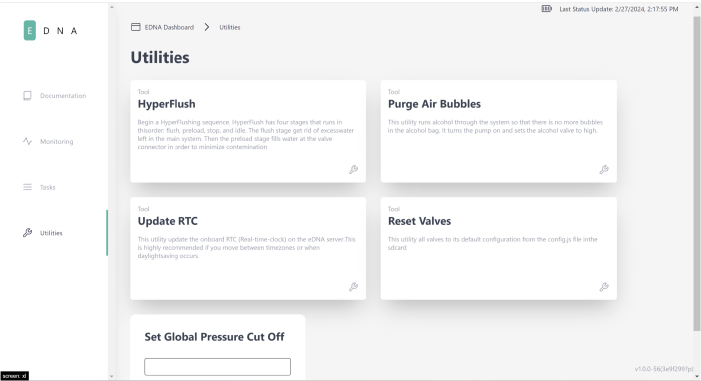
Screenshot of user interface on utilities page.

The codebase for the sampler machine uses PlatformIO, an IDE for Microsoft Visual Studio Code. Downloading the codebase also requires downloading another repository, the framework repository, that is used as a Git submodule in the /lib subdirectory. Information on how to use PlatformIO is available here (see Fig. 176).

### 6.3 Sampler cleaning

To ensure as little contamination as possible, the Hyperflush procedure can be used to clean the sampler. The procedure is available by connecting to the UI under the utilities tab. To clean the sampler, put all inputs and outputs into a container of bleach, and run the Hyperflush procedure completely five times. Afterwards, put the intake line into Ascorbic Acid and run the HyperFlush procedure five times. Finally, put the intake line into a DI water source and all other connections into a disposal system or sink, and again run Hyperflush five times.

### 6.4 Sampler/filter setup

**Fig. 177:**
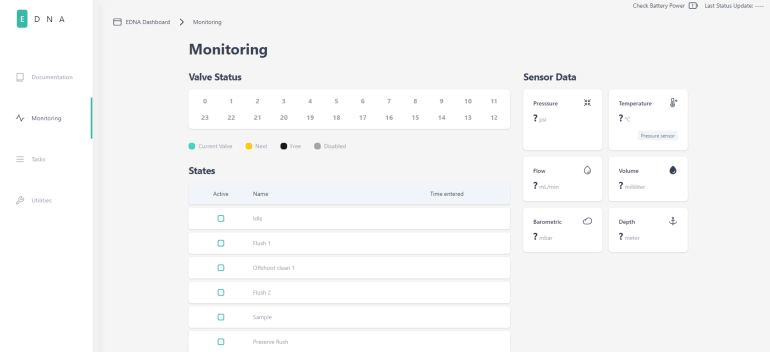
Visual indicator of filter labels.

**Fig. 178:**
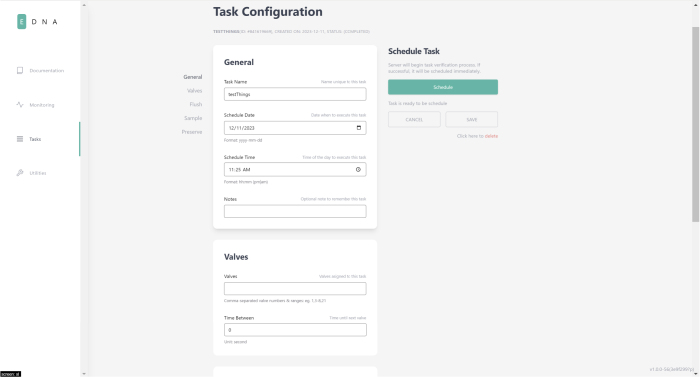
Screenshot of User Interface on Task Page.

Filter housings have a quick-connect plug on the bottom of their assembly, which allows for them to be quickly switched out with other housings as needed. The filter housings themselves are designed to be hand tightened and loosened to access the membrane filter inside. The valve layout that tasks use follows the diagram below: (see Fig. 177, Fig. 178).

### 6.5 Task configuration

**Fig. 179:**
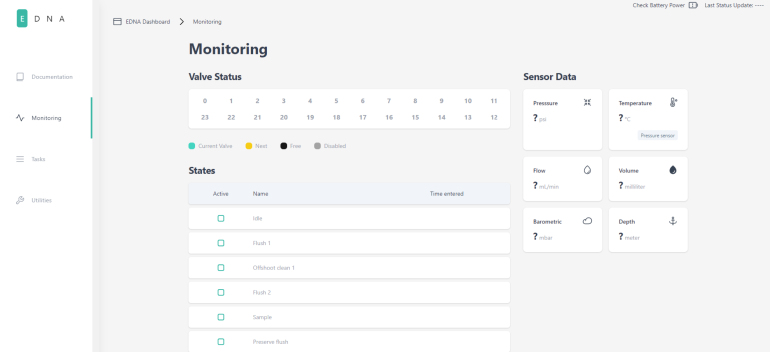
Screenshot of User Interface of a Task Configuration Page.

Scheduling a task is done with the UI by navigating to the tasks tab, and either creating a new task or modifying an old one. Tasks are sorted on the task tab by whether they are scheduled or inactive. Clicking on a task will bring you to the task configuration page, where you can set different parameters for the task. Scheduled tasks need to be unscheduled if they are going to be modified, otherwise they will be executed on the scheduled time. If a task has any parameters change, they must be saved before scheduled (see Fig. 179).

Tasks can sample with multiple filters, with the ability to set the time between the ending of sampling one to the start of another sampling. Additionally, some states of the sampling procedure have a variable time that can be set by the user: flush, sample, dry, and preserve states. The sample state is unique in that the state will be considered complete depending on the time in the state, the volume of water sampled, and the maximum pressure reached while sampling. All three parameters can be set by the user depending on their use case.

## 7 Validation and characterization

The PolyWAG envirSampler was tested by the Openly Published Environmental Sensing Lab at Oregon State University. The primary use case of the PolyWAG eDNA sampler is for use in water sampling to capture existing trace biological information in the form of DNA by means of filtration. In investigating this, the sampler was evaluated not only on its ability to collect and store DNA, but also its ability to prevent cross-contamination between samples, accurately measure filtered volume, its effectiveness in various environments, and the energy needed to operate. It was characterized by a blend of isolated in-lab testing procedures. To assess the capabilities of the sampler, the following evaluations were conducted: to establish the viability of field deployment at running water sources in Alaska.

### 7.1 Cross-contamination testing

As established earlier the sampler must be able to not only collect and store eDNA, but must also be able to prevent cross-contamination between samples. To establish and validate this aspect, the sampler was subjected to a lengthy cross-contamination study for detecting residual DNA between samples. Prior to sampling, a cleaning and sterilization process was conducted across the sampler to eliminate any sources of contamination from previous testing. After running the sampling procedure, the results were evaluated by means of Polymerase-chain reaction (PCR).

The sampler’s cleaning procedure uses three cleaning cycles that make use of the sampler’s HyperFlush utility to sterilize both the filter housings and the sampler’s hydraulic lines. The hyperflush utility flushes each valve of the system sequentially with any bulk liquid connected to the input line. Each cleaning cycle uses the hyperflush utility three times with liquids in the following order: DI water, 6% bleach, 5% ascorbic acid. The three cleaning cycles are followed by three subsequent hyperflush cycles of DI water to purge all residual chemicals. Once the cleaning procedure was completed filters were loaded to prepare for the sampling procedure.

The basis for this analysis and observations was the use of Alaskan Sockeye Salmon DNA. The Salmon DNA was prepared by releasing it into liquid solution. This was accomplished by placing one filet of salmon ( 500 g) in an eight Gal container of DI Water for 24 h. During this time, the salmon filet broke apart and biological material including DNA diffused into the bulk water. After 24 h, the remaining salmon mass was removed to ensure constant salmon DNA concentration across trials.

The final two samples of the cycle are the main samples of interest and contain DI water to allow the sampler’s cross-contamination to be gauged. Because the samples were conducted with shared hydraulic lines, if cross-contamination existed it would appear in subsequent samples. Separate inlet lines were used for DI water and fish water to isolate the source of cross-contamination to the sampler itself, with the lines being switched manually between samples. The data collected during this study consisted of four trials performed using the following task settings on the eDNA UI: 1000 mL max sample volume, four-minute max sample time, 10-second preservative flush, and 24-minute flush time ( 5 Liters). The samples were collected by using filtration across Tisch Cellulose Nitrate (CN) Membrane filters with a 47 mm diameter and 5.0μm pore size. After sampling, the samples were individually packaged in sample containers and sent for analysis.

Analysis of the samples was conducted using a standard qPCR technique to amplify and quantify DNA by the Levi lab in the Department of Fisheries and Wildlife at Oregon State University []. During which the sample filters were first lysed, and then amplified using an Alaskan Sockeye Salmon specific primer [7]. The samples then proceeded to a qPCR technique combining immunofluorescence with PCR to quantify the DNA amplification. During each PCR cycle, the DNA was amplified and the fluorescence of the sample was measured for intensity. This continued until the fluorescence reached the machine-specific detection threshold. The number of cycles needed to reach this threshold is known as the CT or Cycle Threshold [8]. Using this methodology, a cycle threshold between 0–40 indicated the presence of Salmon DNA, while a CT score greater than 40 indicated no Salmon DNA was detected. Using these processes 13 samples were taken using the sampler. This included one Negative control, and four sample trials, as well as lab controls (see Table 2).

The four trials conducted all yielded the same conclusion, there was no-cross contamination between samples. The CT score of the initial DI water sample (Negative Control) was not detectable ensuring the quality of the sterilization process and DI water. The CT scores of the samples containing fish DNA (Positive Control) contained positive signals with CT scores requiring 35 cycles to reach the target illuminance. The samples of interest following the fish DNA contained no detectable fish DNA indicating no cross-contamination. Based on these results the sampler provides evidence that there is no cross-contamination between samples. However, it does so only at DNA concentrations that require less than 33.9 PCR cycles to reach the threshold. When comparing the test controls to the lab controls there is one difference noticed. The Lab positive control contains fish DNA at a much higher concentration: 20 CT vs 33.9–35.6 CT in the experiment. This indicates that the lab’s positive DNA sample is 1015x more concentrated with DNA than the fish water sample collected. There are two possibilities explaining this: The fish water did not contain the maximum Sockeye salmon DNA, or that the sampler is not collecting the same concentration of DNA in the water as traditional bag sampling.

**Table 2:** Cross-Contamination qPCR results in no-detectable DNA in subsequent samples.

Sample name	CT	Results
Test Negative Control	Undetermined	**Negative**
Test Negative Control	Undetermined	**Negative**
Lab Positive Control	20.20038033	**Positive**
Lab Positive Control	20.4094305	**Positive**
Lab Negative Control	Undetermined	**Negative**
Lab Negative Control	Undetermined	**Negative**
Test Negative Control	Undetermined	**Negative**

Positive Control	34.42589188	**Positive**
Positive Control	35.62890244	**Positive**
Trial 1: Sample 1	Undetermined	**Negative**
Trial 1: Sample 1	Undetermined	**Negative**
Trial 1: Sample 2	Undetermined	**Negative**
Trial 1: Sample 2	Undetermined	**Negative**

Positive Control	36.21839905	**Positive**
Positive Control	36.1700592	**Positive**
Trial 2: Sample 1	Undetermined	**Negative**
Trial 2: Sample 1	Undetermined	**Negative**
Trial 2: Sample 2	Undetermined	**Negative**
Trial 2: Sample 2	Undetermined	**Negative**

Positive Control	33.92808533	**Positive**
Positive Control	34.61808395	**Positive**
Trial 3: Sample 1	Undetermined	**Negative**
Trial 3: Sample 1	Undetermined	**Negative**
Trial 3: Sample 2	Undetermined	**Negative**
Trial 3: Sample 2	Undetermined	**Negative**

#### 7.1.1 Turbidity

The rate at which a filter clogs is a function of particle size and count. Specifically, the eDNA sampler’s filters are clogging from suspended solids at various sizes and concentrations in the samples. This is intentional as the purpose of the sampler is to capture the DNA that binds to these particles. At the same time, eDNA collection typically has a threshold of filtered volume required for analysis [9]. This indicates that not only is the collection of eDNA important, but also the volume of water passing through the filters. For this reason, the characterization in the laboratory was idealized without the variations in suspended solids, and therefore must be validated using non ideal conditions.

To ensure the effective use of the sampler in non-ideal conditions, the sampler was analyzed by means of a flow analysis across a range of turbidity levels. The purpose of this test is to ensure adequate flow through the filter before it clogs, and to gauge this clogging as a function of water quality by means of turbidity. Turbidity is an optical measurement indicating the clarity of a sample [10]. Therefore determining the turbidity of a sample is not a direct representation of the particulates in the water; however, it is commonly used to gauge the water quality in the field where suspended solid information is not readily available. For our eDNA collection purposes, a minimum of 500 mL of filtered volume is required to accurately analyze the DNA concentration of the source. This testing serves to provide the expected filtered volume for various turbidity levels and therefore the environments where the sampler can operate. For this analysis, a 75.0μm pre-filter and 5.0μm cellulose nitrate filters are used.

**Fig. 180:**
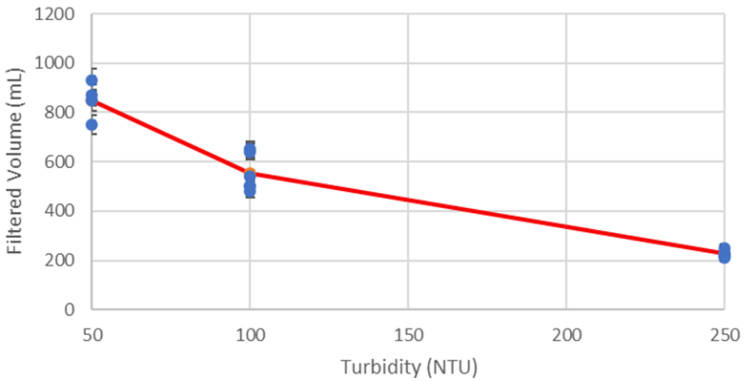
As turbidity increases, the filtered volume through the 5.0μm filter decreases due to increased filter clogging.

Based on water turbidity data for Southeast Alaskan rivers, the turbidity of the water ranges from 0–200 NTU [11]. To reproduce this range, a Hach 4000 NTU formazin based turbidity standard was diluted to produce the following NTU standards: 50, 100, 250 NTU. These standards were then mixed via a stir bar. The inlet pre-filter was suspended two inches from the bottom of the beaker while sampling. Sampling was conducted and stopped once the flow rate dropped below 60 mL/min. This is due to the lower limit of the inline flow meter, a drop in flow rate below this limit indicates a filter clogging and a restriction of flow below detectable levels. To gauge the effectiveness of the sampler at different turbidity levels the values are compared to the 500 mL minimum filtered volume requirement. Greater than 500 mL of filtered volume indicates a successful sampler run at that water quality (see Fig. 180).

The data from these trials suggest that the sampler successfully operates in the range of 30–80 NTU. The data indicates the sampler can run in water qualities up to 100 NTU before samples under 500 mL are observed. Subsequent testing at 250 NTU indicates for lower-quality water streams, the sampler’s function decreases substantially, only filtering 200–250 mL per sample. This indicates that the sampler’s current configuration is able to support water qualities below 100 NTU. For environments with turbidity greater than 100 NTU, changes to the sampler’s configuration will be required to reach a higher target volume.

#### 7.1.2 Flow accuracy testing

In manual eDNA collection, the baseline requirements are captured eDNA and accurate recording of the filtered volume. When designing the automated sampler these requirements translate. Without an accurate measurement of volume, it is impossible to determine the concentration of the captured eDNA. The mechanism for determining the filtered volume is controlled by the inline flow sensor. A turbine in the sensor measures flow by sending electric pulses every time the turbine makes a full rotation. The software interprets this value by adding a constant indicating a fixed volume of water has passed to the total filtered volume for every pulse. To make matters more complicated, the kinematic viscosity of water varies with temperature, meaning the force applied by a given volume changes as the temperature changes. For this reason, the volume constant must also be optimized not only for flow but also temperature.

**Fig. 181:**
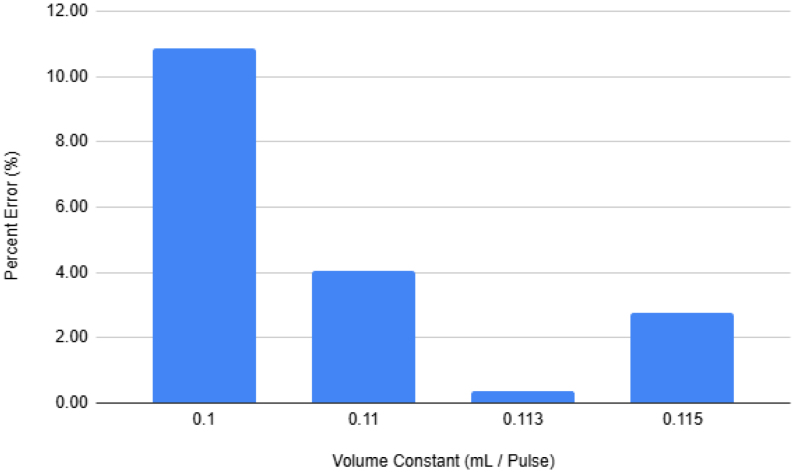
The pulse volume constant indicating the highest precision is 0.113 mL/pulse.

To ensure the sampler the flowmeter provides accurate flow readings for the upcoming Alaskan trials, the sampler was calibrated to reach accurate flow readings between 3-5’C. For accurate eDNA measurements the measured filtered volume must be within 10% of the true filtered volume. Using the 10% value as a threshold, several trials were conducted to determine the optimal volume constant for the flow meter pulses. In these trials, the outlet of the sampler is manually measured with a graduated cylinder and compared to the sampler’s pulse calculated value. The percent error was then calculated between the two quantities. To ensure accuracy across the sampler’s operating range, the filtered volume was measured and compared at 250, 500, and 1000 mL for each volume constant. By averaging these values at each volume constant the average error per volume constant was discovered. Using these values, the volume constant was selected for the lowest percent error (see Fig. 181).

**Fig. 182:**
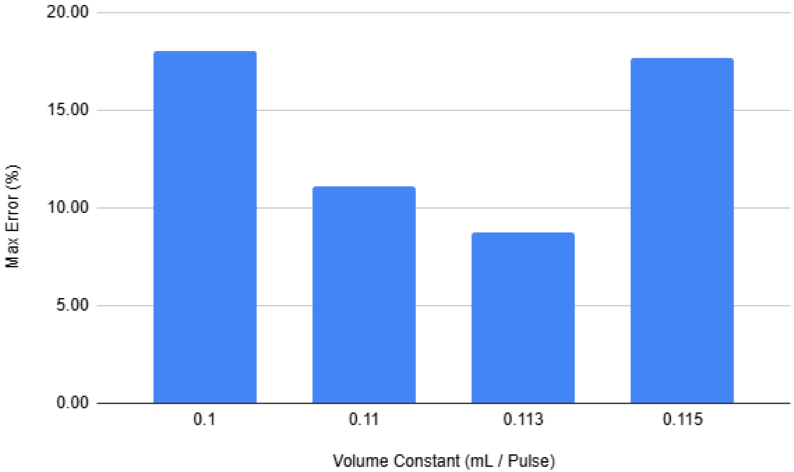
Using the 0.113 mL/pulse volume constant the maximum error falls below the 10% error threshold.

The results determined from this process illustrate the absolute error observed for different volume constants. Therefore the smaller the magnitude indicates offsetting error. In other words, the smaller the bar the more the overestimates and underestimates negate each other. This essentially centers the solution in the error domain. The minimum error indicates an ideal volume constant at or near 0.113; however, this alone does not demonstrate the accuracy of the volume measurement. Because the 10% error is a threshold the max error between trials is a better indicator of the flow meter’s accuracy (see Fig. 182).

When viewing the max magnitude of error, a convex solution approaches minimum error at a volume constant of 0.113 mL/pulse. Because the maximum error is lower than 10%, the sampler satisfies the accurate volume condition Therefore, the sampler is validated to record accurate flow readings when the volume constant is set to 0.113 mL/pulse.

#### 7.1.3 Battery life testing

The power consumption of the sampler is heavily dictated by the current pull of the pump while under load; therefore, to gauge the energy storage needed to power the sampler, a worst case power expenditure model is used. The pump is powered by 12v and variable current draw. The maximum current draw allowed by the pump in our design is 2 A, while the minimum current for the motor is 0.5 A. This means the worst-case energy consumption of the sampler is 24Wh. As described earlier, the normal sample procedure has a maximum runtime of 28.17 min. That runtime includes 24 min of flush time, 0.17 min of preserve time, and a maximum of four minutes of sample time. This indicates that a single sample requires 11.27 Wh and the worst-case scenario energy budget of 270.4 Wh is more than double the design’s 120 Wh battery. However, during real-world operation of the sampler this is not realistic. During the flush states, the flow of the hydraulics is not restricted indicating the pump is under low load. The pump can only be under high load during the 4.17 min of sample and preserve time. This changes the requirements for the energy budget as one sample requires 4.07 Wh and a full 24 sample set would require 97.60 Wh of energy storage. The included 120 Wh battery contains 120 Wh indicating a 23.0% energy surplus under normal operation. Nonetheless, the sampler is designed to be upgradeable and another battery can be slotted in if the end user requires a different procedure.

Battery life also varies with the ambient temperature of the surrounding environment [12]. To gauge the battery life of the sampler in a cold Alaskan environment the battery was placed in a freezer at 0’C. The battery was left for two weeks to simulate the two-week field deployment time in Alaska. After two weeks, the battery line was plugged into the sampler while remaining in the freezer. A full 24-sample procedure was conducted. To ensure normal operating conditions the full 24-sample procedure was used using the default sampling procedure with low turbidity water. Because the battery did not die during this time the sampler was verified to have enough battery life for normal operation of the sampler during a two week field deployment time.

## 8 Conclusion

In our paper, we introduced the PolyWAG eDNA sampler, an automated solution for collecting environmental DNA from aquatic environments. This system addresses the limitations of current eDNA sampling techniques, which are often labor intensive, costly, or limited in capability. Despite its advantages, we acknowledge areas for improvement to enhance its performance further. Our recommendations focus on increasing the sampler’s capacity to handle larger volumes and higher turbidity, integrating an automatic calibration for water viscosity adjustments based on temperature changes, and employing more robust components such as stronger pumps and high-pressure rated solenoid valves. Additionally, in remote areas using ethanol as a preservative is not always viable. Therefore we have also began development integrating self-preserving filters into the design. Furthermore, We propose enhancements like a closed-loop bleach cleaning system for reducing cross-contamination, a pulse damper for pressure stabilization, and improved flow meter accuracy through noise filtering and firmware adjustments. These modifications aim to make the PolyWAG sampler even more effective and reliable for diverse field conditions, paving the way for its broader application in environmental DNA research and monitoring.

## 9 Ethics statements

The work does not use any human or animal subjects.

## Funding

This work was supported by the USDA NIFA Hatch Act (Regular Research Fund, ORE00218, ORE00218 A), and 10.13039/100000001National Science Foundation, United States (#1832170).

## CRediT authorship contribution statement

**Riley Prince:** Writing – review & editing, Writing – original draft, Validation. **Kai Roy:** Writing – original draft, Visualization. **Nathan Jesudason:** Writing – original draft, Software. **Marc Belinga:** Writing – original draft, Software. **Jacob Field:** Writing – original draft. **Dylan Heiesy:** Writing – original draft. **Aaron Arvidson:** Writing – original draft. **Torrey Menne:** Writing – original draft. **John Selker:** Supervision, Resources, Project administration, Funding acquisition. **Chet Udell:** Supervision, Resources, Project administration, Funding acquisition, Conceptualization.

## Declaration of competing interest

The authors declare the following financial interests/personal relationships which may be considered as potential competing interests: John Selker reports financial support was provided by USDA. Joh Selker reports financial support was provided by National Science Foundation. If there are other authors, they declare that they have no known competing financial interests or personal relationships that could have appeared to influence the work reported in this paper.
